# Photocatalytic synthesis of tetra-substituted furans promoted by carbon dioxide†

**DOI:** 10.1039/d1sc06403g

**Published:** 2021-12-06

**Authors:** Ya-Ming Tian, Huaiju Wang, Ritu, Burkhard König

**Affiliations:** Institute of Organic Chemistry, Faculty of Chemistry and Pharmacy, University of Regensburg 93040 Regensburg Germany Burkhard.Koenig@chemie.uni-regensburg.de

## Abstract

We report a simple protocol for the transition metal-free, visible-light-driven conversion of 1,3-diketones to tetra-substituted furan skeleton compounds in carbon dioxide (CO_2_) atmosphere under mild conditions. It was found that CO_2_ could be incorporated at the diketone enolic OH position, which was key to enabling the cleavage of a C–O bond during the rearrangement of a cyclopropane intermediate. This method allows for the same-pot construction of two isomers of the high-value tetra-substituted furan scaffold. The synthetic scope and preliminary mechanistic investigations are presented.

## Abstract 2

A CO_2_-promoted transition metal-free photocatalytic synthesis of tetra-substituted furan derivatives from 1,3-diketones as the only starting material.

## Graphical abstract

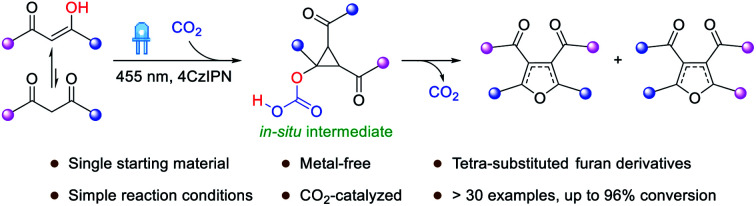

## Introduction

**Scheme 1:**
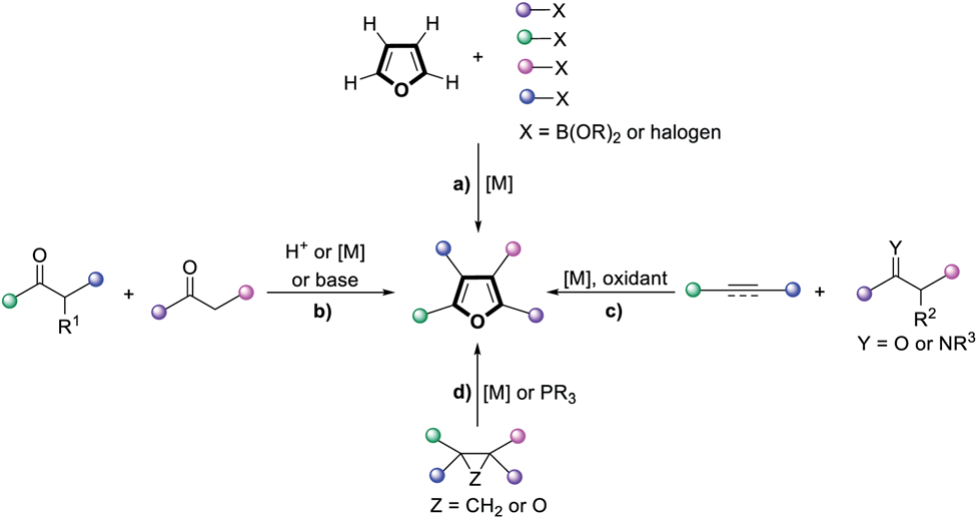
Approaches to access highly substituted furans *via*: (a) successive direct functionalization of furan C–H; (b) condensation; (c) annulation of unsaturated substrates with ketones or imines; (d) migratory cycloisomerization and rearrangements.

Polysubstituted furans are of great importance in the flavor and fragrance industry, pharmaceutical industry and materials chemistry, and are also valuable building blocks in organic synthesis.^1^ However, the preparation of such polysubstituted furans is often complicated and circuitous. While direct functionalization of the furan C–H positions is possible (Scheme 1a), regioselectivity challenges and detrimental reactivity of the furan ring itself hampered such approaches. Classical strategies to construct fully functionalized furans include the Feist–Benary reaction^2^ and Paal–Knorr condensation^3^ (Scheme 1b). Annulation of unsaturated substrates with ketones or imines^4^ (Scheme 1c) as well as other cross-coupling approaches^5^ could also afford polysubstituted furans, but migratory cycloisomerization^6^ and rearrangements^7^ are the most straightforward and convergent routes (Scheme 1d).^8^ Furthermore, current methods to access such furans typically require the use of metal catalysts and multiple components. As such, an atom-efficient, transition metal-free, organocatalytic protocol to synthesize tetra-substituted furans from a single precursor is highly sought after.

**Scheme 2:**
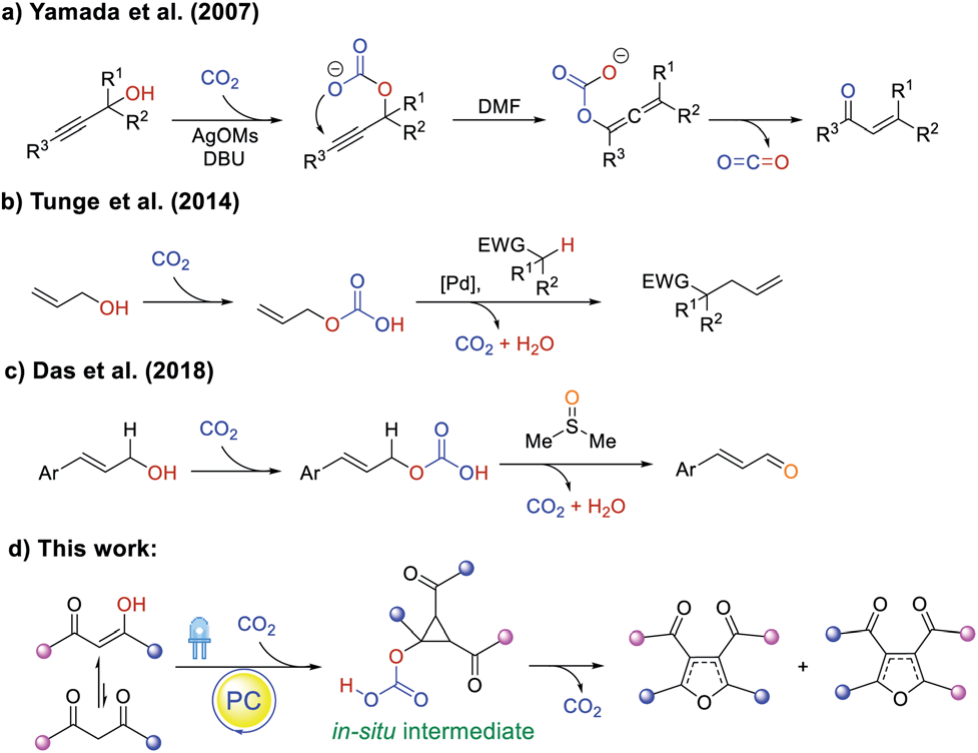
CO_2_-promoted organic transformations: (a) rearrangement of propargyl alcohols to unsaturated ketones; (b) cross-coupling using native allylic alcohol; (c) oxidation of allylic alcohols to unsaturated aldehydes. This work: (d) synthesis of tetra-substituted furans.

Carbon dioxide as a natural, abundant, inexpensive, easy-to-separate, and recyclable C1 building block has become the focus of recent research,^9–11^ but its capability to catalytically promote reactions has not been widely explored. Shell Oil Company first patented the utilization of CO_2_ to facilitate the synthesis of propionaldehydes in 1968,^12^ but only in 2007 was a CO_2_-catalyzed rearrangement of propargyl alcohols to unsaturated ketones reported by Yamada (Scheme 2a).^13^ Proceeding *via* a carbonate intermediate generated between CO_2_ and the propargyl alcohol, the reaction regenerates the gas upon formation of the product. Similarly, Tunge showed the use of CO_2_ to engage allylic OH groups, producing better leaving groups for cross-coupling (Scheme 2b),^11*g*^ and Das further reported the CO_2_-promoted oxidation of allylic alcohols to unsaturated aldehydes (Scheme 2c).^10*b*^ More recently, it was also demonstrated that CO_2_ can act on amine substrates to activate α-C–H bonds for intermolecular hydrogen atom transfer.^14,15^ Taken together, while the study of CO_2_-catalysis is still in its infancy, it offers new opportunities to perform organocatalysis that can complement traditional metal-based chemistry.

Therefore, we envisioned utilizing CO_2_ together with organic dyes to catalyze the cyclizations of 1,3-diketones^1*b*,16,17^ and subsequent cyclopropane rearrangement events^7^*en route* to valuable tetra-substituted furan products, enabled by the reversible interaction of CO_2_ with the enol forms that can provide favorable carbonate leaving groups.^10*b*,*c*,11*g*^ The optimization survey, substrate scope, and preliminary mechanistic studies of this transition metal-free, photocatalytic, and CO_2_-promoted furan synthesis strategy are presented herein.

## Results and discussion

We started our investigation by employing 1-phenyl-1,3-butanedione (1a) as the model substrate in the presence of CO_2_. At first, a range of commercially available organic photocatalysts and bases were screened (Table S1†). Gratifyingly, the desired tetra-substituted furans were produced by using 4CzIPN as the photocatalyst, *N*,*N*-dimethylformamide (DMF) as solvent, Cs_2_CO_3_ as a base, at 25 °C under 455 nm light irradiation, and in CO_2_ atmosphere. Surprisingly, two different tetra-substituted isomers can be formed in the same reaction system, giving 48% and 50% yield of products 1b and 1c, respectively (Table 1, entry 1). Temperature of the photocatalytic reaction seemingly has an effect on the regioselectivity between 1b and 1c (Table 1, entry 2). The yields of the products are influenced by the amount of base, with 1.5 equivalents of Cs_2_CO_3_ providing the best yields (Table S2†). We then screened a range of solvents, with DMF proving to be optimal (Table S3†). Light sources at different wavelengths were found to be similarly effective (Table S4†). However, when the CO_2_ atmosphere was replaced with N_2_, no desired products were formed (Table 1, entry 3). Only trace amounts of products 1b and 1c were detected when the reaction was carried out under air (Table 1, entry 4), indicating that CO_2_ is an indispensable component in this reaction. In the dark, no reaction occurred at either 25 °C or 60 °C (Table 1, entries 5 and 6), ruling out a base-catalyzed thermal pathway. Similarly, no detectable products were formed when the reaction was carried out in the absence of photocatalyst or base (Table 1, entries 7 and 8).

**Table 1:** Control experiments for the reaction of 1a

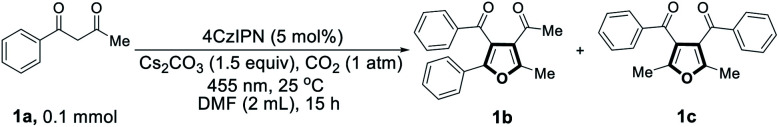			
Entry	Deviations from standard conditions	Yield of 1b^a^	Yield of 1c^a^
1	None	48%	50%
2	60 °C	24%	75%
3	N_2_ (1 atm) instead of CO_2_	n.d.	n.d.
4	Air (1 atm) instead of CO_2_	<1%	<1%
5	No light, 25 °C	n.d.	n.d.
6	No light, 60 °C	n.d.	n.d.
7	No 4CzIPN	n.d.	n.d.
8	No Cs_2_CO_3_	n.d.	n.d.

a Yields were determined by GC-MS analysis against an internal standard and are the average of two runs; n.d., product was not detected.

**Scheme 3:**
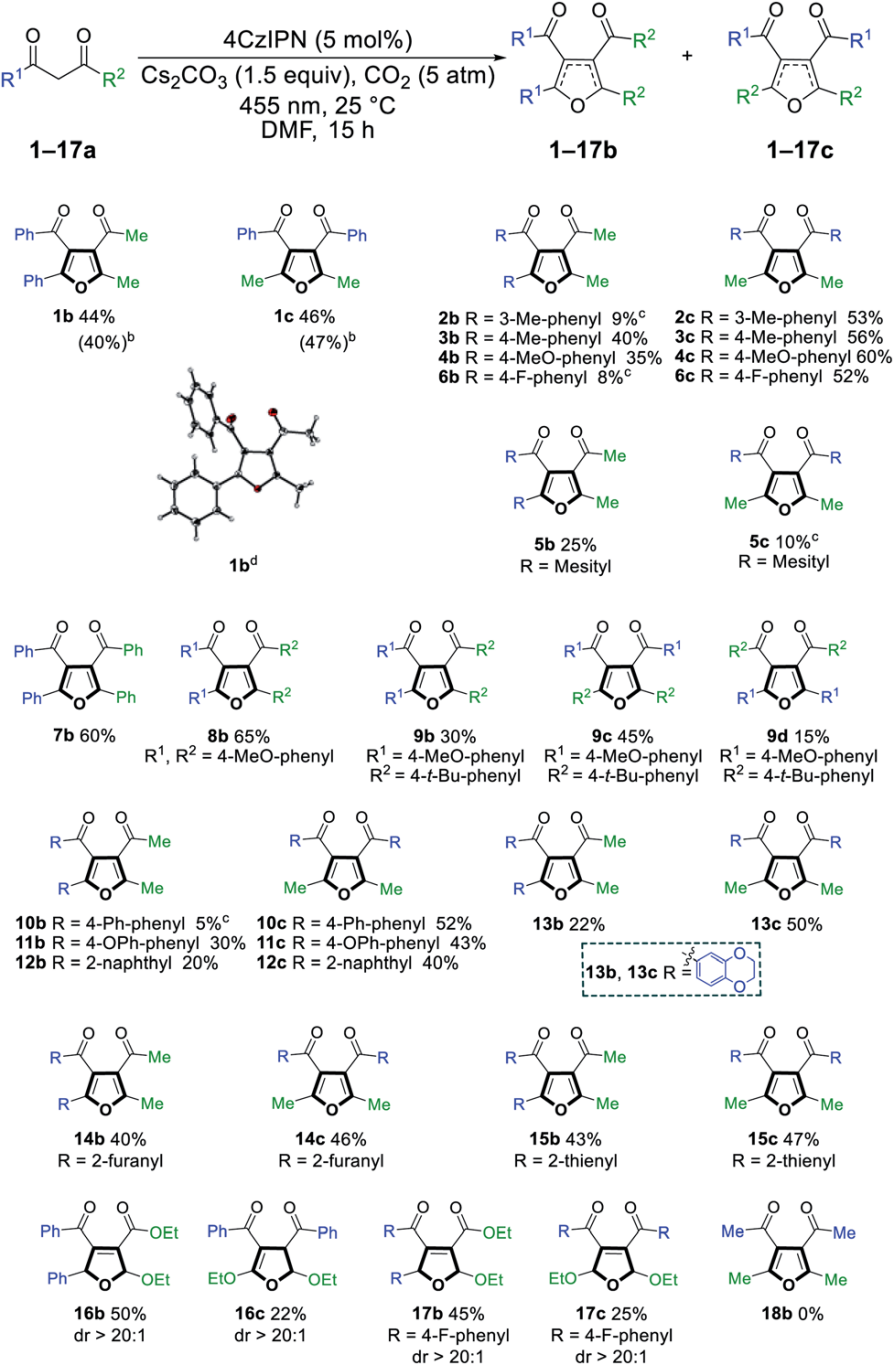
Substrate scope of tetra-substituted furan from 1,3-diketone. ^a^Reaction conditions: 1,3-diketone (0.5 mmol), 4CzIPN (0.025 mmol), Cs_2_CO_3_ (0.75 mmol), CO_2_ (5 atm) in DMF (5 mL) at 25 °C under irradiation with a 455 nm LED for 15 h. Reported yields are isolated yields unless stated otherwise. ^b^Isolated yields of gram-scale reaction. ^c^Yields were determined by GC-MS analysis against an internal standard and are the average of two runs. Products were not isolated due to their low yields. ^d^Crystal structure of 1b as determined by X-ray crystallography at 123 K (see ESI†).

With the optimized reaction conditions in hand, we evaluated the scope and limitations of this method (Scheme 3). Both electron-rich and electron-deficient phenyl rings were well tolerated (2–4, 6), but a mesityl group provided deleterious steric hindrance (5). Diaryl-1,3-diketones reacted smoothly to afford the desired products (7–9). Notably, the reaction of 9a could produce three different products when the two phenyl groups contain different substituents. Other benzene systems such as biphenyl (10), phenyl ether (11), naphthalene (12) and benzodioxane (13) were also compatible with our reaction conditions. The preference for the symmetric regioisomers in 2, 6, and 10 cannot be explained at the current stage. Furthermore, this method was successfully extended to heterocyclic substituents, such as furan and thiophene (14, 15). Surprisingly, when the 1,3-diketone motif was replaced with 3-oxo-ester, the reaction yielded 2,5-dihydrofurans (16b, 17) and unsymmetric 2,3-dihydrofurans (16c, dr > 20 : 1) that resisted regioisomerization and further oxidation to furans even after extensive exposure to air (*vide infra*). We speculate that the less hydridic C2 hydrogens and the overall less electron-rich conjugation systems made 16 and 17 products stable to oxidation, while the electronic effects of the benzene ring could affect the equilibrium between different dihydrofuran isomers (16c*vs.*17c). Unfortunately, no reaction occurred with acetylacetone 18a (*vide infra*). The structure of product 1b was unambiguously confirmed *via* single crystal X-ray analysis. A gram-scale reaction of 1a was also conducted under standard conditions, giving 40% and 47% yield of products 1b and 1c, respectively.

**Scheme 4:**
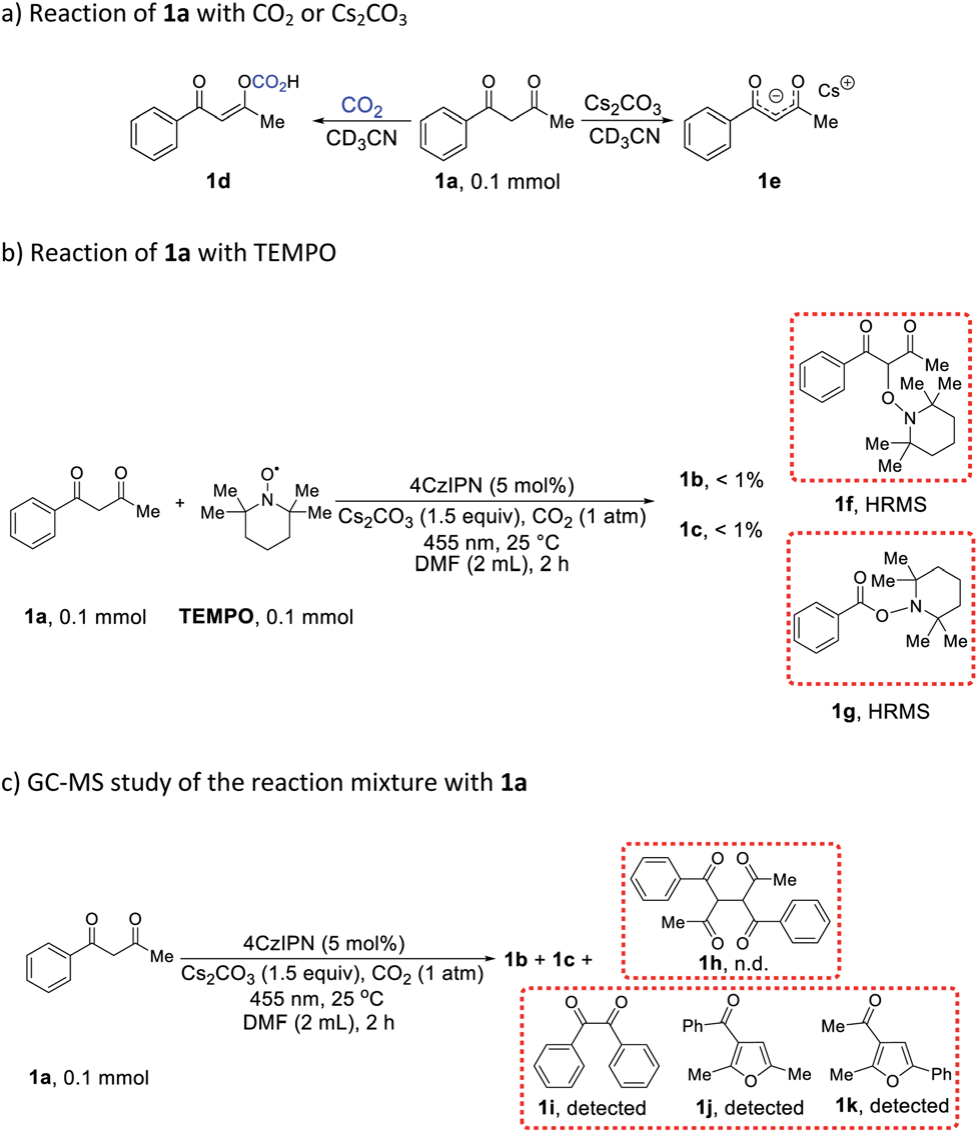
Mechanistic studies.

In order to gain insight into the aforementioned transformation, a series of mechanistic studies was conducted. To confirm whether the reaction proceeds *via* a radical process, *in situ* electron paramagnetic resonance (EPR) spectra of different reactions were recorded (Table S6† and Fig. S1–S14†). No signal was observed for the mixture of 1a, 4CzIPN, and CO_2_, either with or without blue light irradiation (Table S6,† entries 1 and 2). Similarly, the mixture of 1a, Cs_2_CO_3_, and CO_2_ did not show any signal with or without blue light irradiation (Table S6,† entries 3 and 4). In addition, there was no EPR signal when mixing 4CzIPN, Cs_2_CO_3_, and CO_2_ (Table S6,† entries 5 and 6), or 1a with CO_2_ alone (Table S6,† entries 7 and 8). However, a significant EPR signal was observed when a composition of 1a, 4CzIPN, Cs_2_CO_3_, and CO_2_ was irradiated (Table S6,† entry 10). When the CO_2_ atmosphere was replaced with N_2_, the same signal was still present (Table S6,† entry 12), indicating that CO_2_ did not participate in the generation of this radical species. To identify the origin of the radical resonance signal, we employed 7a instead of 1a in the EPR measurement, obtaining an identical signal (Fig. S7–S9†). Speculating that the signal arose from a 4CzIPN radical anion, we recorded the EPR spectrum of this species by adding NEt_3_ as an electron donor to a solution of 4CzIPN (Fig. S14†), which gave the same resonance signal as did the catalysis system.^18^ Accordingly, we concluded that 1a, 4CzIPN, Cs_2_CO_3_ and light irradiation together resulted in the formation of 4CzIPN radical anion. Then, the redox behavior of 1a and its anion 1a^−^ were investigated. For 1a, no oxidation peak was observed within the electrochemical window of DMF as solvent (Fig. S15†). The anion of 1a, synthesized independently, showed an irreversible one-electron oxidation (*E*_ox_ = −0.09 V *vs.* SCE), which is able to reduce the photoexcited state of 4CzIPN (*E*_1/2_[PC*/PC^−^] = +1.35 V *vs.* SCE)^19^ to provide the EPR-observed radical anion and a transient alkyl radical of 1a. Stern–Volmer luminescence quenching experiments (Fig. S17 and S18†) revealed efficient quenching of photoexcited 4CzIPN* upon addition of 1a and Cs_2_CO_3_ (*K*_SV_ = 113 M^−1^). *In situ* NMR studies demonstrated that 1a react with CO_2_ to produce compound 1d (Scheme 4a and Fig. S20–S31,† observing the carbonic acid carbon peak), and naturally, Cs_2_CO_3_ can deprotonate 1a to give salt 1e (Scheme 4a and Fig. S32†). While no change was observed when 1a or 1d was subjected to irradiation together with 4CzIPN (Fig. S33–S40†), the ^1^H spectrum of 1e was altered under these conditions (Fig. S41–S44†).

When the reaction of 1a was carried out in the presence of 1 equiv. 2,2,6,6-tetramethylpiperidinyloxyl (TEMPO) as a radical trap, the desired reactivity was almost completely shut down, while adducts 1f and 1g were formed, hinting at the presence of C(sp^3^)-centered alkyl radicals generated from 1a, as well as benzoyl radicals (Scheme 4b). The detection of benzil 1i lends further support to the formation of benzoyl radicals in the course of the reaction (Scheme 4c). Correspondingly, tri-substituted furans 1j and 1k were also present in the reaction mixture (Scheme 4c). It is worth noting that benzoyl radical PhC(O)˙ (*E*_red_ = −1.13 V *vs.* SCE)^20^ is capable of oxidizing 4CzIPN radical anion (*E*_1/2_[PC/PC^−^] = −1.21 V *vs.* SCE),^19^ whereas CH_3_C(O)˙ (*E*_red_ = −1.75 V *vs.* SCE)^20^ is not, giving a possible explanation to the lack of reactivity with acetylacetone 18a. In addition, the dimer (1h) of starting material 1a was not formed in any appreciable amount (Scheme 4c), suggesting that alkyl radicals originating from 1a did not undergo a simple dimerization. Light “on–off” experiments indicate that the reaction needs continuous light irradiation to proceed (Fig. S19†), ruling out a radical-chain mechanism.

**Scheme 5:**
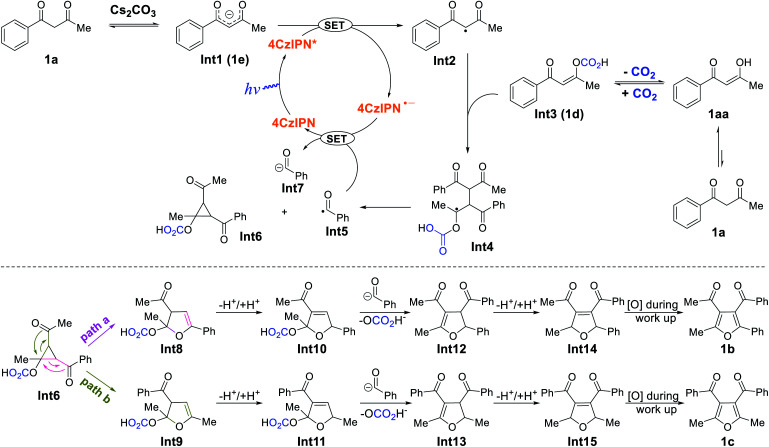
Proposed reaction mechanism.

Based on the above observations and previous studies,^7^ a mechanism for the CO_2_-promoted photocatalytic activation of 1,3-diketones to afford tetra-substituted furans is proposed (Scheme 5). The starting material 1a is deprotonated by Cs_2_CO_3_ to generate enolate 1e, which is oxidized by photoexcited 4CzIPN* through a single electron transfer process, forming 4CzIPN˙^−^ and radical Int2. In addition, 1a also equilibrates with CO_2_ to form adduct 1d, which reacts with radical Int2, giving the dimeric Int4. Subsequently, Int4 ejects benzoyl radical PhC(O)˙ (Int5) to form acylcyclopropane Int6*via* an intramolecular cyclization process (see ESI† section XII for discussion). Density functional theory (DFT) calculations reveal that the formation of Int5 and Int6 from Int4 (Δ*G* = +10 kcal mol^−1^, see ESI† section XIII) is most likely the rate-determining step. Thereafter, benzoyl radical Int5 oxidizes 4CzIPN˙^−^ to close the catalytic cycle and produce benzoyl anion Int7. The ring-opening rearrangement of Int6 results in furanoid structures, Int8 and Int9, *via* two different pathways.^7*c*,*d*^ Computations suggest that the ring-opening processes are exergonic and favorable. The protonation and deprotonation of Int8 and Int9 lead to the formation of conjugated Int10 and Int11. Cleavage of the C–O bonds and the accompanying nucleophilic attack by Int7 generate tetra-substituted 2,3-dihydrofurans Int12 and Int13 (isolated as product 16c). We postulated that 1j and 1k detected by GC-MS came from the direct C–O cleavage of Int10 and Int11 with further aromatization in air. Intermediates Int12 and Int13 undergo additional protonation and deprotonation to afford the more conjugated Int14 and Int15 (isolated as products 16b, 17b, 17c). At last, aromatization-driven oxidation processes yield the desired tetra-substituted furans.

## Conclusion

In conclusion, we have developed an unusual, transition metal-free, CO_2_-promoted, visible-light-induced photocatalytic synthesis of highly substituted furan derivatives using 1,3-diketones as the only starting material. Mechanistic investigations indicated that CO_2_ was catalytically incorporated in order to create a better leaving group from the enolic OH group. The reaction proceeds under mild conditions *via* diketone radical additions and acylcyclopropane rearrangements, leading to the formation of two isomeric but differently substituted furan products. From 3-oxo-ester starting materials, partially hydrogenated furan scaffolds could also be obtained. This protocol expands the scope of the photocatalytic *de novo* synthesis of heterocyclic compounds as well as the catalytic use of CO_2_ as a reaction promoter.

## Data availability

All experimental, computational, and crystallographic data are available in the ESI.†

## Author contributions

Y.-M. T. and B. K. conceived the project. Y.-M. T. performed and analyzed the experiments. H. W. performed the DFT and CBS calculations. R. synthesized some materials. Y.-M. T., H. W., and B. K. prepared the manuscript. All authors discussed the results.

## Conflicts of interest

There are no conflicts to declare.

## Supplementary Material

SC-013-D1SC06403G-s001

SC-013-D1SC06403G-s002
