# Supplementary material for: Photocatalytic synthesis of tetra-substituted furans promoted by carbon dioxide
Source: Chem Sci. 2021 Dec 6;13(1):241–6. doi: 10.1039/d1sc06403g (PMC8694347; doi:10.1039/d1sc06403g)
Supplement: SC-013-D1SC06403G-s001 [file SC-013-D1SC06403G-s001.pdf]

Electronic Supplementary Material (ESI) for Chemical Science.
This journal is © The Royal Society of Chemistry 2021

Supporting Information

Photocatalytic Synthesis of Tetra-Substituted Furans Promoted by Carbon Dioxide

Ya-Ming Tian, Huaiju Wang, Ritu, Burkhard König*

Institute of Organic Chemistry, Faculty of Chemistry and Pharmacy, University of Regensburg, 93040 Regensburg, Germany

E-mail: Burkhard.Koenig@chemie.uni-regensburg.de

Table of contents

I. General information 3

II. Experimental procedures 6

III. Optimization of the reaction conditions 7

IV. EPR investigations 10

V. Cyclic voltammetry 19

VI. Stern-Volmer luminescence quenching experiments 21

VII. Light "on-off" experiments 22

VIII. *In-situ* NMR investigations 23

IX. NMR and HRMS data of the products 42

X. NMR spectra of the products 53

XI. Single-crystal X-ray diffraction analysis of product 1b 84

XII. Possible mechanism for the transformation of Int4 to Int5 and Int6 86

XIII. Energy computations of key intermediates 88

XIV. References 114

I. General information

All reagents were purchased from Alfa-Aesar, TCI, Sigma-Aldrich, ABCR, Acros or Fluorochem, and were checked for purity by GC-MS and/or ^{1}H NMR spectroscopy and used as received. Unless otherwise noted, all manipulations were performed using standard Schlenk manifold technique. Extra-dry anhydrous *N*,*N*-dimethylformamide and acetonitrile were purchased from Acros.

All NMR spectra were recorded at ambient temperature using Bruker Avance 300 NMR (^{1}H, 300 MHz; ^{13}C{^{1}H}, 75 MHz), or Bruker Avance 400 NMR (^{1}H, 400 MHz; ^{13}C{^{1}H}, 101 MHz; ^{19}F, 376 MHz) spectrometers.[1] ^{1}H NMR chemical shifts are reported relative to TMS and were referenced via residual proton resonances of the corresponding deuterated solvent ($CDCl_3$: 7.26 ppm, CD_3CN: 1.94 ppm) whereas ^{13}C{^{1}H} NMR spectra are reported relative to TMS and were referenced *via* the carbon signals of the deuterated solvent ($CDCl_3$: 77.16 ppm, CD_3CN: 1.32, 118.26 ppm). ^{19}F NMR chemical shifts are reported relative to $CFCl_3$ as external standard.[2] All ^{13}C and ^{19}F NMR spectra were broad-band ^{1}H decoupled. Coupling constants *J* are given in Hertz (Hz). Abbreviations used for signal multiplicity: ^{1}H-NMR: b = broad, s = singlet, d = doublet, t = triplet, q = quartet, p = quintet, and m = multiplet.

High-resolution mass spectra (HRMS) were obtained from the central analytic mass spectrometry facilities of the Faculty of Chemistry and Pharmacy, Regensburg University, and are reported according to the 2013 IUPAC recommendations. All mass spectra were recorded on a Finnigan MAT 95, Thermo Quest Finnigan TSQ 7000, Finnigan MATSSQ 710 A or Agilent Q TOF 6540 UHD instrument. Gas chromatography (GC) measurements were performed on an Agilent GC 7890. Data acquisition and evaluation was done with Agilent ChemStation Rev.C.01.04. Analytical TLC was performed on silica gel-coated alumina plates (MN TLC sheets ALUGRAM ® Xtra SIL G /UV254). Visualization was done by UV light (254 or 366 nm). If necessary, potassium permanganate was used for chemical staining. Purification by column chromatography was performed with silica gel 60 M (40-63 µm, 230-440 mesh, Merck) on a Biotage® Isolera TM Spektra One device. All photocatalytic reactions were performed with OSRAM Oslon SSL 80 royal blue LEDs (λ = 455 nm ± 15 nm, 3.5 V, 700 mA). The samples were irradiated with an LED through the bottom side and cooled from the side using custom-made aluminum water-cooling blocks connected to a thermostat. Gram-scale

reactions were performed in a glass tube photochemical reactor setup irradiated from the outside. The reaction mixture and the LED cooling block were temperature-controlled at 25 °C. CO_2 was bubbled continuously through the reaction mixture in large-scale reactions.

UV-Vis and fluorescence measurements were performed with a Varian Cary 100 UV/Vis spectrophotometer and FluoroMax 4 spectrofluorometer, respectively. CV measurements were taken on a three-electrode potentiostat galvanostat PGSTAT302N from Metrohm Autolab by using a glassy carbon working electrode, a platinum wire counter electrode, a silver wire as a reference electrode. The voltammograms were taken at room temperature in a degassed DMF or MeCN solution ([n-Bu_4NBF_4] = 0.1 M, [substrate] = 1 mM, ferrocene as the internal standard) under Argon atmosphere. The scan rate was 0.1 V/s. Potentials vs. SCE were reported according to $E_{SCE} = E_{Fc/Fc+}$ + 0.38 V.

For the single-crystal X-ray diffraction, a clear colourless prism-shaped crystal with dimensions 0.13 × 0.09 × 0.04 mm^3 was mounted. Data were collected using a XtaLAB Synergy R, DW system, HyPix-Arc 150 diffractometer operating at T = 123.00(10) K. Data were measured using ω scans using Cu K_α radiation. The diffraction pattern was indexed and the total number of runs and images was based on the strategy calculation from the program CrysAlisPro (Rigaku, V1.171.41.93a, 2020). The maximum resolution that was achieved was Θ = 73.045° (0.81 Å). The diffraction pattern was indexed and the total number of runs and images was based on the strategy calculation from the program CrysAlisPro (Rigaku, V1.171.41.93a, 2020). The unit cell was refined using CrysAlisPro (Rigaku, V1.171.41.93a, 2020) on 11229 reflections, 68% of the observed reflections. Data reduction, scaling and absorption corrections were performed using CrysAlisPro (Rigaku, V1.171.41.93a, 2020). The final completeness is 99.90 % out to 73.045° in Θ. A gaussian absorption correction was performed using CrysAlisPro 1.171.41.93a (Rigaku Oxford Diffraction, 2020) Numerical absorption correction based on gaussian integration over a multifaceted crystal model Empirical absorption correction using spherical harmonics, implemented in SCALE3 ABSPACK scaling algorithm. The absorption coefficient μ of this material is 0.730 mm^{-1} at this wavelength (λ = 1.54184Å) and the minimum and maximum transmissions are 0.831 and 1.000. The structure was solved and the space group $P2_1/n$ (# 14) determined by the ShelXT 2018/2 (Sheldrick, 2018) structure solution program

using using dual methods and refined by full matrix least squares minimisation on $\boldsymbol{F}^2$ using version 2018/3 of ShelXL 2018/3 (Sheldrick, 2015).[3,4] All non-hydrogen atoms were refined anisotropically. Hydrogen atom positions were calculated geometrically and refined using the riding model. Hydrogen atom positions were calculated geometrically and refined using the riding model. Diamond[5] software was used for graphical representation. Crystal data and experimental details are listed in Figure S110 and Table S7. Full structural information has been deposited with the Cambridge Crystallographic Data Centre. CCDC-2113371. EPR measurements at X-band (9.38 GHz) were carried out using a Bruker ELEXSYS E580 CW EPR spectrometer equipped with an Oxford Instruments helium cryostat (ESR900) and a MercuryiTC temperature controller.

II. Experimental procedures

Method A

Unless specified otherwise, a mixture of 1,3-diketone (0.1 mmol), 4CzIPN (0.005 mmol), Cs_2CO_3 (0.15 mmol) was added to an oven-dried 5 mL snap vial equipped with a magnetic stirring bar. The capped vial was evacuated and back filled with CO_2 for three times and dry DMF (2 mL) was added to the vial by syringe. The solution was then bubbled with CO_2 for 5 min. Then, the cap was sealed with parafilm. The reaction mixture was stirred and irradiated with a 455 nm LED at 25 °C for 15 h, then diluted with dichloromethane (DCM, 2 mL) and filtered through a plug of celite (Ø 3 mm × 8 mm). After addition of *n*-dodecane (0.2 mmol) as an internal standard for calibration, the product yield was determined by GC-MS.

Method B

Unless specified otherwise, a mixture of 1,3-diketone (0.5 mmol), 4CzIPN (0.025 mmol), Cs_2CO_3 (0.75 mmol) was added to an oven-dried 10 mL snap vial equipped with a magnetic stirring bar. The capped vial was evacuated and back filled with CO_2 for three times and dry DMF (5 mL) was added to the vial by syringe. The solution was then bubbled with CO_2 for 5 min. Then, CO_2 (24 mL) was injected by syringe into the reaction system and the cap was sealed with parafilm. The reaction mixture was stirred and irradiated with a 455 nm LED at 25 °C for 15 h. The reaction was then quenched with saturated brine (5 mL) and extracted with EtOAc (10 mL × 3). The combined organic phase was then washed with H_2O (10 mL) and brine, dried over sodium sulfate, and concentrated under vacuum. The residue was purified by silica gel flash chromatography to give the desired product.

III. Optimization of the reaction conditions

Table S1. Screening of different photocatalysts and bases [a]

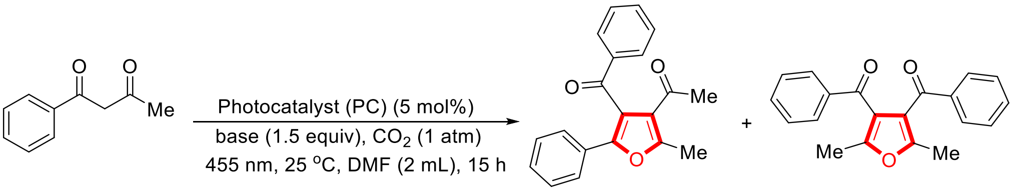

1a, 0.1 mmol **1b** **1c**

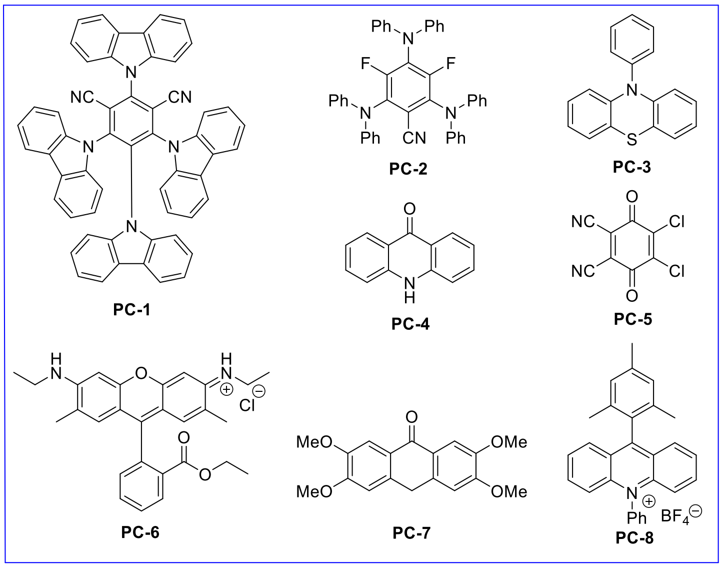

Entry	PC	Base	Conversion of 1a (%)	Yield of 1b (%)	Yield of 1c (%)
1	**PC-1**	Cs_2CO_3	100	48	50
2	**PC-2**	Cs_2CO_3	100	47	51
3	**PC-3**	Cs_2CO_3	49	13	25
4	**PC-4**	Cs_2CO_3	90	30	40
5	**PC-5**	Cs_2CO_3	35	12	15
6	**PC-6**	Cs_2CO_3	17	4	9
7	**PC-7**	Cs_2CO_3	28	9	9
8	**PC-8**	Cs_2CO_3	52	12	32
9	**PC-1**	K_2CO_3	70	16	40
10	**PC-1**	CsF	38	9	24
11	**PC-1**	Na_2HPO_4	10	n.d.	n.d.
12	**PC-1**	KBr	0	n.d.	n.d.

13	**PC-1**	$NaHCO_3$	0	n.d.	n.d.
14	**PC-1**	DABCO	0	n.d.	n.d.
15	**PC-1**	NaF	0	n.d.	n.d.

[a]Yields were determined by GC-MS analysis and are the averages of two runs; n.d., product was not detected.

Table S2. Reaction performance at different Cs_2CO_3 loadings[a]

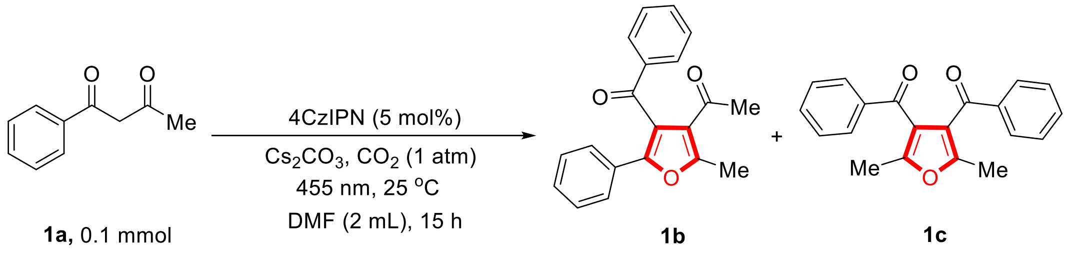

Entry	Amount of Cs_2CO_3 (mmol)	Conversion of 1a (%)	Yield of 1b (%)	Yield of 1c (%)
1	0.05 (0.5 equiv)	82	38	42
2	0.1 (1 equiv)	83	39	43
3	0.15 (1.5 equiv)	100	48	50
4	0.2 (2 equiv)	91	42	48

[a]The yields were determined by GC-MS analysis and are averages of two runs.

Table S3. Screening of different solvents[a]

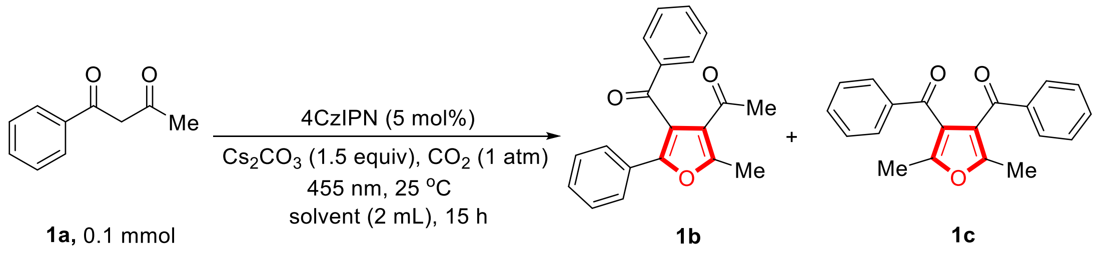

Entry	Solvent	Yield of 1b (%)	Yield of 1c (%)
1	DMF	48	50
2	CH_3CN	30	43
3	THF	20	30
4	Toluene	trace	trace

[a]Yields were determined by GC-MS analysis and are the averages of two runs.

Table S4. Screening of different light sources[a]

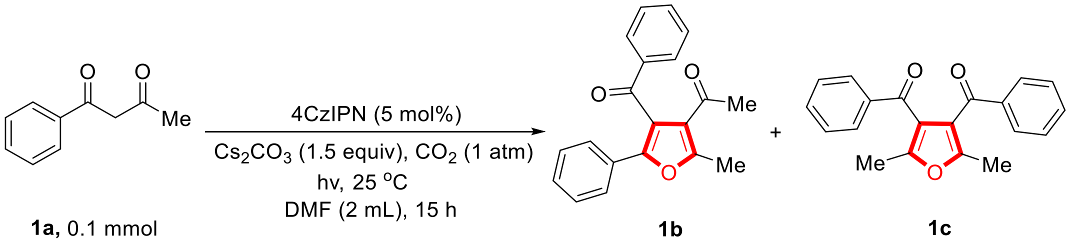

Entry	Light source	Yield of 1b (%)	Yield of 1c (%)
1	455 nm	48	50
2	400 nm	49	50
3	365 nm	47	50

[a]Yields were determined by GC-MS analysis and are the averages of two runs.

Table S5. Reaction performance at different CO_2 pressure[a]

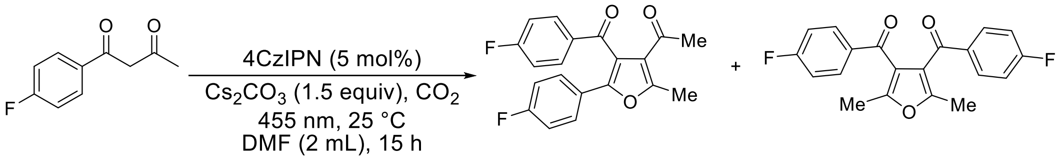

Entry	Pressure of CO_2	Yield of **6b**	Yield of **6c**
1	1 atm	3%	15%
2	5 atm	8%	56%
3	7 atm	8%	57%

[a]Yields were determined by GC-MS analysis and are the averages of two runs.

IV. EPR investigations

Table S6. EPR studies of different reactions

Entry	1a	4CzIPN	Cs_2CO_3	CO_2	N_2	hv	EPR signal
1	Yes	Yes	No	Yes	No	Yes	silent
2	Yes	Yes	No	Yes	No	No	silent
3	Yes	No	Yes	Yes	No	Yes	silent
4	Yes	No	Yes	Yes	No	No	silent
5	No	Yes	Yes	Yes	No	No	silent
6	No	Yes	Yes	Yes	No	Yes	silent
7	Yes	No	No	Yes	No	No	silent
8	Yes	No	No	Yes	No	Yes	silent
9	Yes	Yes	Yes	Yes	No	No	silent
10	Yes	Yes	Yes	Yes	No	Yes	active
11	Yes	Yes	Yes	No	Yes	No	silent
12	Yes	Yes	Yes	No	Yes	Yes	active

In order to confirm whether the reaction proceeds via a radical process, *in-situ* EPR spectra of different mixtures were recorded. No EPR signal was observed for the mixture of **1a**, CO_2 and 4CzIPN without (Figure S1) and with irradiation (Figure S2).

a) EPR of the mixture of **1a** and 4CzIPN without irradiation:

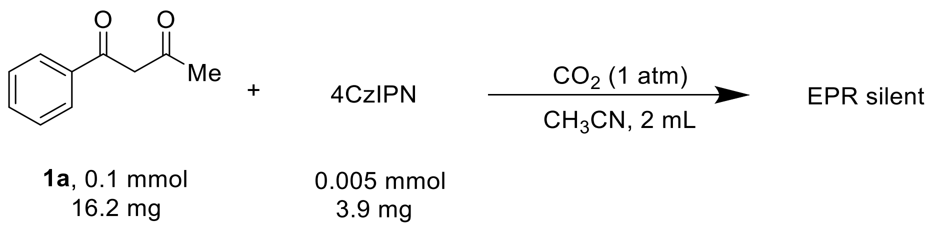

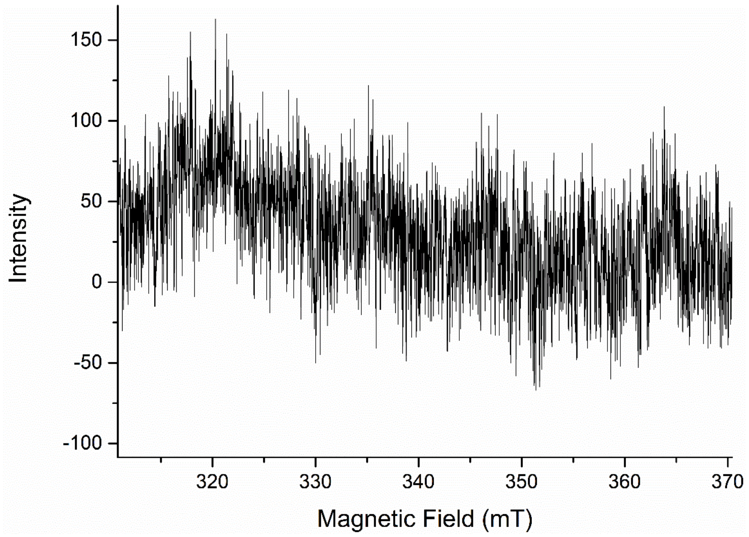

Figure S1. *In-situ* EPR spectrum of **1a** and 4CzIPN under 1 atm CO_2 in CH_3CN without irradiation.

b) EPR of the mixture of **1a** and 4CzIPN under 455 nm light irradiation:

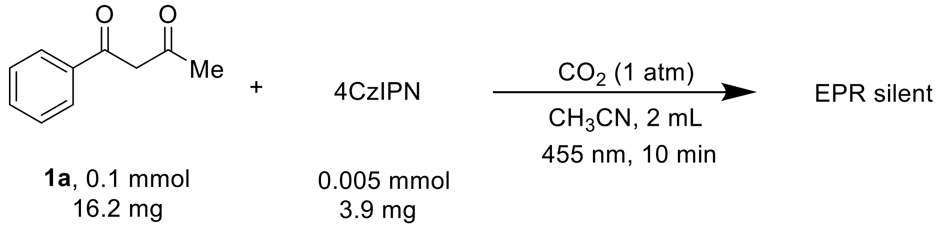

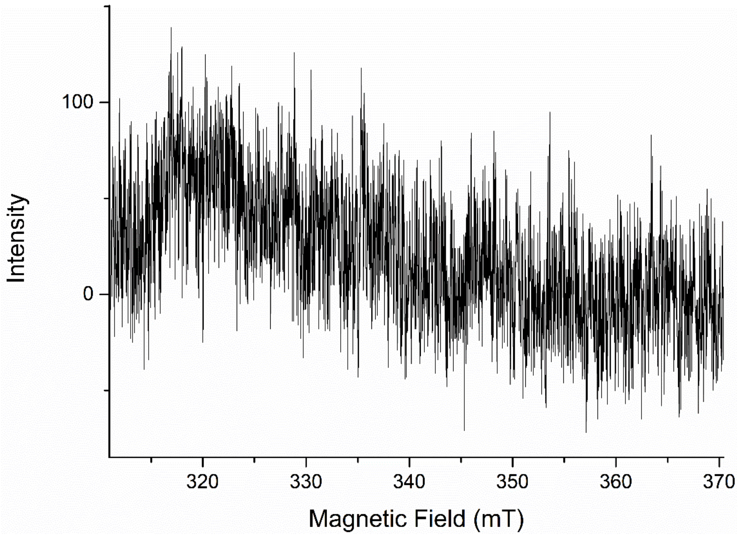

Figure S2. *In-situ* EPR spectrum of **1a** and 4CzIPN under 1 atm CO_2 in CH_3CN under 455 nm light irradiation for 10 min.

No EPR signal was observed for the mixture of **1a**, CO_2 and Cs_2CO_3 without (Figure S3) and with irradiation (Figure S4).

c) EPR of the mixture of **1a** and Cs_2CO_3 without irradiation:

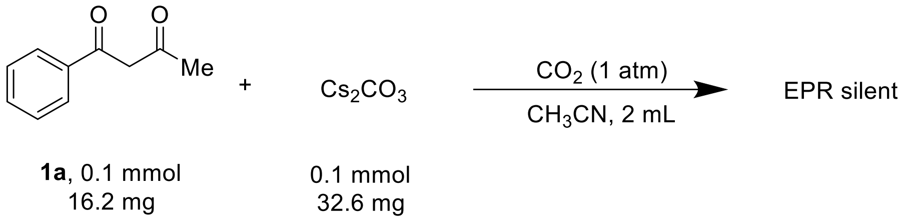

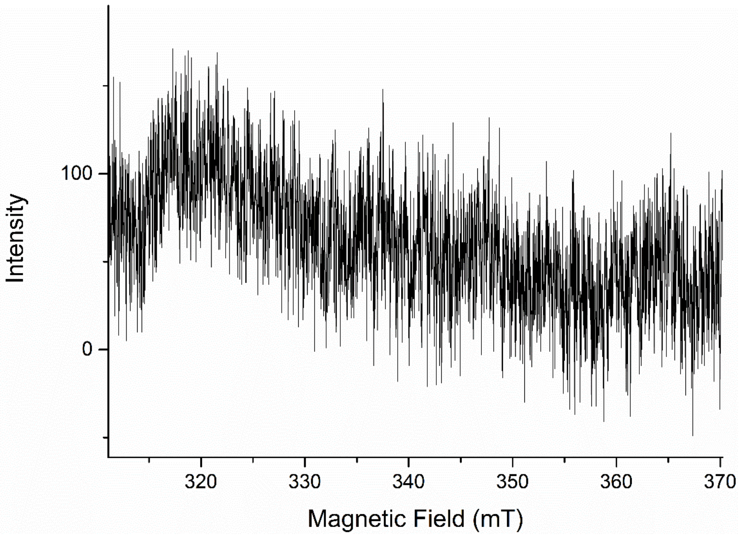

Figure S3. *In-situ* EPR spectrum of **1a** and Cs_2CO_3 under 1 atm CO_2 in CH_3CN without irradiation.

d) EPR of the mixture of **1a** and Cs_2CO_3 under 455 nm light irradiation:

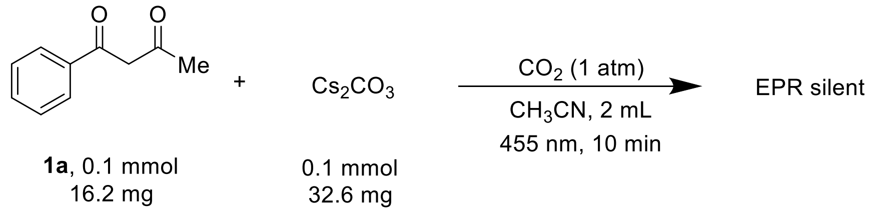

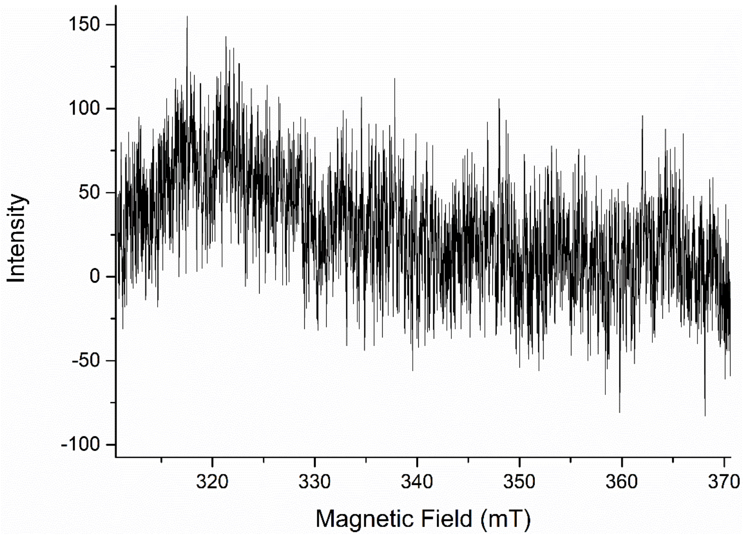

Figure S4. *In-situ* EPR spectrum of **1a** and Cs_2CO_3 under 1 atm CO_2 in CH_3CN under 455 nm light irradiation for 10 min.

No EPR signal was observed for the mixture of **1a**, CO_2, 4CzIPN and Cs_2CO_3 without (Figure S5) irradiation. However, a clear ERP signal was detected after 5 min irradiation (Figure S6), confirming the existence of radicals. We recorded the EPR spectra for the same sample at different irradiation times, which showed that the intensity of the signal decreased gradually over time (Figure S6).

e) EPR of the mixture of **1a**, 4CzIPN and Cs_2CO_3 without irradiation:

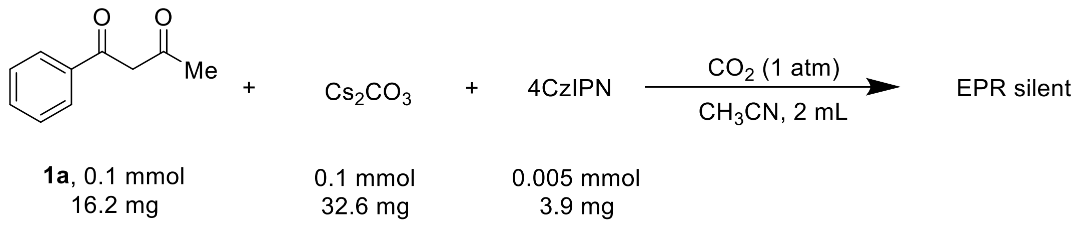

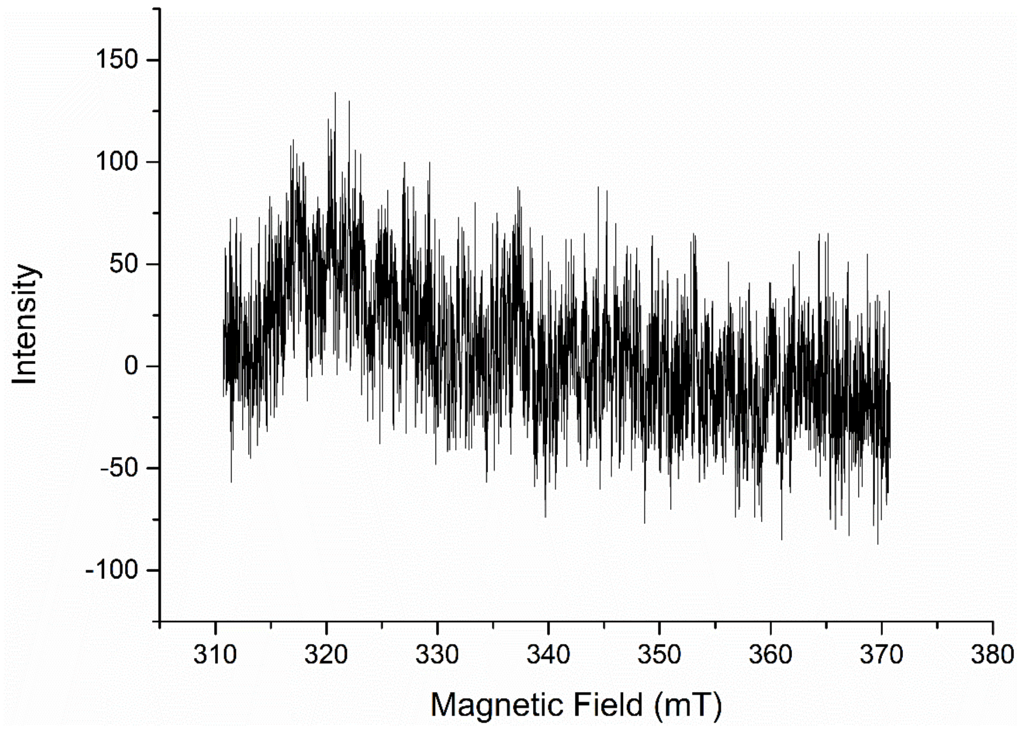

Figure S5. *In-situ* EPR spectrum of **1a**, 4CzIPN and Cs_2CO_3 under 1 atm CO_2 in CH_3CN without irradiation.

f) EPR of the mixture of **1a**, 4CzIPN and Cs_2CO_3 under 455 nm light irradiation:

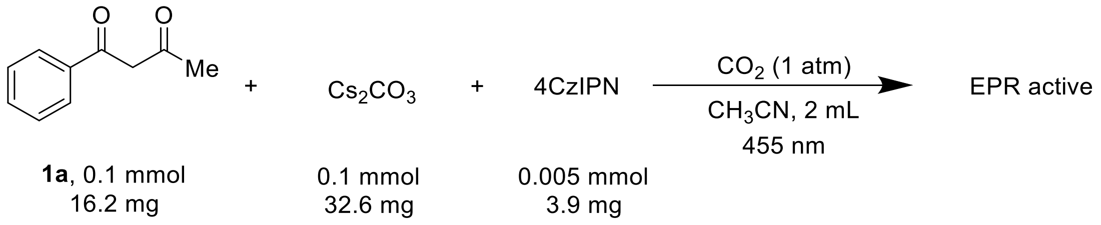

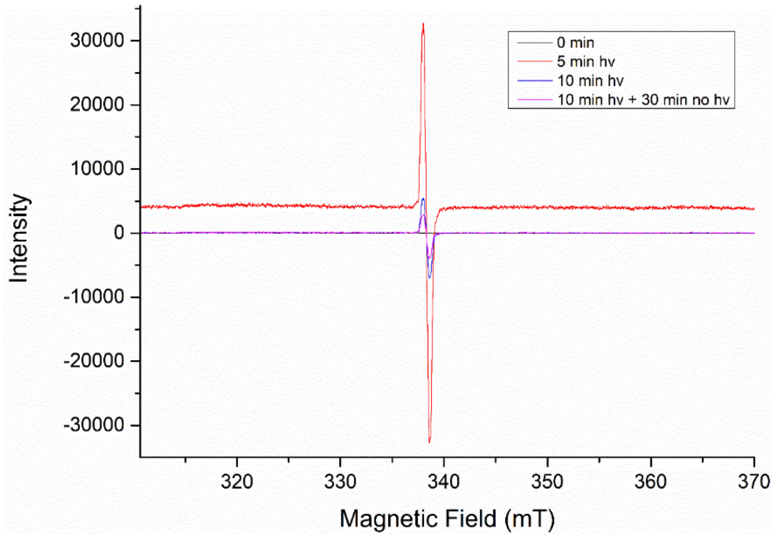

Figure S6. *In-situ* EPR spectra of **1a**, 4CzIPN and Cs_2CO_3 under 1 atm CO_2 in CH_3CN under 455 nm light irradiation for 0 min, 5 min, 10 min and 30 min in the dark after 10 min irradiation, respectively.

g) EPR of the mixture of **7a**, 4CzIPN and Cs_2CO_3 without irradiation:

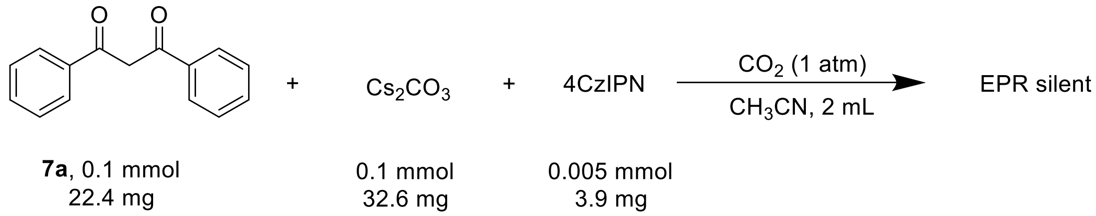

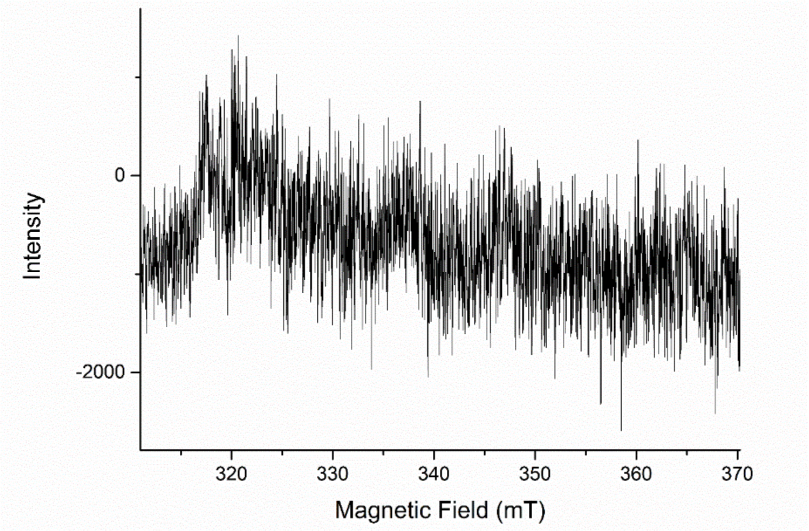

Figure S7. *In-situ* EPR spectrum of **7a**, 4CzIPN and Cs_2CO_3 under 1 atm CO_2 in CH_3CN without irradiation.

h) EPR of the mixture of **7a**, 4CzIPN and Cs_2CO_3 under 455 nm light irradiation:

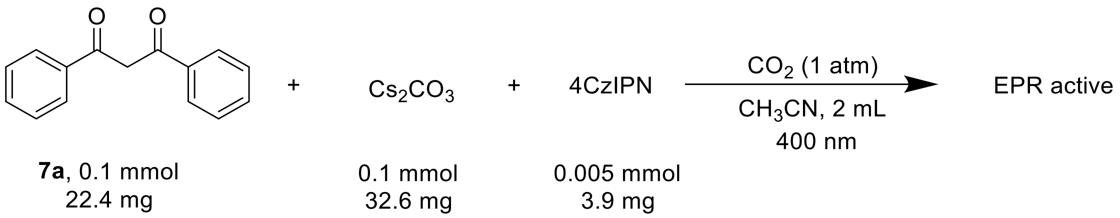

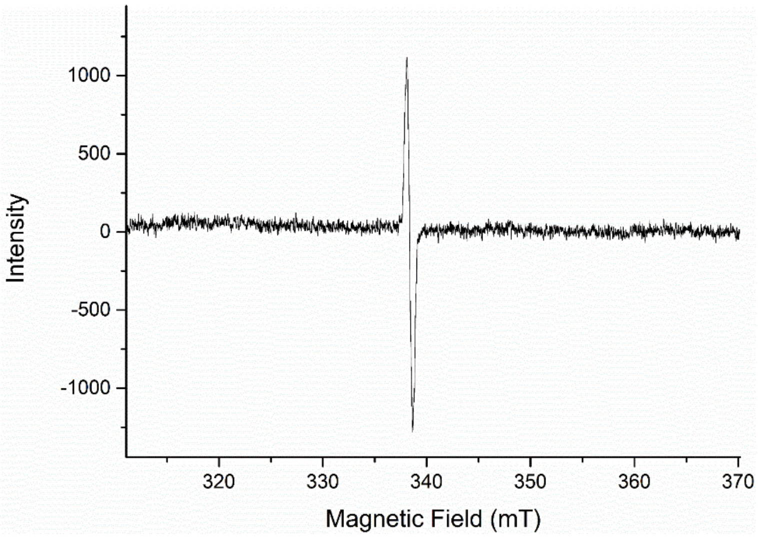

Figure S8. *In-situ* EPR spectrum of **7a**, 4CzIPN and Cs_2CO_3 under 1 atm CO_2 in CH_3CN under 455 nm light irradiation for 5 min.

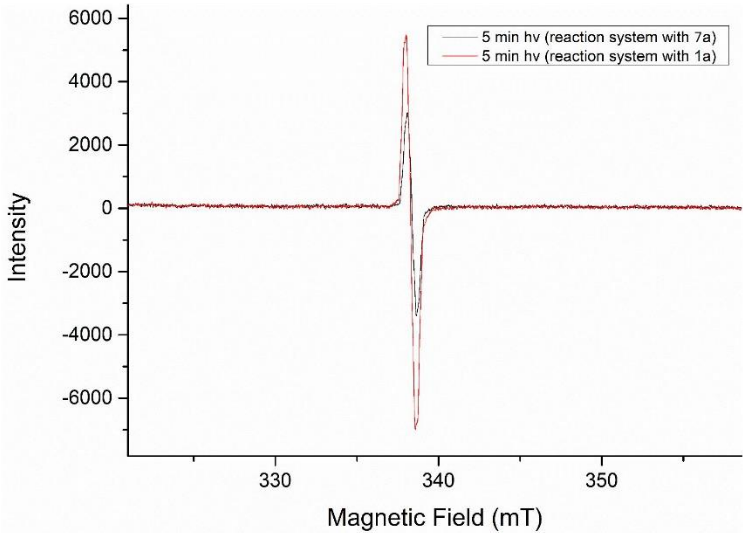

Figure S9. Overlaid *in-situ* EPR spectra of **1a** and **7a** with 4CzIPN and Cs_2CO_3 under 1 atm CO_2 in CH_3CN under 455 nm light irradiation for 5 min.

i) EPR of the mixture of **1a**, 4CzIPN and Cs_2CO_3 under N_2 without irradiation:

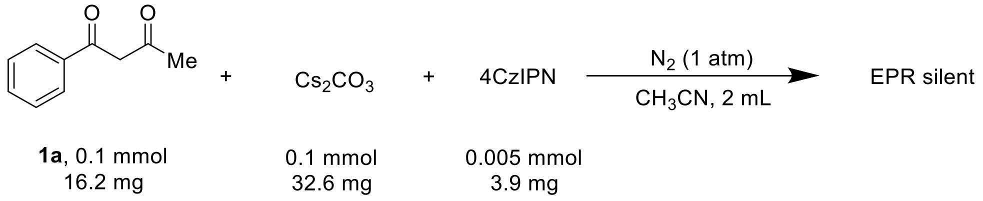

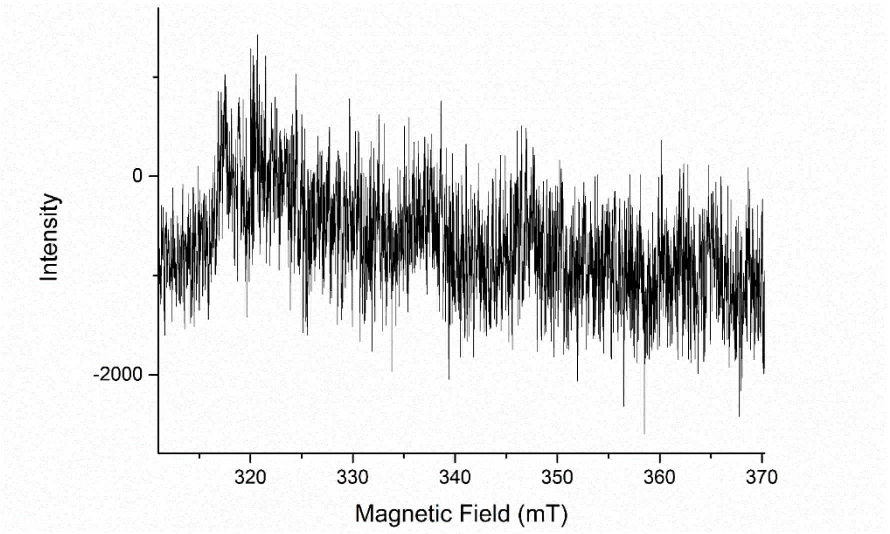

Figure S10. *In-situ* EPR spectrum of **1a**, 4CzIPN and Cs_2CO_3 under 1 atm N_2 in CH_3CN without irradiation.

j) EPR of the mixture of **1a**, 4CzIPN and Cs_2CO_3 under N_2 under 455 nm light irradiation:

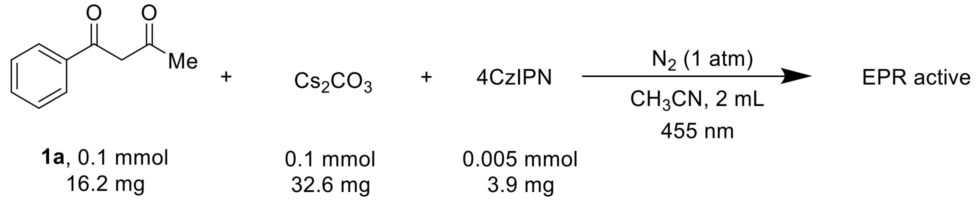

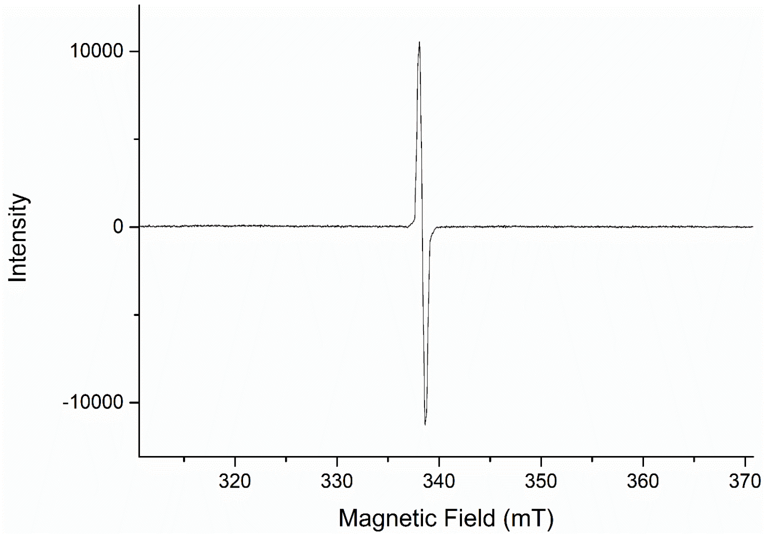

Figure S11. *In-situ* EPR spectra of **1a**, 4CzIPN and Cs_2CO_3 under 1 atm N_2 in CH_3CN under 455 nm light irradiation for 5 min.

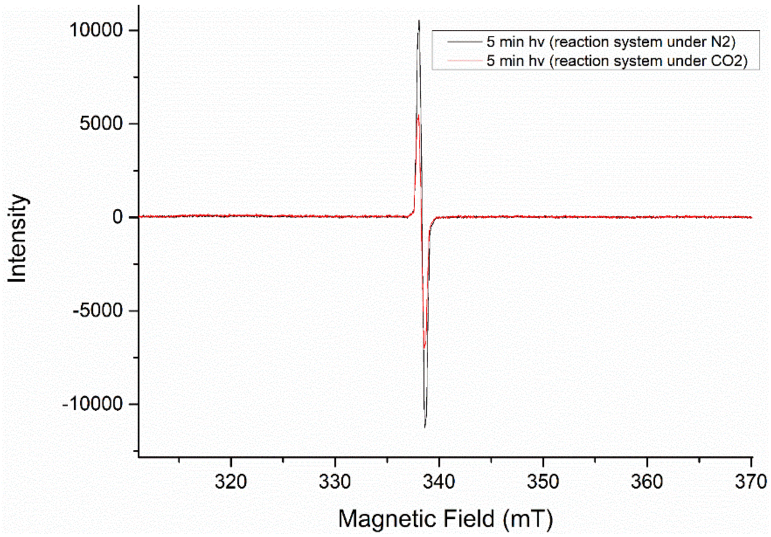

Figure S12. Overlaid *in-situ* EPR spectra of **1a**, 4CzIPN and Cs_2CO_3 under 1 atm N_2 and 1 atm CO_2 in CH_3CN under 455 nm light irradiation for 5 min.

k) EPR of the mixture of 4CzIPN and NEt_3 under N_2 without irradiation:

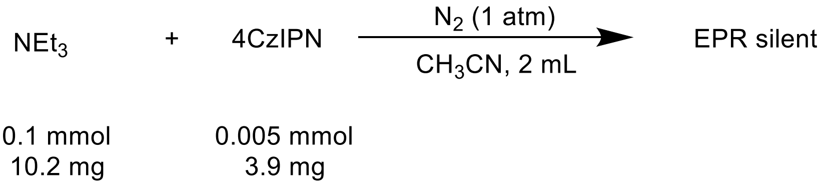

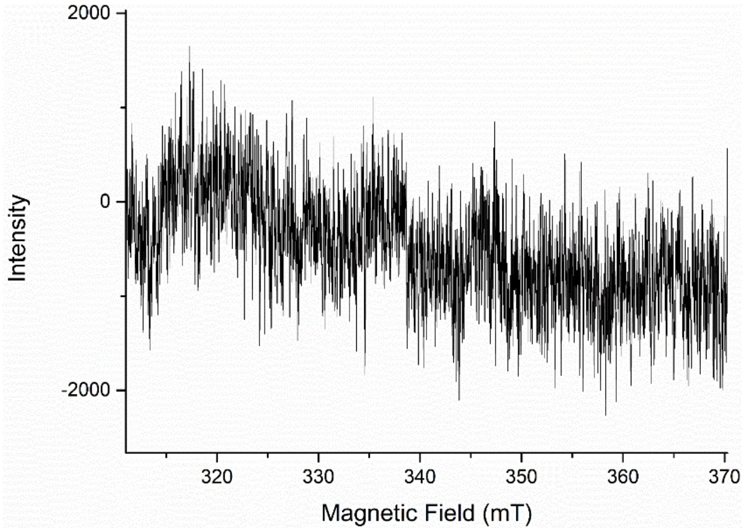

Figure S13. *In-situ* EPR spectrum of 4CzIPN and NEt_3 under 1 atm N_2 in CH_3CN without irradiation.

l) EPR of the mixture of 4CzIPN and NEt_3 under N_2 under 455 nm light irradiation:

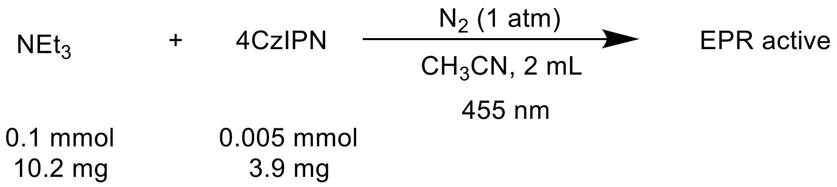

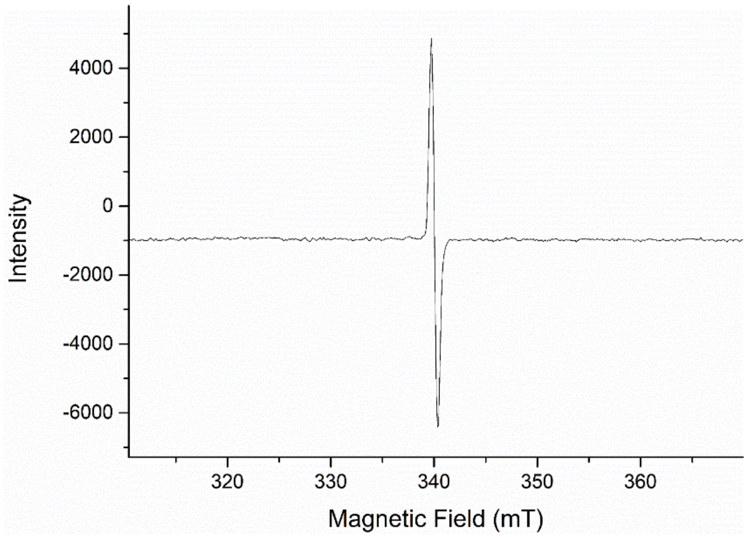

Figure S14. *In-situ* EPR spectrum of 4CzIPN and NEt_3 under 1 atm N_2 in CH_3CN under 455 nm light irradiation for 5 min. The chemical shift in the magnetic field is slightly shifted due to the different chemical environment.

V. Cyclic voltammetry

1a: 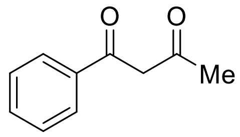

Index peak position

1 -1.3596

2 0.8207 (Fc/Fc$^+$)

3 0.7402 (Fc/Fc$^+$)

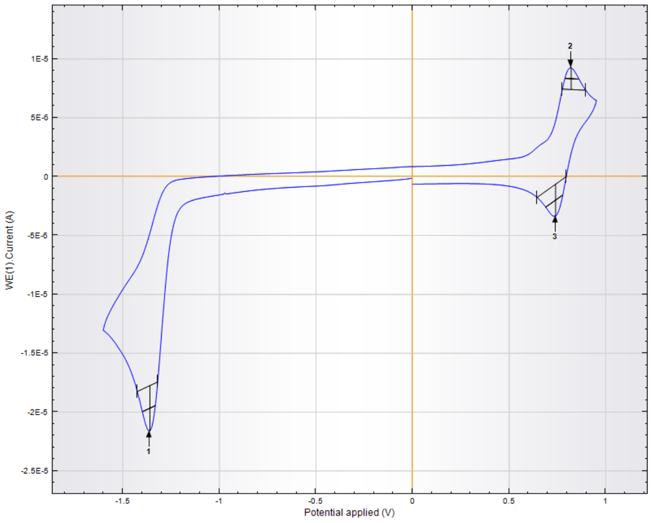

Figure S15. Cyclic voltammograms of **1a** and ferrocene internal reference in DMF. Reversible ferrocene/ferrocenium oxidation peak is seen at $E_{1/2}$ = 0.7805 V.

1k: 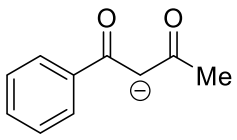

Index peak position

1 1.2186

2 1.3495 (Fc/Fc$^+$)

3 1.2790 (Fc/Fc$^+$)

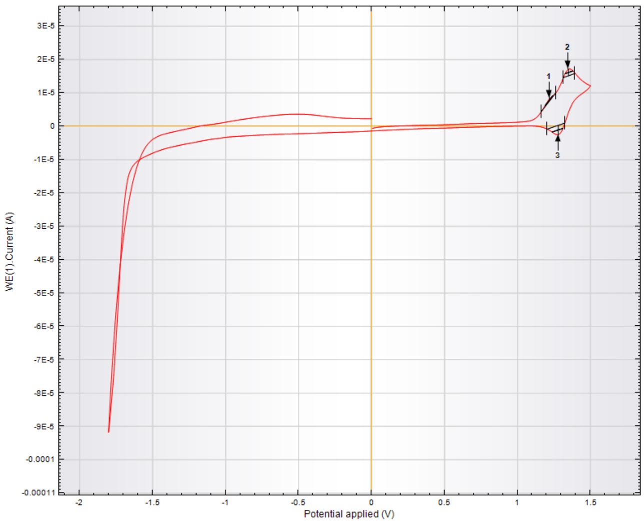

Figure S16. Cyclic voltammograms of **1k** and ferrocene internal reference in DMF. Reversible ferrocene/ferrocenium oxidation peak is seen at $E_{1/2}$ = 1.3143 V.

VI. Stern-Volmer luminescence quenching experiments

Luminescence spectra of 4CzIPN (2.10 μM) were collected in dry and degassed DMF with **1a** varying from 0.00 to 5.93 mM in concentration and 2 mg Cs_2CO_3. Emission spectra of these solutions are shown in **Figure S17**. The photoluminescence quenching data were analyzed (**Figure S18**) using the Stern-Volmer equation (1):

$$I_0/I = 1 + K_{SV}[Q] \quad (1)$$

where I_0 and I are the steady-state emission intensities of 4CzIPN in the absence and in the presence of a quencher **1a** and Cs_2CO_3, respectively. [Q] is the molar concentration of the quencher. K_{SV} is the Stern-Volmer constant.

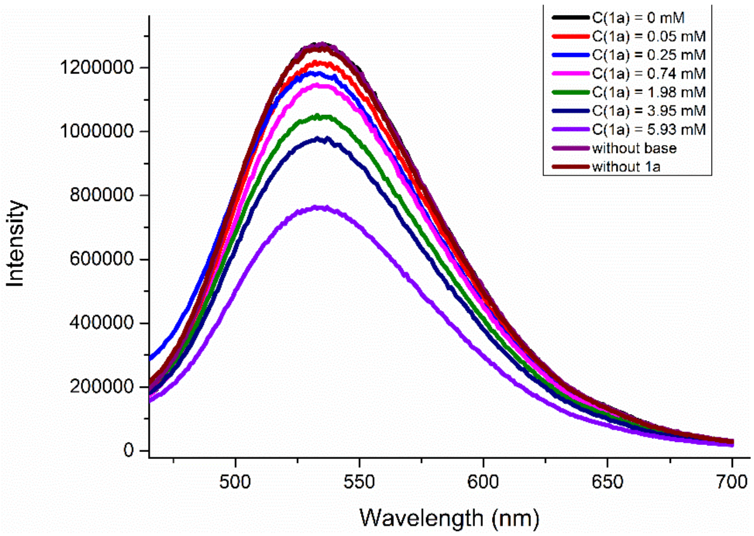

Figure S17. Emission spectra of 4CzIPN in the presence of **1a** and Cs_2CO_3.

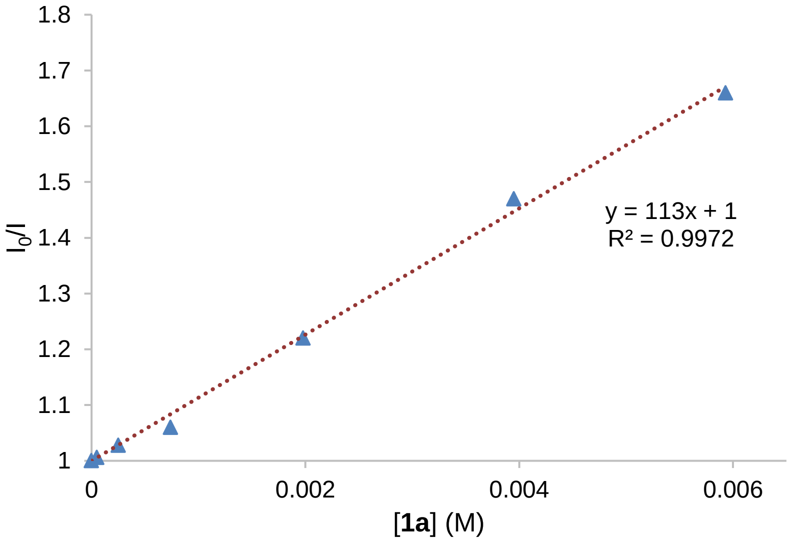

Figure S18. Stern-Volmer quenching of 4CzIPN with Cs_2CO_3 and varying [**1a**].

VII. Light "on-off" experiments

A mixture of 1-phenyl-1,3-butanedione **1a** (0.1 mmol), 4CzIPN (0.005 mmol), Cs_2CO_3 (0.15 mmol) was added to an oven-dried 5 mL snap vial equipped with a magnetic stirring bar. The capped vial was evacuated and back filled with CO_2 for three times and dry DMF (2 mL) was added to the vial by syringe. The solution was then bubbled with CO_2 for 5 min. Then, the cap was sealed with parafilm. The reaction mixture was stirred at 25 °C. Parallel reactions were prepared and the conversions of **1a** at different times with 455 nm light irradiation or without light irradiation were monitored.

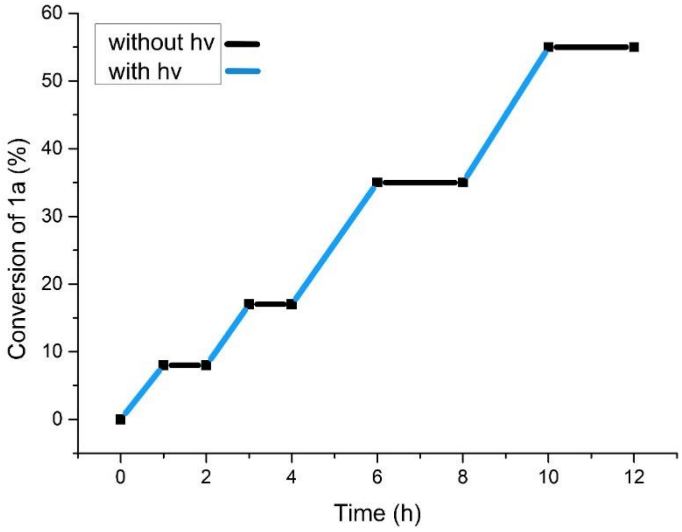

Figure S19. Conversion of **1a** at different times with 455 nm light irradiation or without light irradiation. Each data point represents a separately measured reaction.

VIII. *In-situ* NMR investigations

In-situ NMR spectra of pure 1-phenyl-1,3-butanedione (**1a**), mixture of **1a** and CO_2, pure 1-(4-fluorophenyl)-1,3-butanedione (**6a**), mixture of **6a** and CO_2 were recorded (**Figures S20-S31**), which revealed that CO_2 can react with the OHs group of enols.

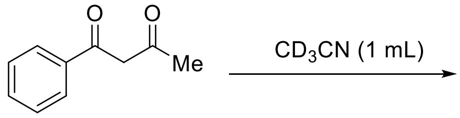

1a, 0.1 mmol

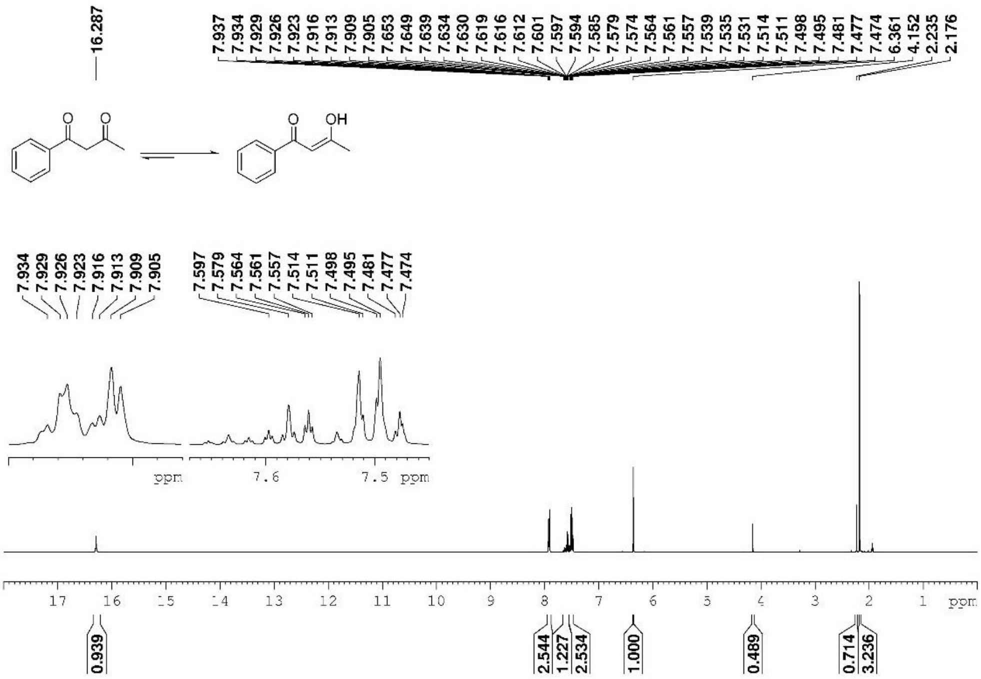

Figure S20. ^{1}H NMR spectrum (400 MHz, CD_3CN).

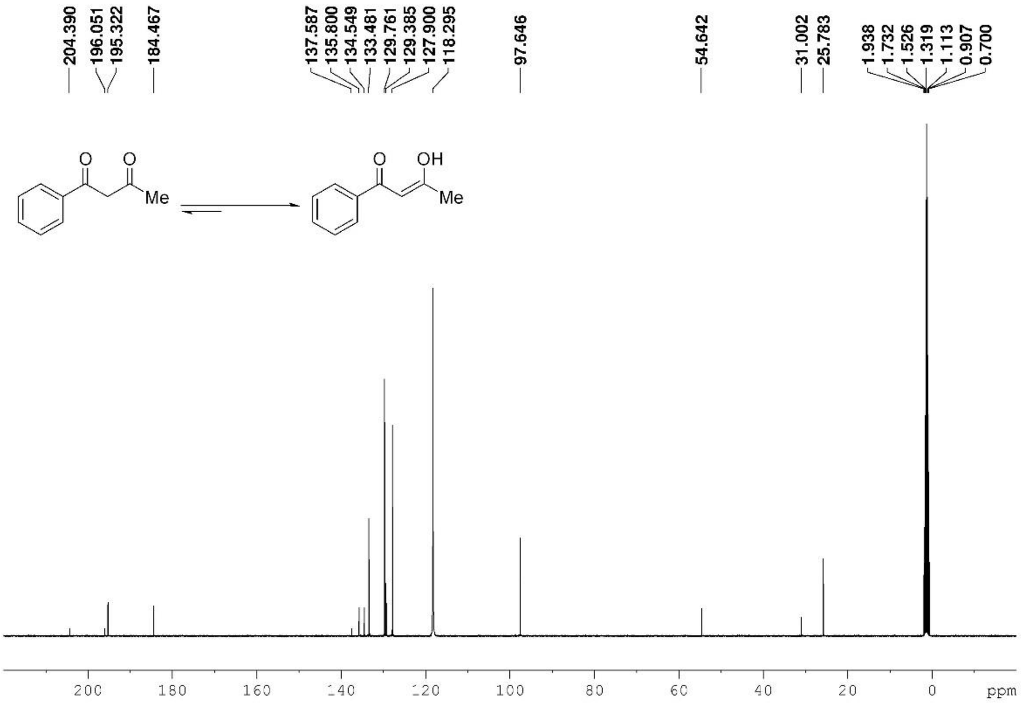

Figure S21. $^{13}C\{^1H\}$ NMR spectrum (101 MHz, CD_3CN).

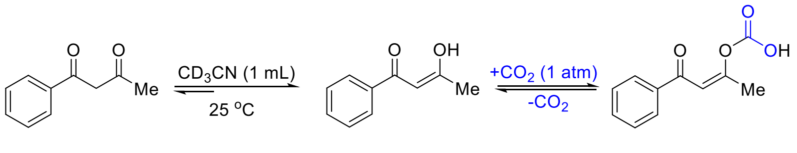

1a, 0.1 mmol

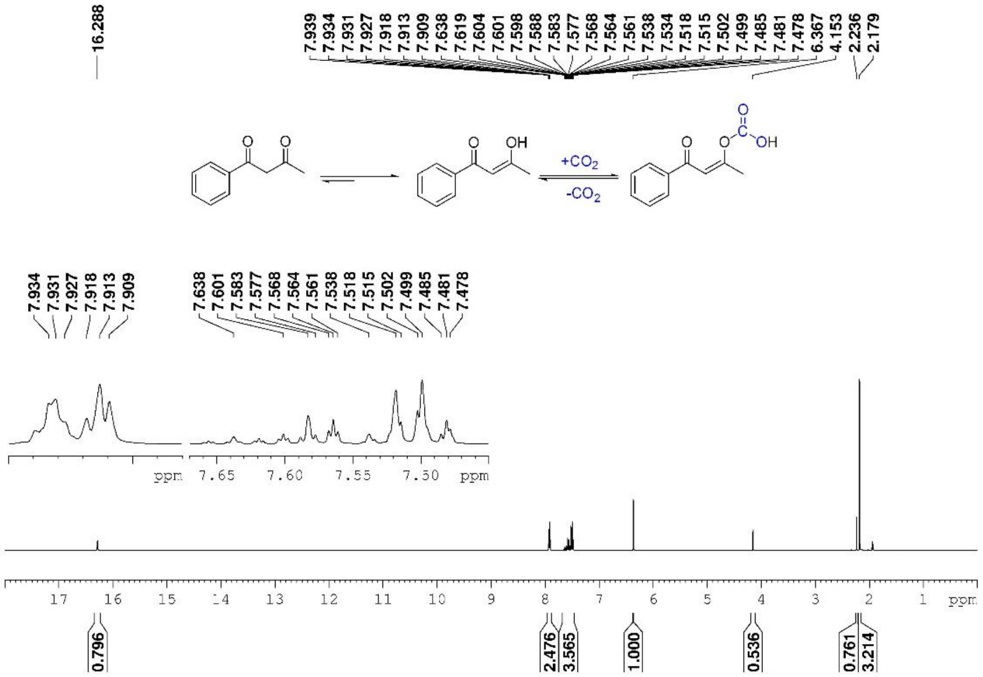

Figure S22. ^1H NMR spectrum (400 MHz, CD_3CN).

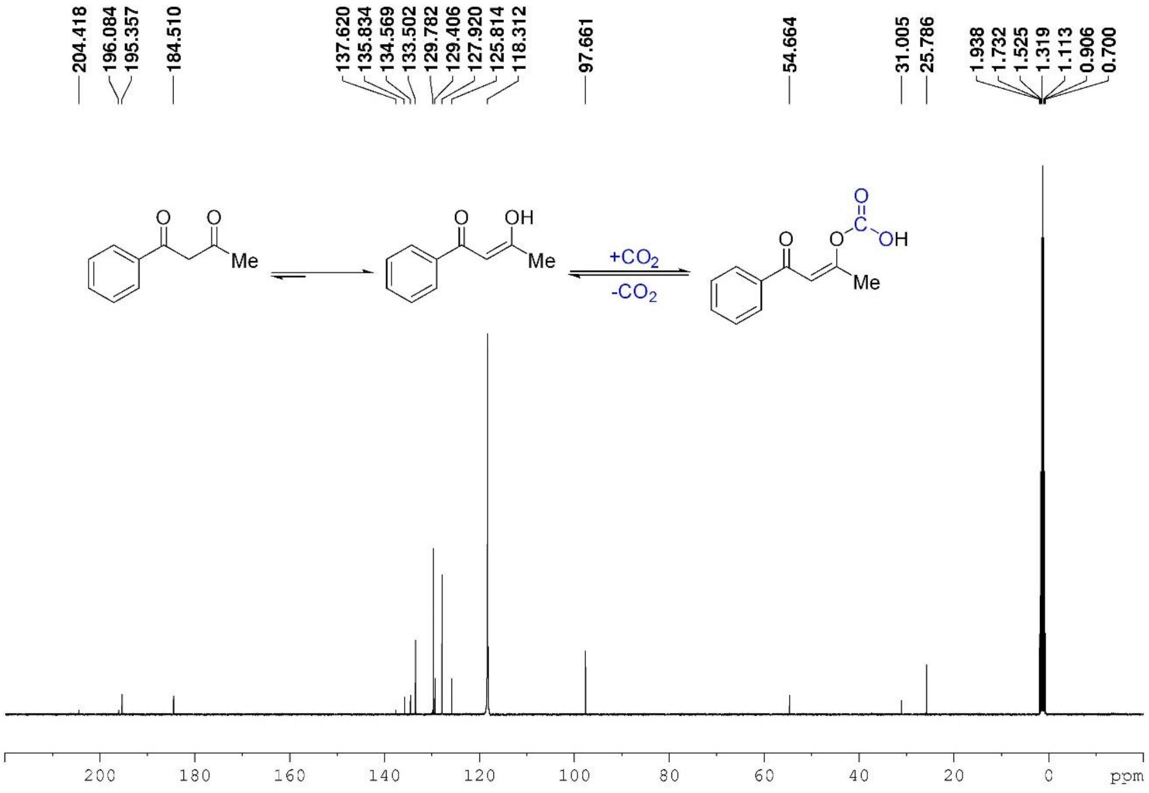

Figure S23. $^{13}C\{^{1}H\}$ NMR spectrum (101 MHz, CD_3CN).

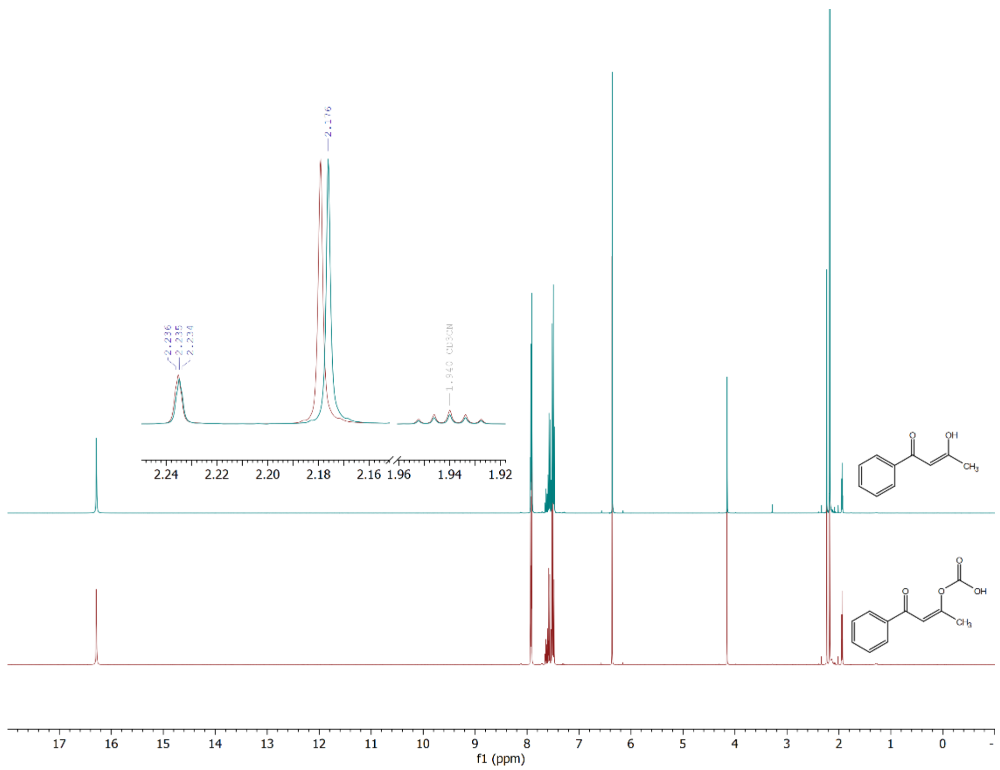

Figure S24. Overlaid ^{1}H NMR spectra (400 MHz, CD_3CN) of **1a** and **1d**, with a zoomed-in cutout showing the region with significant difference (some ranges were removed to better demonstrate the small chemical shift changes).

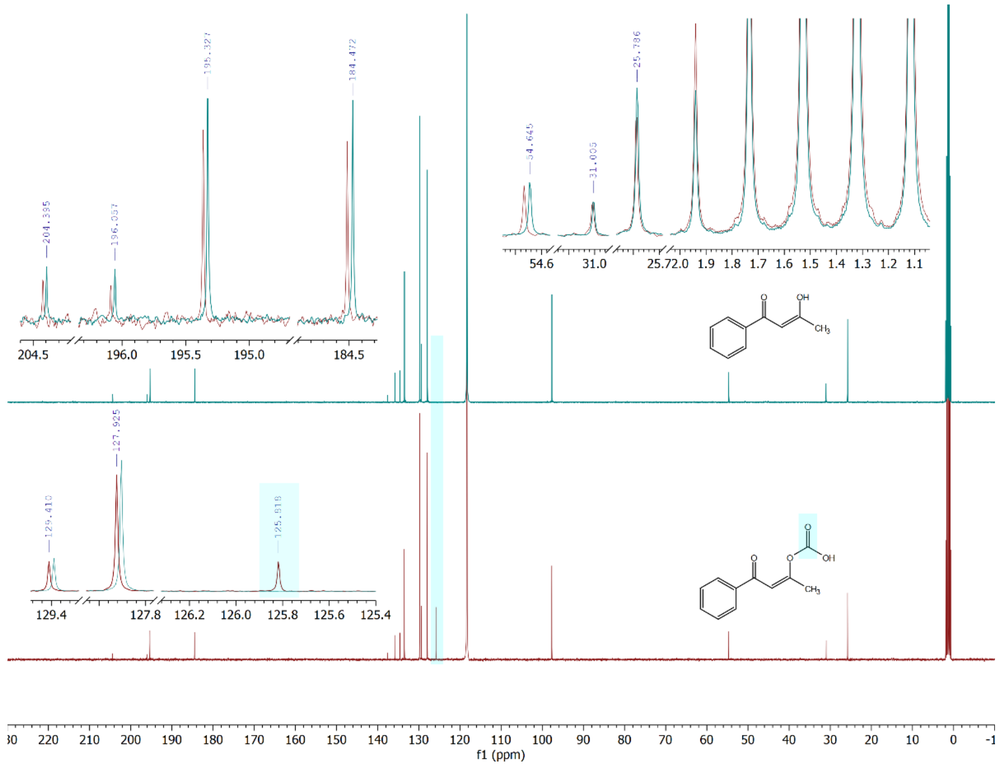

Figure S25. Overlaid $^{13}C\{^1H\}$ NMR spectra (101 MHz, CD_3CN) of **1a** and **1d**, with zoomed-in cutouts showing the regions with significant differences (some ranges were removed to better demonstrate the small chemical shift changes). The new peak corresponding to the carbonic acid carbon is highlighted.

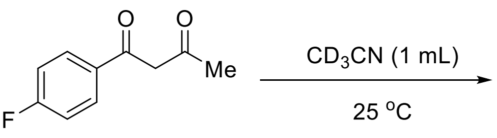

1a, 0.1 mmol

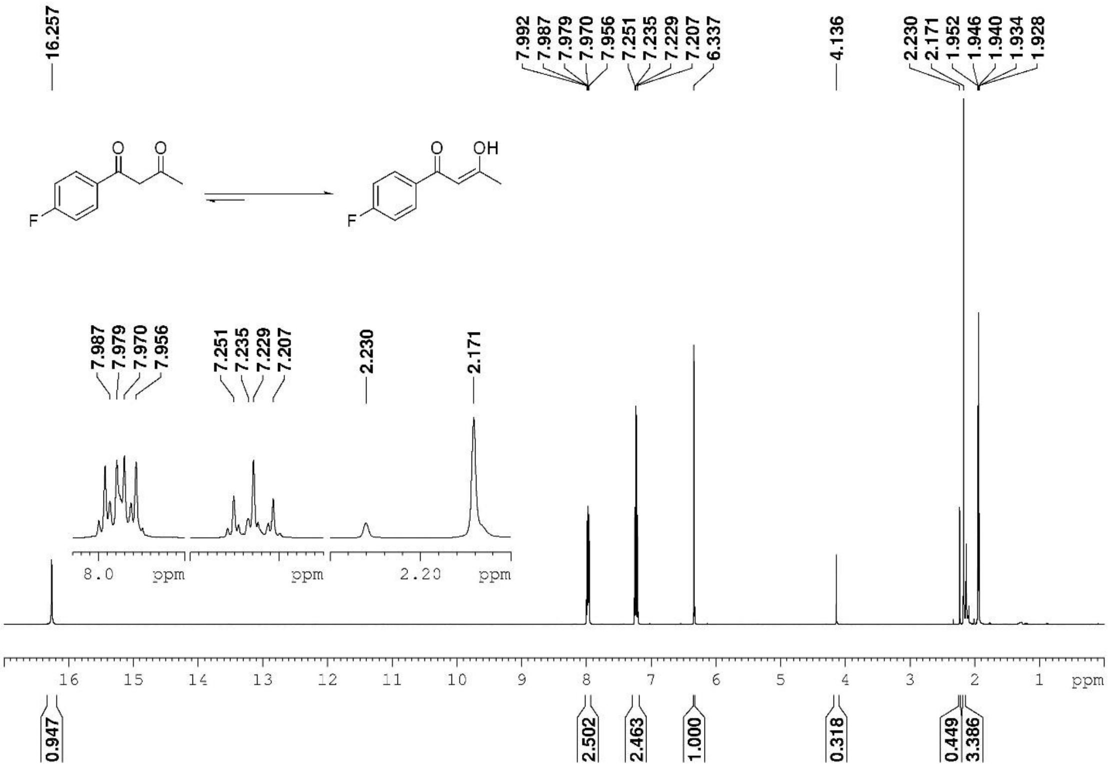

Figure S26. ^{1}H NMR spectrum (400 MHz, CD_3CN).

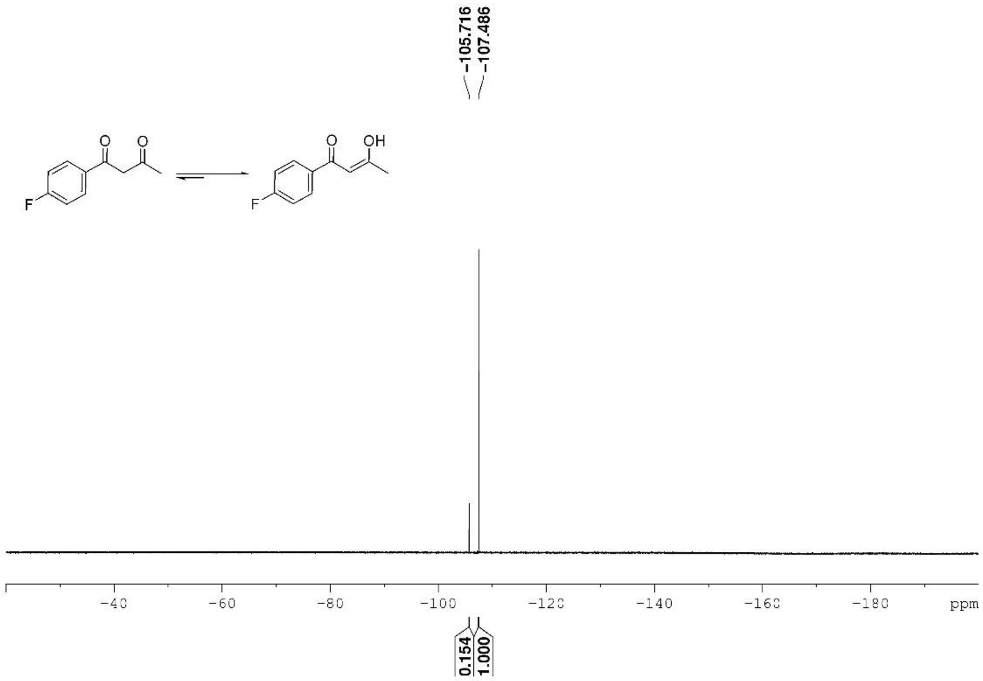

Figure S27. $^{19}F\{^{1}H\}$ NMR spectrum (376 MHz, CD_3CN).

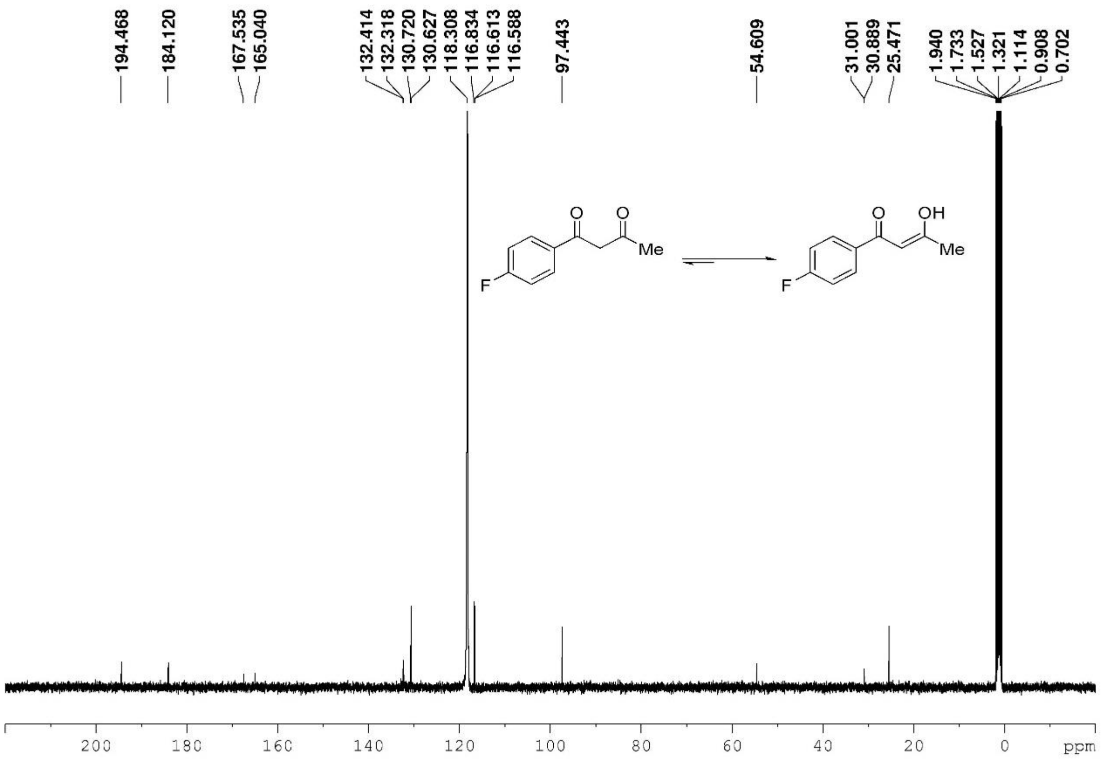

Figure S28. $^{13}C\{^{1}H\}$ NMR spectrum (101 MHz, CD_3CN).

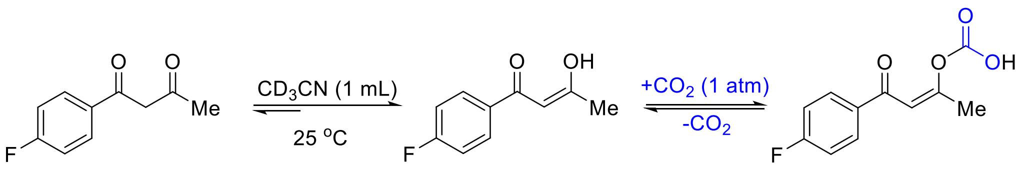

1a, 0.1 mmol

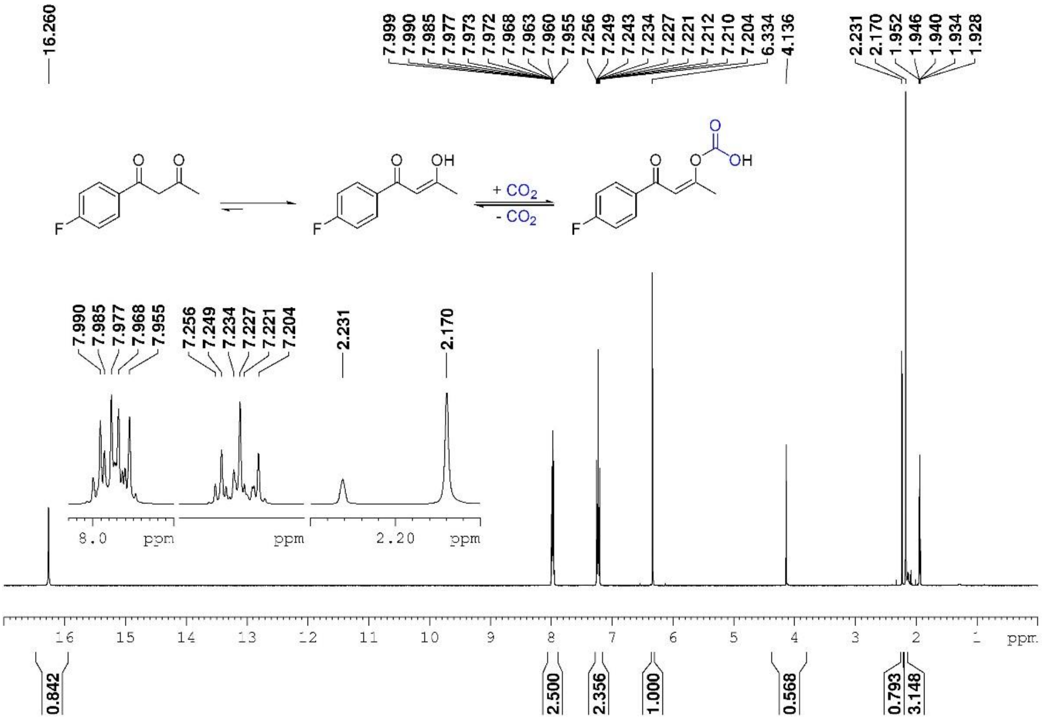

Figure S29. ^{1}H NMR spectrum (400 MHz, CD_3CN).

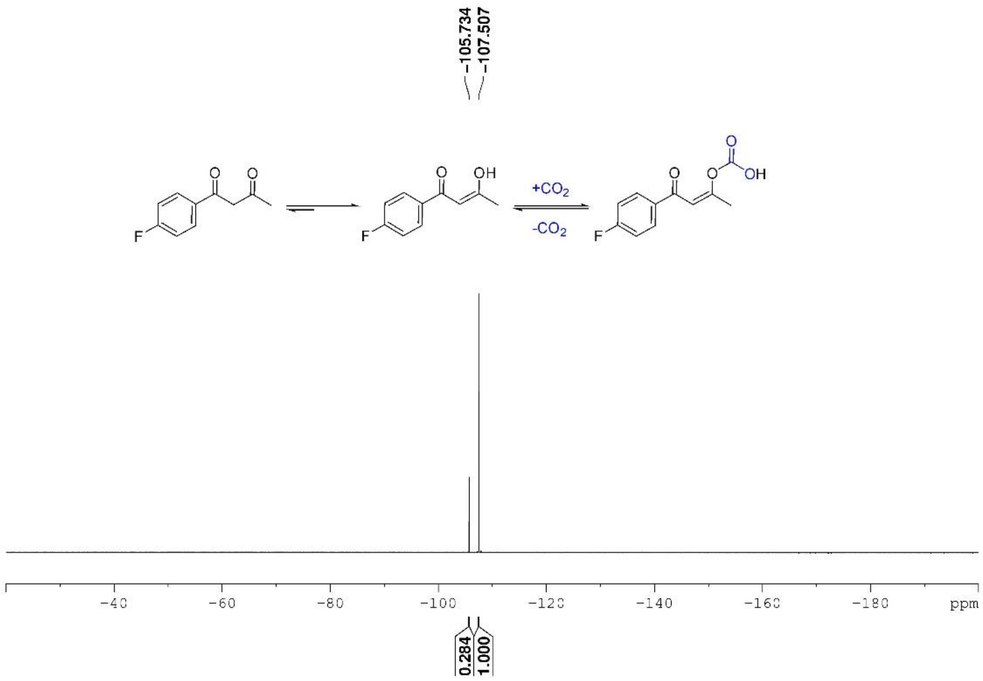

Figure S30. $^{19}F\{^{1}H\}$ NMR spectrum (376 MHz, CD_3CN).

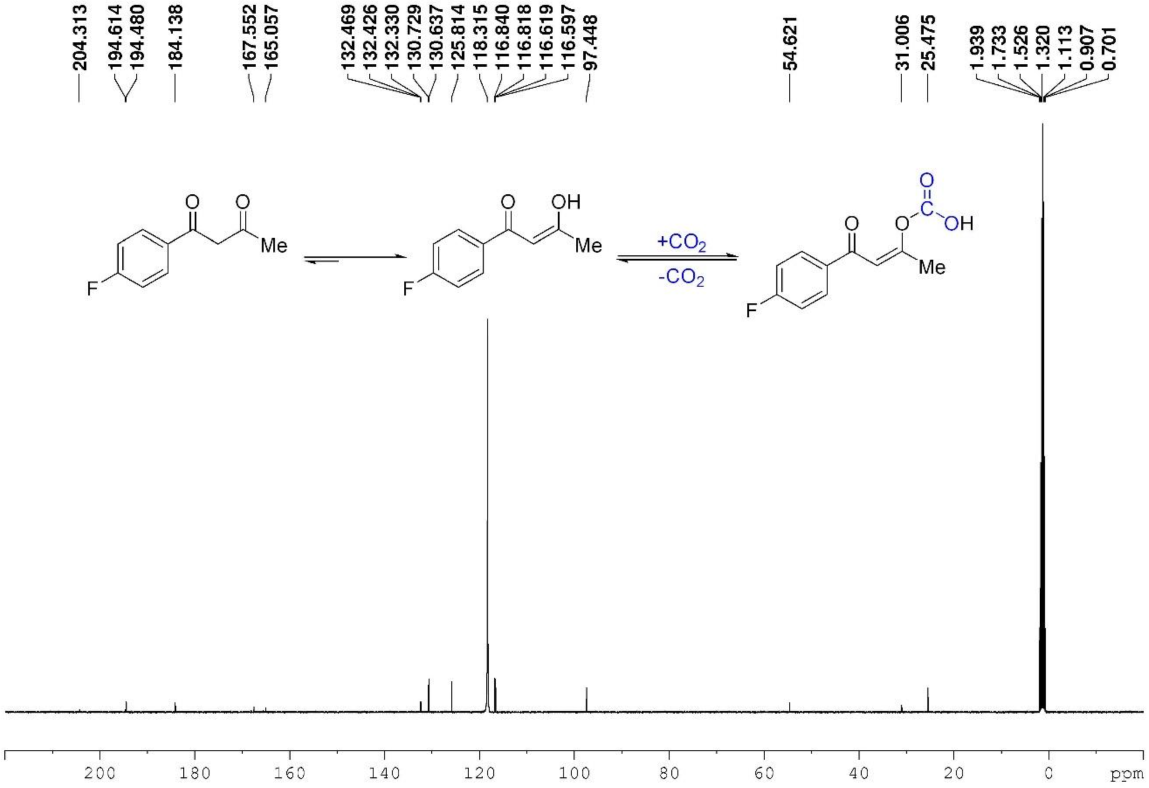

Figure S31. $^{13}C\{^{1}H\}$ NMR spectrum (101 MHz, CD_3CN).

In-situ NMR spectrum of the mixture of **1a** and Cs_2CO_2, was recorded (**Figure S32**), which revealed that **1a** can be deprotonated by Cs_2CO_3.

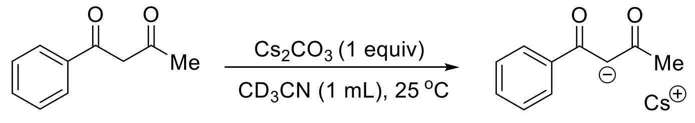

1a, 0.1 mmol

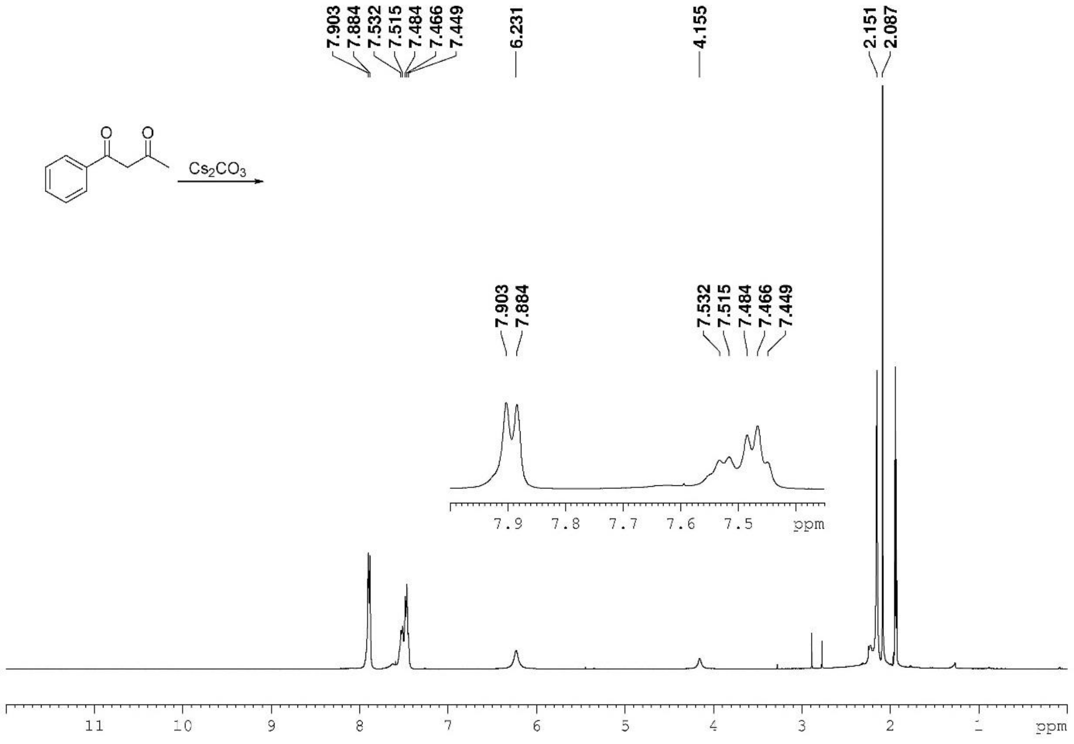

Figure S32. ^{1}H NMR spectrum (400 MHz, CD_3CN).

To test the reactivity of 1-phenyl-1,3-butanedione (**1a**) with 4CzIPN, two identical reaction mixture were prepared. Then one was kept in the dark and another one irradiated with 455 nm LEDs. They were subsequently studied by ^{1}H NMR spectroscopy after 5 h, which revealed no sign of reaction (**Figures S33-S36**).

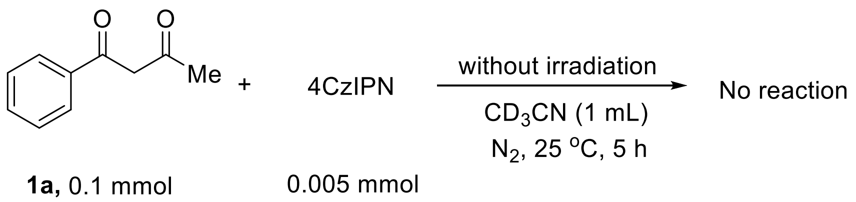

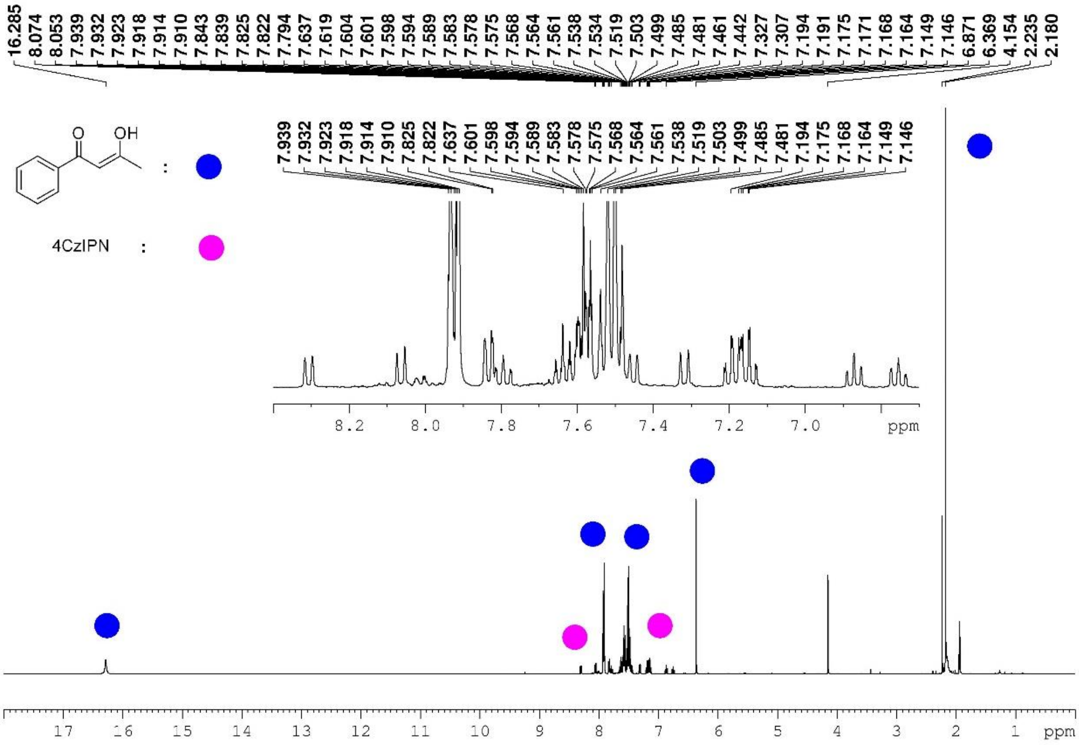

Figure S33. ^{1}H NMR spectrum at 0 min (400 MHz, CD_3CN).

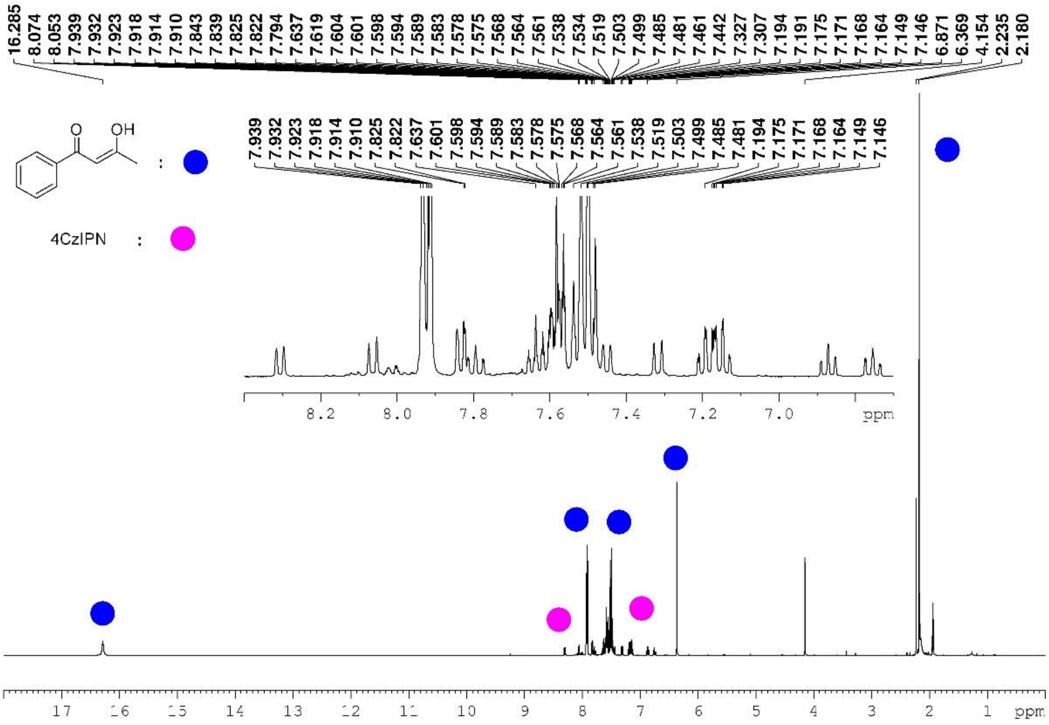

Figure S34. ^{1}H NMR spectrum after 5 h in the dark (400 MHz, CD_3CN).

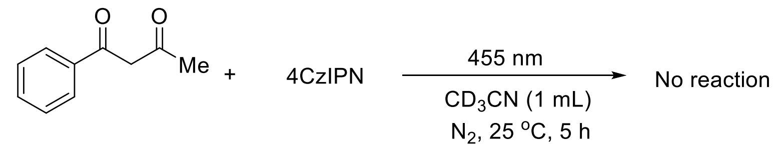

1a, 0.1 mmol 0.005 mmol

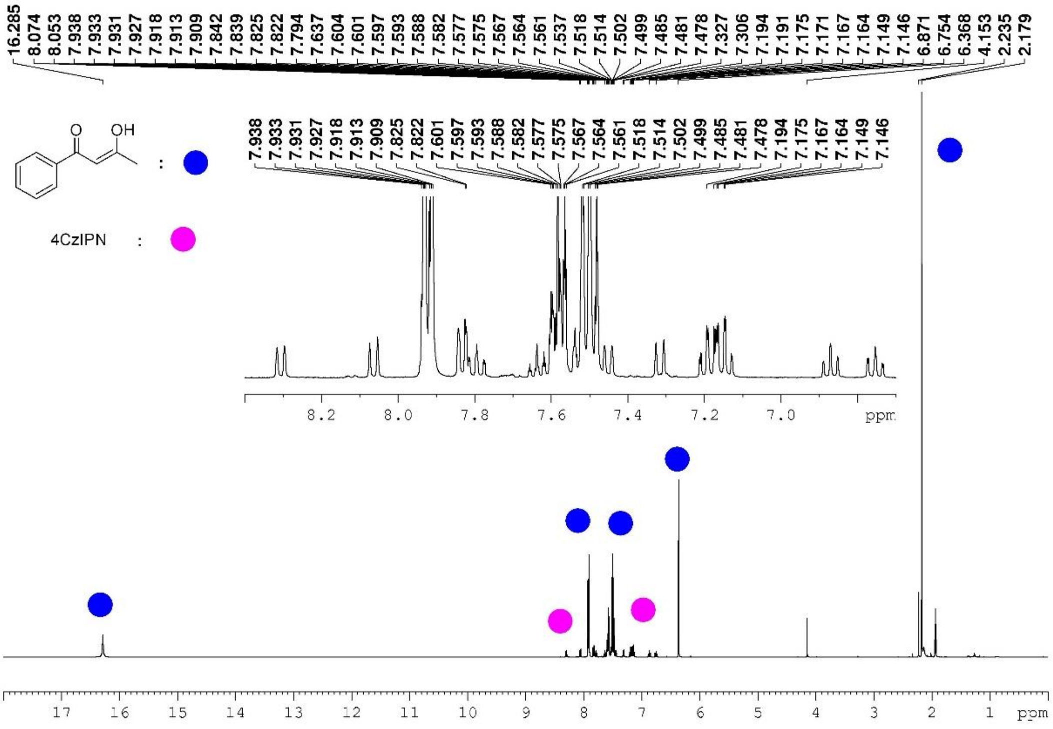

Figure S35. ^{1}H NMR spectrum at 0 min (400 MHz, CD_3CN).

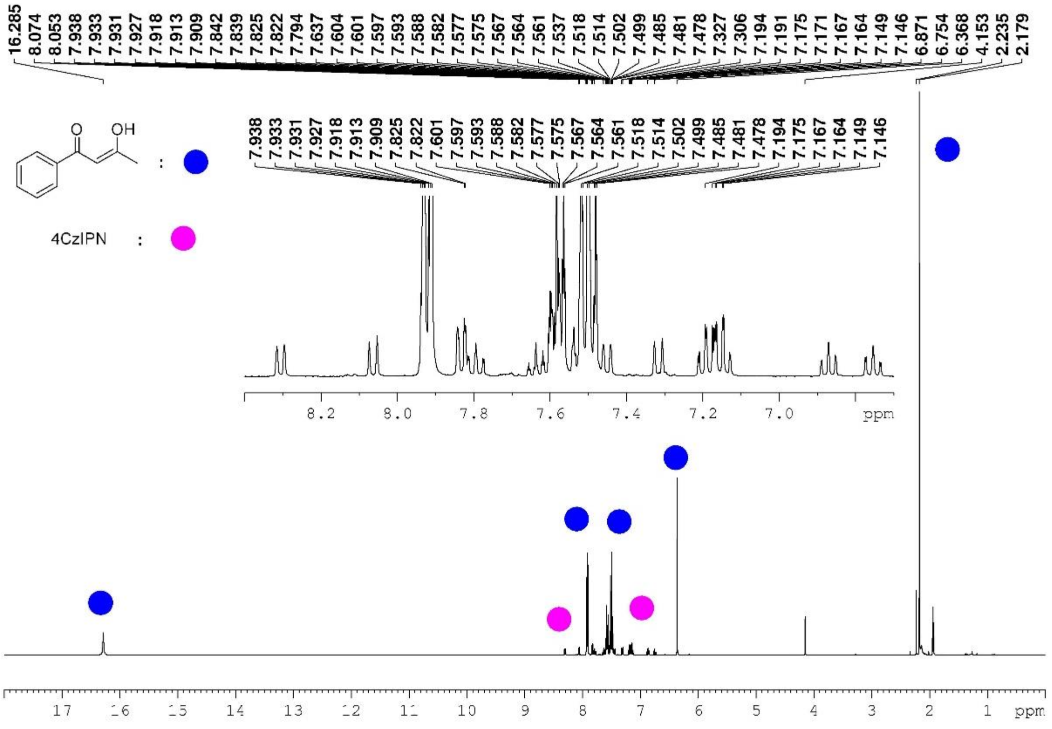

Figure S36. ^{1}H NMR spectrum after 5 h under irradiation (400 MHz, CD_3CN).

To test the reactivity of 1-phenyl-1,3-butanedione (**1a**) with CO_2 and 4CzIPN, two identical reaction mixture were prepared. Then one was kept in the dark and another one irradiated with 455 nm LEDs. They were subsequently studied by ^{1}H NMR spectroscopy after 5 h, which revealed no sign of reaction (**Figures S37-S40**).

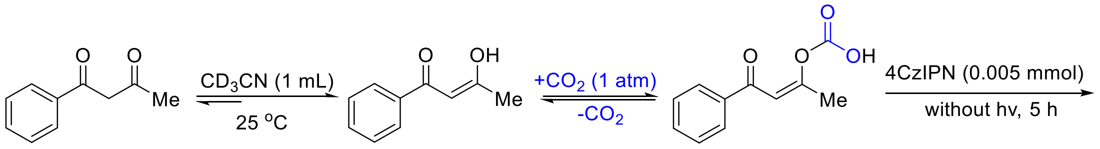

1a, 0.1 mmol

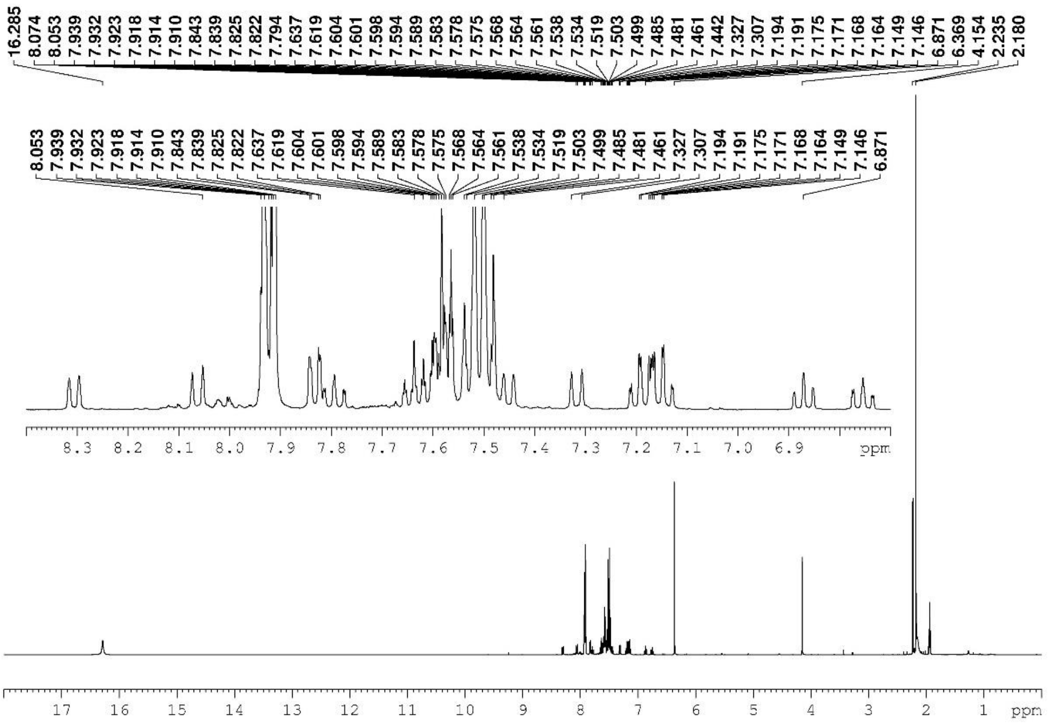

Figure S37. ^{1}H NMR spectrum at 0 min (400 MHz, CD_3CN).

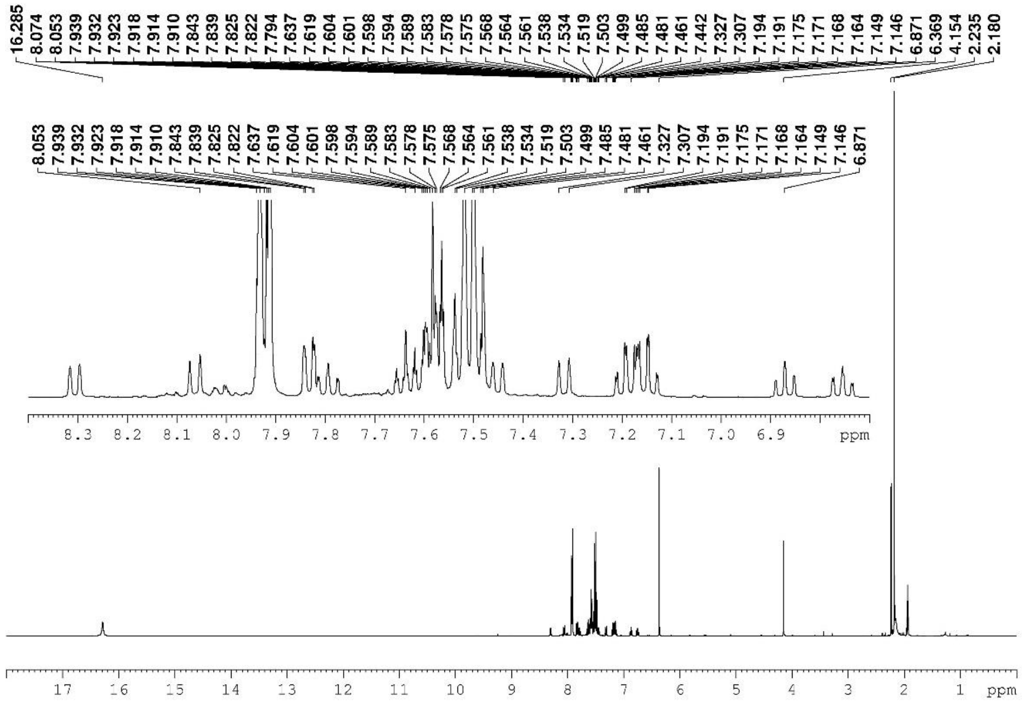

Figure S38. ^{1}H NMR spectrum after 5 h in the dark (400 MHz, CD_3CN).

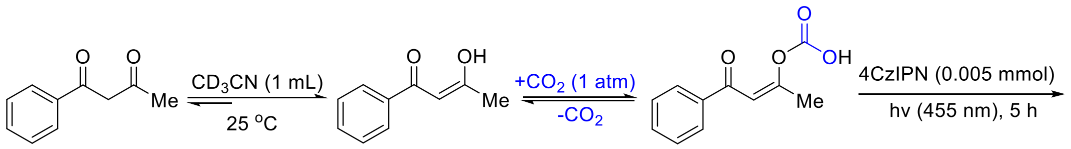

1a, 0.1 mmol

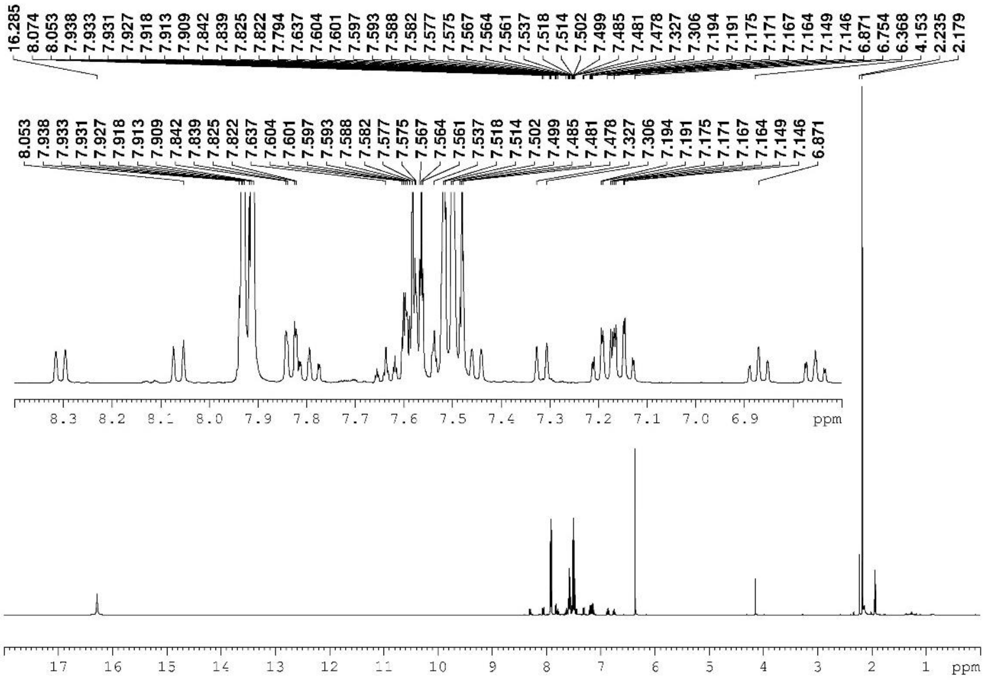

Figure S39. ^{1}H NMR spectrum at 0 min (400 MHz, CD_3CN).

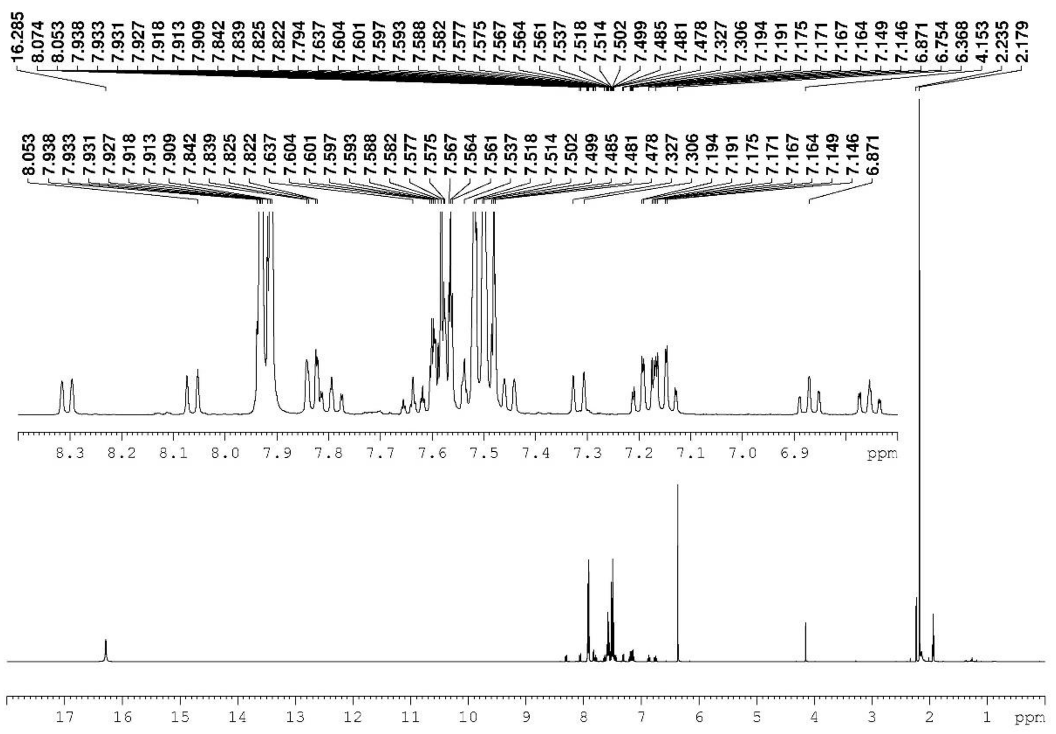

Figure S40. ^{1}H NMR spectrum after 5 h under irradiation (400 MHz, CD_3CN).

To test the reactivity of 1-phenyl-1,3-butanedione (**1a**) with Cs_2CO_3 and 4CzIPN, two identical reaction mixture were prepared. Then one was kept in the dark and another one irradiated with 455 nm LEDs. They were subsequently studied by ^{1}H NMR spectroscopy after 5 h, which revealed no sign of reaction without light irradiation (**Figures S41, S42**). However, from the ^{1}H NMR spectra recorded after 5 h irradiation, new peaks were observed (**Figures S43, S44**), indicating a reaction occurred in this system.

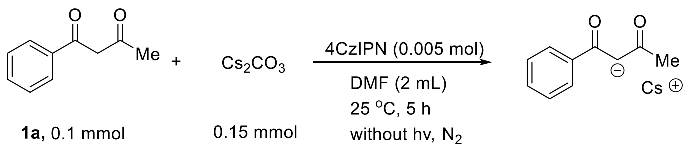

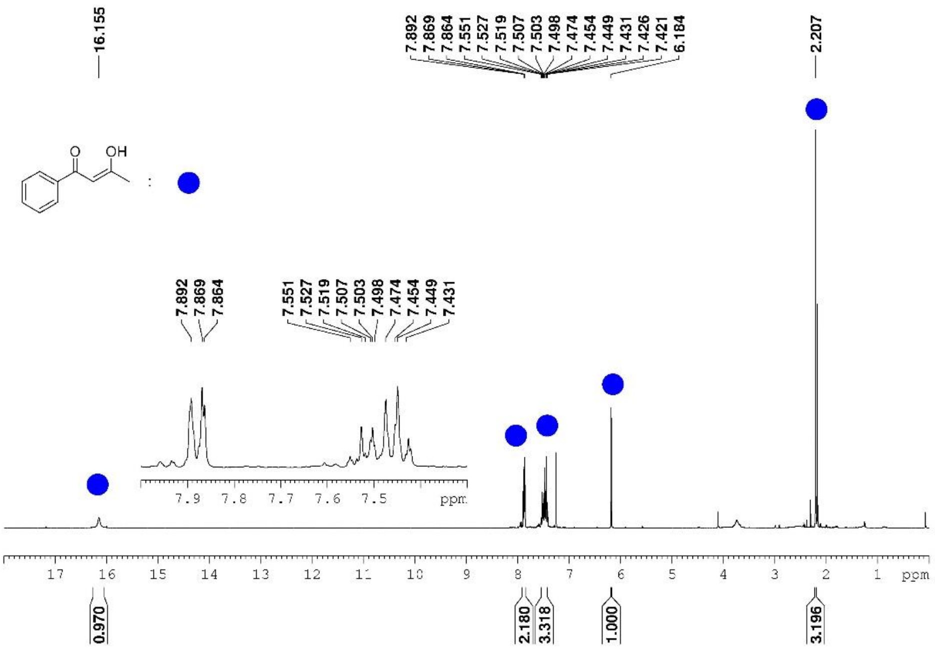

Figure S41. ^{1}H NMR spectrum at 0 min (300 MHz, $CDCl_3$).

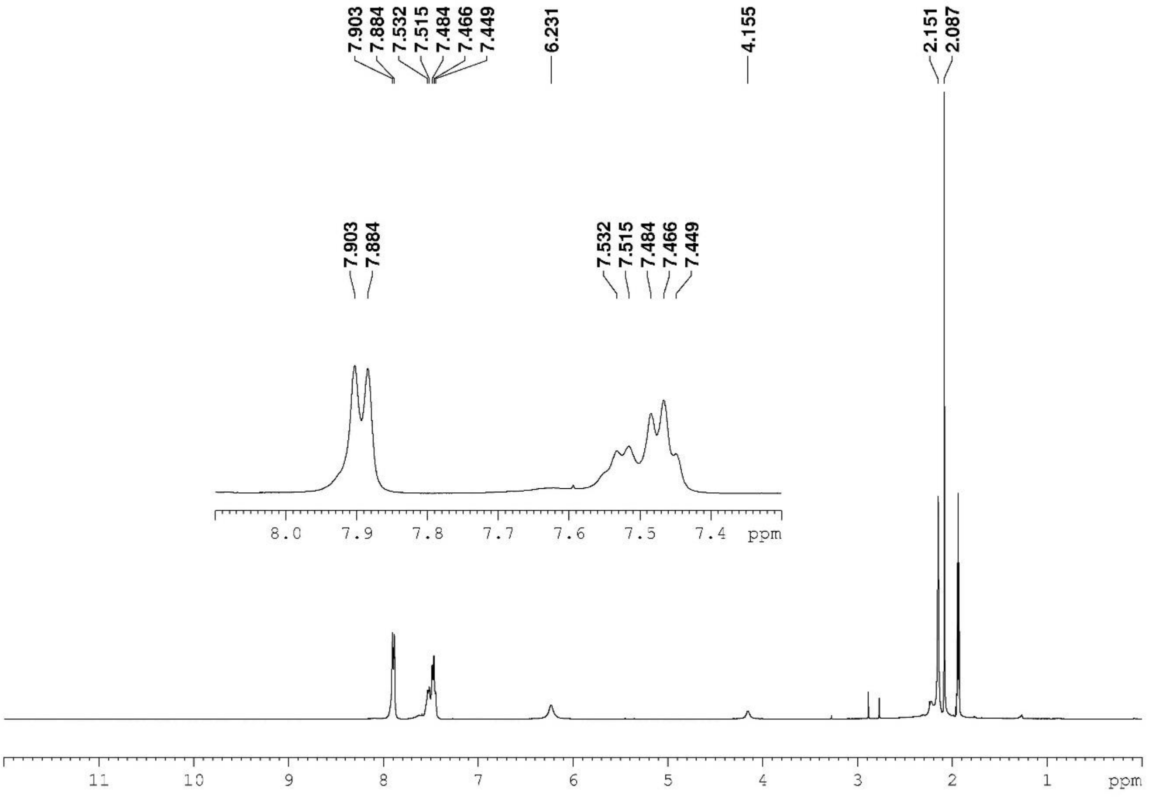

Figure S42. ^{1}H NMR spectrum after 5 h in the dark (300 MHz, $CDCl_3$).

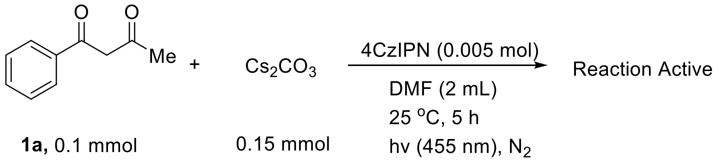

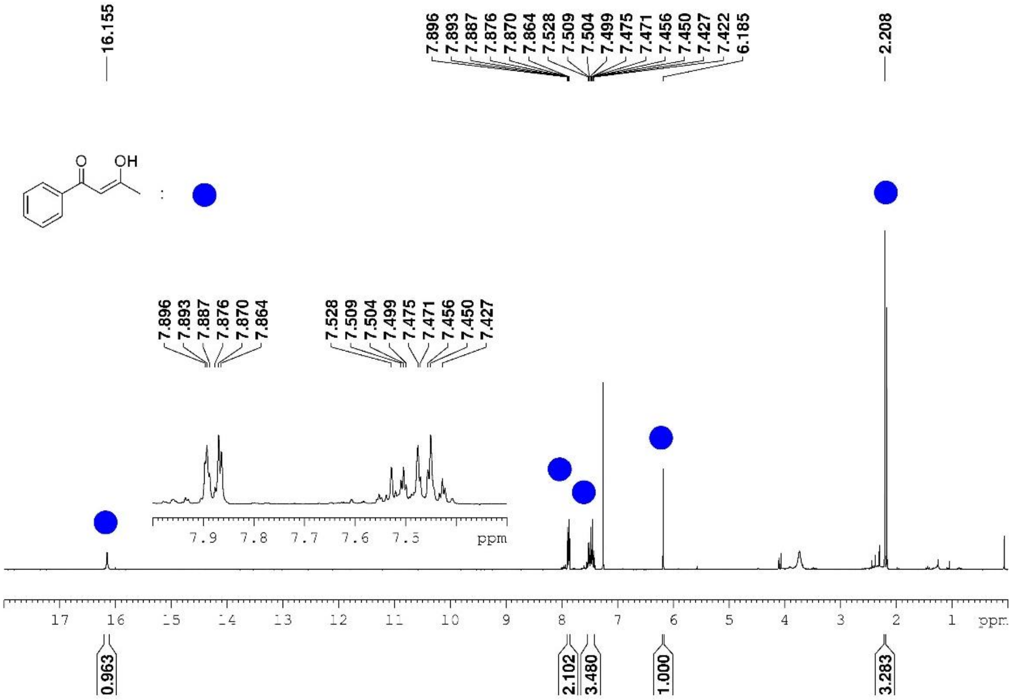

Figure S43. ^{1}H NMR spectrum at 0 min (300 MHz, $CDCl_3$).

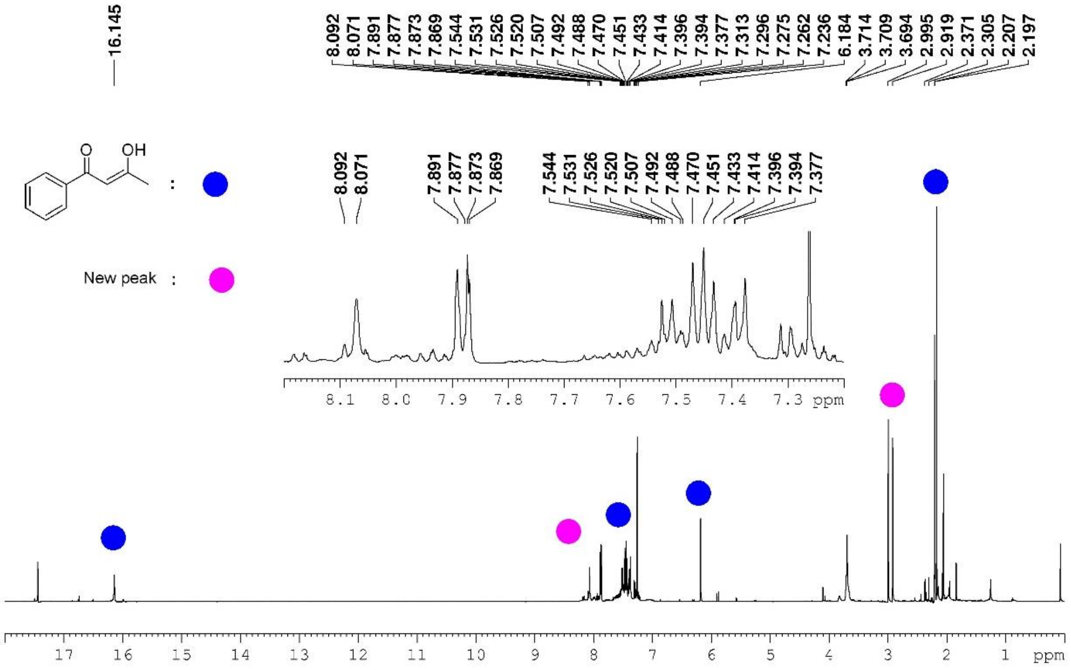

Figure S44. ^{1}H NMR spectrum after 5 h under irradiation (300 MHz, $CDCl_3$).

For the stander reaction, two identical reaction mixture were prepared. Then one was kept in the dark and another one irradiated with 455 nm LEDs. They were subsequently studied by ^{1}H NMR spectroscopy after 5 h, which revealed no sign of a reaction without light irradiation (**Figures S45, S46**). As we expected, from the ^{1}H NMR spectra recorded after 5 h irradiation, we could observe the signal for the products **1b** and **1c** (**Figures S47, S48**).

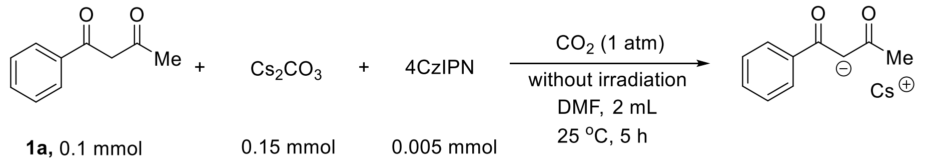

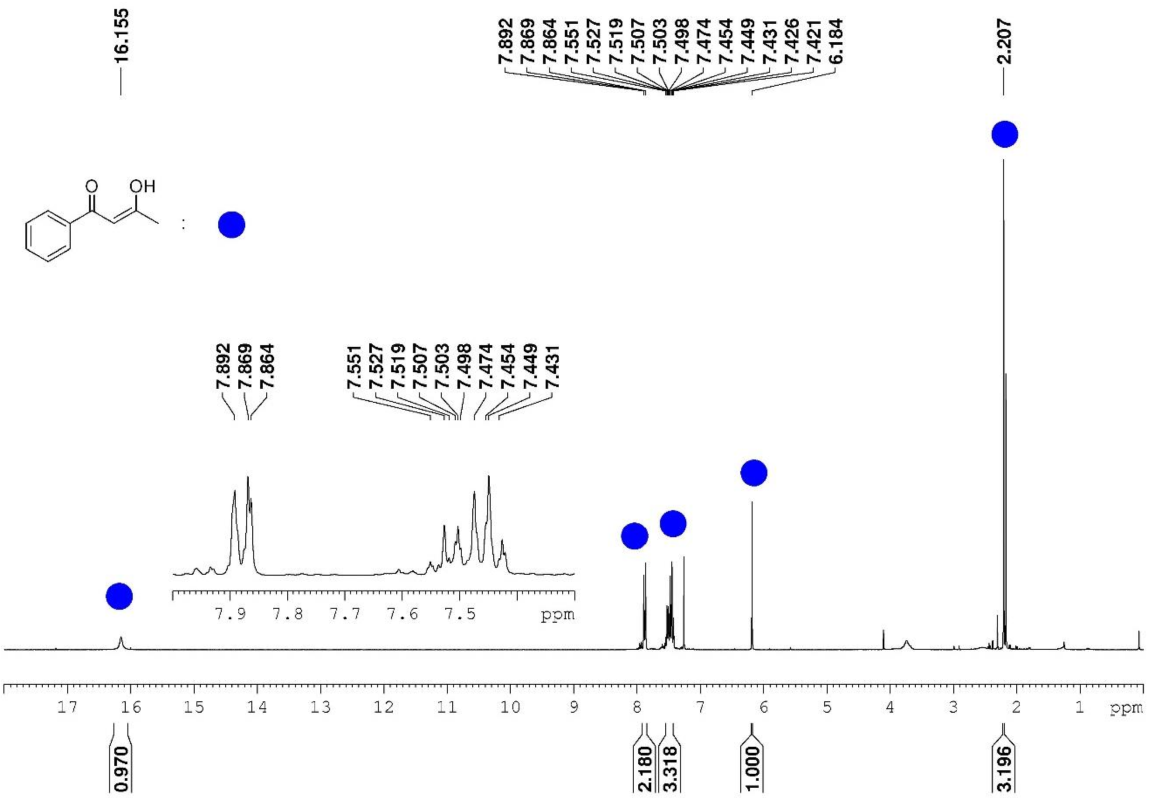

Figure S45. ^{1}H NMR spectrum at 0 min (300 MHz, $CDCl_3$).

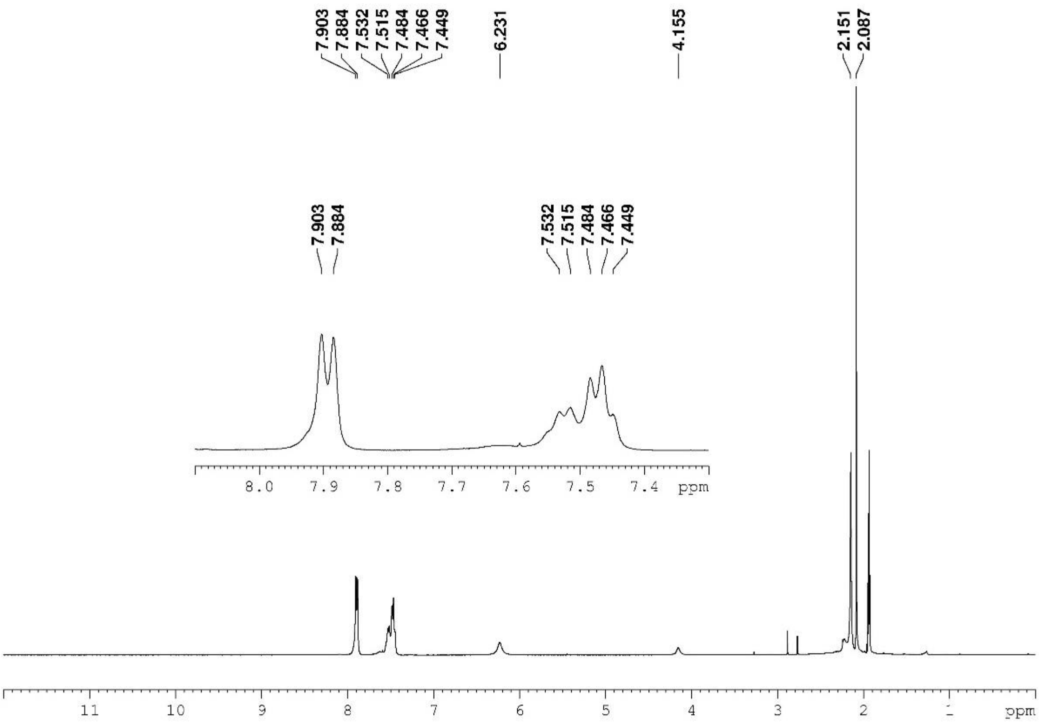

Figure S46. ^{1}H NMR spectrum after 5 h in the dark (300 MHz, $CDCl_3$).

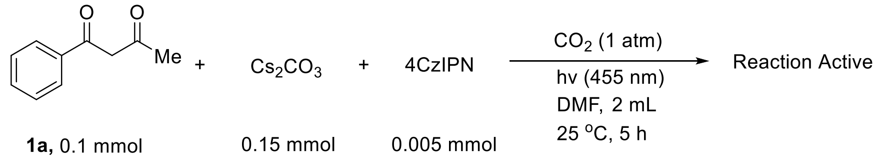

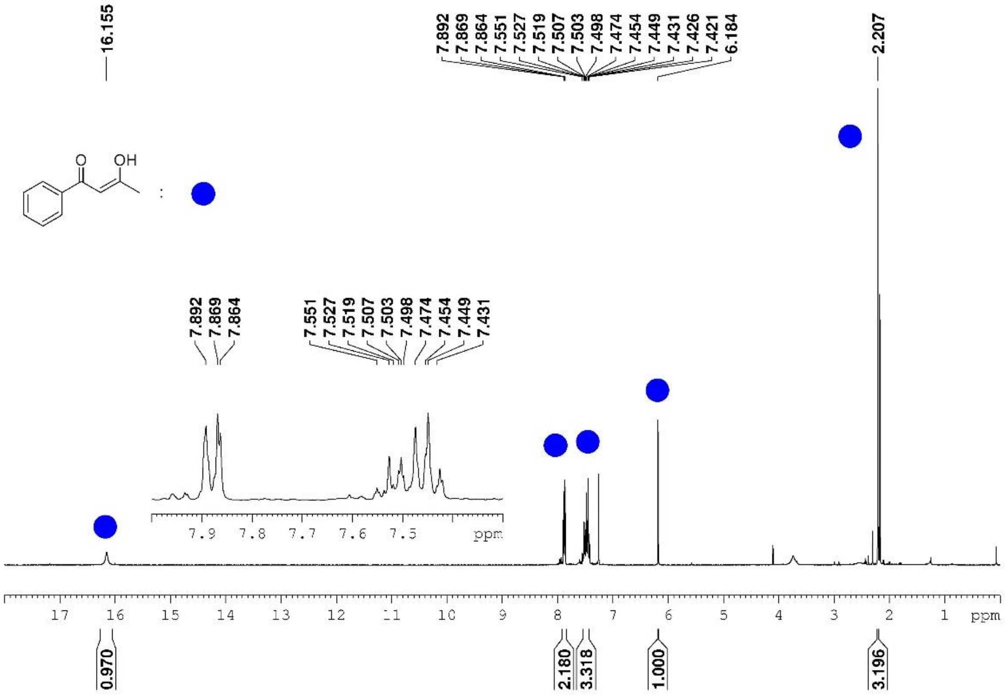

Figure S47. ^{1}H NMR spectrum at 0 min (300 MHz, $CDCl_3$).

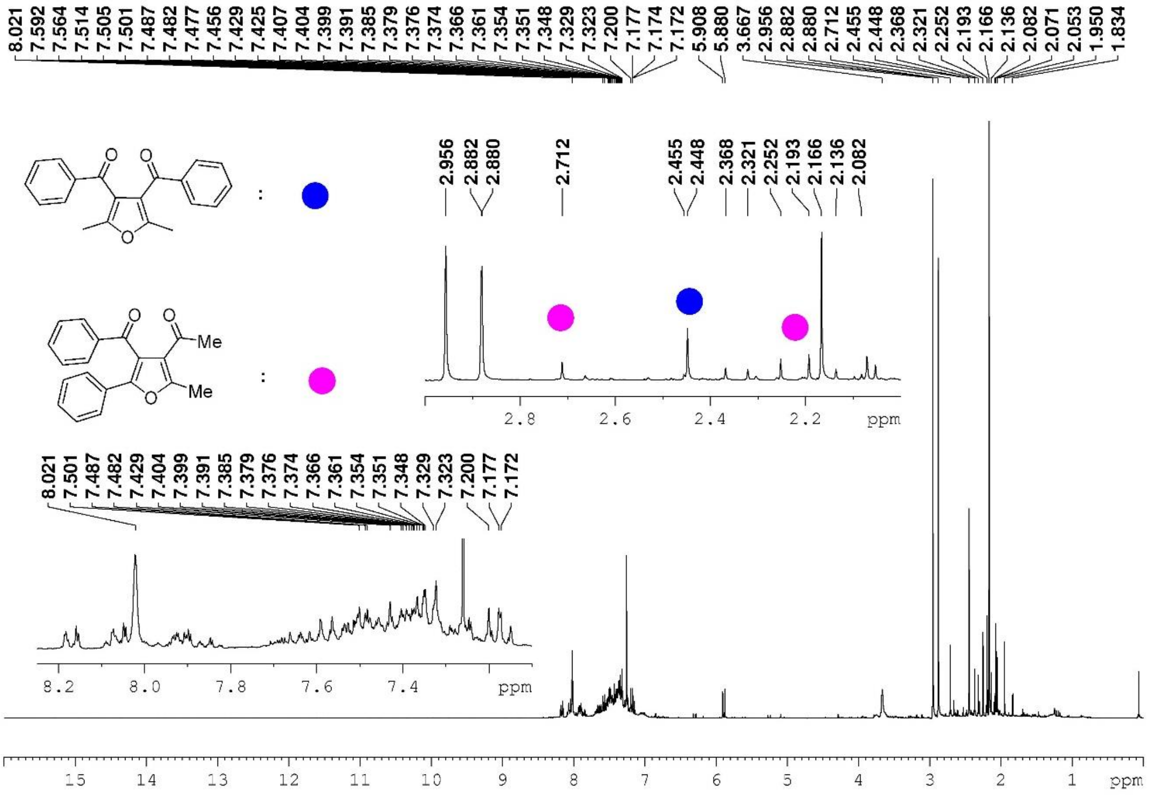

Figure S48. ^1H NMR spectrum after 5 h under irradiation (300 MHz, $CDCl_3$).

IX. NMR and HRMS data of the products

1-(4-benzoyl-2-methyl-5-phenyl-3-furanyl)-ethanone (1b)

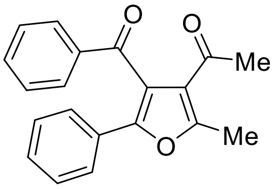

Isolated yield: 44% (66.9 mg, white solid).

^{1}H NMR (400 MHz, $CDCl_3$): δ 7.91 (d, *J* = 8.0 Hz, 2H), 7.56-7.50 (m, 3H), 7.43-7.39 (m, 2H), 7.29-7.24 (m, 3H), 2.72 (s, 3H), 2.25 (s, 3H).

^{13}C{^{1}H} NMR (101 MHz, $CDCl_3$): δ 193.5, 192.8, 157.0, 149.5, 137.5, 133.7, 129.4, 128.9, 128.8, 128.7, 128.6, 125.8, 125.0, 120.3, 30.0, 14.8.

HRMS: *m/z* for $[C_{20}H_{16}O_3]^+$ [M^+] calcd: 304.1099, found: 304.1085.

2,5-dimethyl-3,4-dibenzoyl-furan (1c)

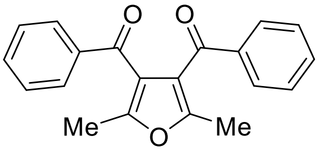

Isolated yield: 46% (69.9 mg, colorless liquid).

^{1}H NMR (400 MHz, $CDCl_3$): δ 7.37-7.33 (m, 6H), 7.20-7.16 (m, 4H), 2.45 (s, 6H).

^{13}C{^{1}H} NMR (101 MHz, $CDCl_3$): δ 191.3, 155.3, 139.0, 132.3, 128.3, 128.2, 121.7, 13.1.

HRMS: *m/z* for $[C_{20}H_{15}O_3]^+$ [M-H^+] calcd: 303.1021, found: 303.1010.

2,5-dimethyl-3,4-bis(3-methylbenzoyl)-furan (2c)

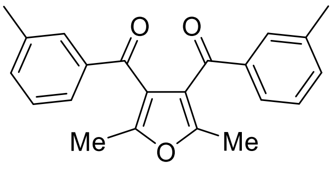

Isolated yield: 53% (88.0 mg, colorless liquid).

^{1}H NMR (400 MHz, CD_3CN): δ 7.32-7.27 (m, 6H), 7.21-7.16 (m, 2H), 2.31 (s, 6H), 2.23 (s, 6H).

^{13}C{^{1}H} NMR (101 MHz, CD_3CN): δ 190.6, 154.3, 138.6, 137.9, 132.7, 128.5, 127.9, 125.2, 121.7, 19.8, 12.0.

HRMS: *m/z* for $[C_{22}H_{21}O_3]^+$ [M+H^+] calcd: 333.1486, found: 333.1485.

1-[4-(4-methylbenzoyl)-2-methyl-5-(4-methylphenyl)-3-furanyl]-ethanone (3b)

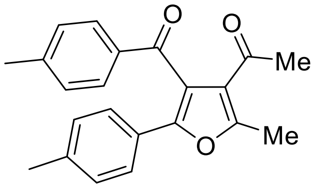

Isolated yield: 40% (66.4 mg, white solid).

^{1}H NMR (400 MHz, CD_3CN): δ 7.74 (d, *J* = 8.0 Hz, 2H), 7.36 (d, *J* = 8.0 Hz, 2H), 7.25 (d, *J* = 8.0 Hz, 2H), 7.13 (d, *J* = 8.0 Hz, 2H), 2.70 (s, 3H), 2.37 (s, 3H), 2.32 (s, 3H), 2.27 (s, 3H).

^{13}C{^{1}H} NMR (101 MHz, CD_3CN): δ 192.8, 192.4, 157.2, 148.5, 144.7, 138.9, 135.2, 129.5, 129.4, 129.1, 126.3, 125.3, 125.2, 120.2, 28.9, 20.7, 20.2, 14.2.

HRMS: *m/z* for $[C_{22}H_{20}O_3]^+$ [M^+] calcd: 332.1412, found: 332.1398.

2,5-dimethyl-3,4-bis(4-methylbenzoyl)-furan (3c)

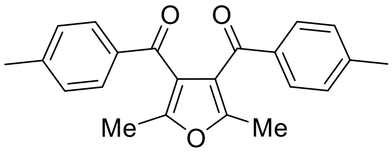

Isolated yield: 56% (93.0 mg, colorless liquid).

^{1}H NMR (400 MHz, CD_3CN): δ 7.47 (d, *J* = 8.0 Hz, 4H), 7.14 (d, *J* = 8.0 Hz, 4H), 2.33 (s, 6H), 2.27 (s, 6H).

^{13}C{^{1}H} NMR (101 MHz, CD_3CN): δ 190.5, 154.3, 143.6, 136.2, 129.0, 128.7, 122.1, 20.6, 12.4.

HRMS: *m/z* for $[C_{22}H_{19}O_3]^+$ [$M\text{-}H^+$] calcd: 331.1329, found: 331.1325.

1-[4-(4-methoxylbenzoyl)-2-methyl-5-(4-methoxylphenyl)-3-furanyl]-ethanone (4b)

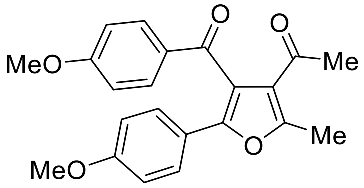

Isolated yield: 35% (63.7 mg, white solid).

^{1}H NMR (400 MHz, CD_3CN): δ 7.81 (d, *J* = 9.0 Hz, 2H), 7.41 (d, *J* = 9.1 Hz, 2H), 6.93 (d, *J* = 9.1 Hz, 2H), 6.86 (d, *J* = 9.0 Hz, 2H), 3.82 (s, 3H), 3.74 (s, 3H), 2.69 (s, 3H), 2.30 (s, 3H).

^{13}C{^{1}H} NMR (101 MHz, CD_3CN): δ 193.1, 192.3, 164.6, 160.5, 157.5, 149.0, 131.9, 131.4, 127.5, 125.6, 122.3, 119.9, 114.8, 114.5, 55.9, 55.6, 29.6, 14.7.

HRMS: *m/z* for $[C_{22}H_{20}O_5]^+$ $[M^+]$ calcd: 364.1311, found: 364.1299.

2,5-dimethyl-3,4-bis(4-methoxylbenzoyl)-furan (4c)

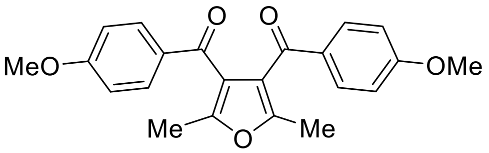

Isolated yield: 60% (109.2 mg, colorless liquid).

^{1}H NMR (400 MHz, CD_3CN): δ 7.56 (d, J = 9.1 Hz, 4H), 6.83 (d, J = 9.0 Hz, 4H), 3.80 (s, 6H), 2.28 (s, 6H).

^{13}C{^{1}H} NMR (101 MHz, CD_3CN): δ 190.0, 163.9, 154.4, 132.1, 131.5, 122.7, 114.1, 55.9, 12.9.

HRMS: *m/z* for $[C_{22}H_{21}O_5]^+$ $[M+H^+]$ calcd: 365.1384, found: 365.1389.

1-[4-(2,4,6-trimethylbenzoyl)-2-methyl-5-(2,4,6-trimethylphenyl)-3-furanyl]-ethanone (5b)

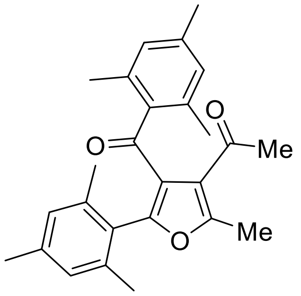

Isolated yield: 25% (48.5 mg, white solid).

^{1}H NMR (400 MHz, $CDCl_3$): δ 6.93 (s, 2H), 6.87 (s, 2H), 2.31 (s, 3H), 2.30 (s, 3H), 2.26 (s, 6H), 2.12 (s, 6H), 2.07 (s, 3H), 2.04 (s, 3H).

^{13}C{^{1}H} NMR (101 MHz, $CDCl_3$): δ 195.2, 194.5, 157.6, 153.0, 140.1, 139.3, 138.6, 137.7, 134.9, 129.0, 128.3, 126.0, 125.7, 123.7, 29.8, 21.3, 21.2, 19.9, 19.7, 13.0.

HRMS: *m/z* for $[C_{26}H_{29}O_3]^+$ $[M+H^+]$ calcd: 389.2112, found: 389.2109.

2,5-dimethyl-3,4-bis(4-fluorobenzoyl)-furan (6c)

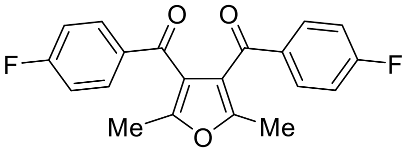

Isolated yield: 52% (88.4 mg, colorless liquid).

^{1}H NMR (400 MHz, $CDCl_3$): δ 7.42-7.39 (m, 4H), 6.91-6.87 (m, 4H), 2.45 (s, 6H).

^{19}**F{^{1}H} NMR** (376 MHz, $CDCl_3$): δ -106.1 (s, 2F).

^{13}**C{^{1}H} NMR** (101 MHz, $CDCl_3$): δ 189.6, 165.2 (d, J = 255 Hz), 155.5, 135.3 (d, J = 3 Hz), 130.9 (d, J = 9 Hz), 121.4, 115.4 (d, J = 22 Hz), 13.1.

HRMS: m/z for $[C_{20}H_{15}F_2O_3]^+$ $[M+H^+]$ calcd: 341.0984, found: 341.0989.

(2,5-diphenyl-3,4-furandiyl)-bis-phenyl-methanone (7b)

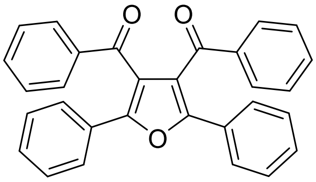

Isolated yield: 60% (128.4 mg, white solid).

^{1}**H NMR** (400 MHz, $CDCl_3$): δ 7.65-7.61 (m, 8H), 7.39-7.35 (m, 2H), 7.28-7.26 (m, 6H), 7.22-7.18 (m, 4H).

^{13}**C{^{1}H} NMR** (101 MHz, $CDCl_3$): δ 191.4, 152.9, 137.5, 133.3, 129.4, 129.3, 128.9, 128.6, 128.4, 127.2, 123.3.

HRMS: m/z for $[C_{30}H_{20}O_3]^+$ $[M^+]$ calcd: 428.1412, found: 428.1390.

[2,5-di(4-methoxylphenyl)-3,4-furandiyl)-bis-(4-methoxylphenyl)]-methanone (8b)

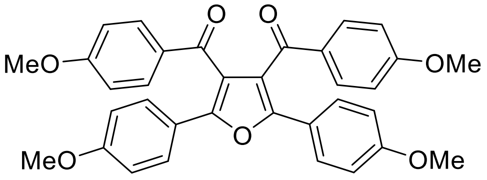

Isolated yield: 65% (178.2 mg, light yellow liquid).

^{1}**H NMR** (400 MHz, $CDCl_3$): δ 7.69-7.65 (m, 8H), 6.88-6.84 (m, 4H), 6.75-6.73 (m, 4H), 3.80 (s, 6H), 3.79 (s, 6H).

^{13}**C{^{1}H} NMR** (101 MHz, $CDCl_3$): δ 189.3, 162.6, 159.1, 150.7, 130.8, 129.8, 127.5, 121.1, 120.9, 113.0, 112.5, 54.4, 54.3.

HRMS: m/z for $[C_{34}H_{29}O_7]^+$ $[M+H^+]$ calcd: 549.1908, found: 549.1914.

[2-(4-*tert*-butyl-phenyl)-3-(4-*tert*-butylbenzoyl)-4-(4-methoxylbenzoyl)-5-(4-methoxylphenyl]-furan (9b)

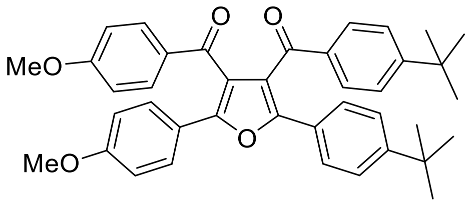

Isolated yield: 30% (90.0 mg, white solid).

^{1}H NMR (400 MHz, $CDCl_3$): δ 7.68-7.55 (m, 8H), 7.37-7.34 (m, 2H), 7.26-7.23 (m, 2H), 6.85 (d, *J* = 9.0 Hz, 2H), 6.72 (d, *J* = 9.0 Hz, 2H), 3.80 (s, 6H), 1.30 (s, 9H), 1.27 (s, 9H).

^{13}C{^{1}H} NMR (101 MHz, $CDCl_3$): δ 191.4, 190.3, 163.6, 160.3, 156.8, 152.8, 152.2, 151.8, 135.3, 131.7, 131.0, 129.4, 128.8, 126.7, 126.4, 125.5, 125.2, 122.9, 122.2, 121.9, 114.0, 113.5, 55.4, 55.3, 35.1, 34.8, 31.2, 31.0.

HRMS: *m/z* for $[C_{40}H_{41}O_5]^+$ $[M+H^+]$ calcd: 601.2949, found: 601.2953.

[2,5-di(4-*tert*-butylphenyl)-3,4-bis(4-methoxylbenzoyl)]-furan (9c)

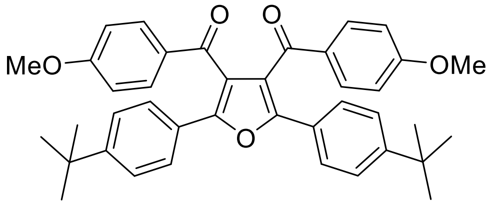

Isolated yield: 45% (135.1 mg, colorless liquid).

^{1}H NMR (400 MHz, $CDCl_3$): δ 7.68-7.64 (m, 8H), 7.35 (d, *J* = 9.1 Hz, 4H), 6.74 (d, *J* = 9.1 Hz, 4H), 3.80 (s, 6H), 1.30 (s, 18H).

^{13}C{^{1}H} NMR (101 MHz, $CDCl_3$): δ 189.3, 162.7, 151.2, 150.9, 130.9, 129.8, 125.6, 125.3, 124.5, 121.8, 112.5, 54.4, 33.7, 30.1.

HRMS: *m/z* for$[C_{40}H_{41}O_5]^+$ $[M+H^+]$ calcd: 601.2949, found: 601.2960.

[2,5-di(4-methoxylphenyl)-3,4-bis(4-*tert*-butylbenzoyl)]-furan (9d)

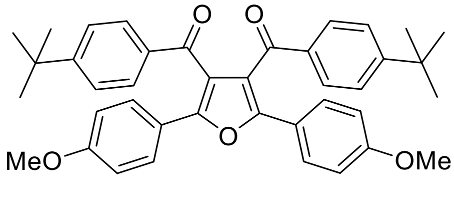

Isolated yield: 15% (45.0 mg, colorless liquid).

^{1}H NMR (400 MHz, $CDCl_3$): δ 7.96-7.87 (m, 8H), 7.42-7.40 (m, 4H), 6.87-6.85 (m, 4H), 3.83 (s, 6H), 1.29 (s, 18H).

$^{13}C\{^1H\}$ **NMR** (101 MHz, $CDCl_3$): δ 190.6, 164.4, 133.5, 132.5, 132.4, 129.9, 129.4, 125.8, 125.7, 114.0, 55.5, 35.3, 31.0.

HRMS: *m/z* for $[C_{40}H_{41}O_5]^+$ $[M+H^+]$ calcd: 601.2949, found: 601.2963.

2,5-dimethyl-3,4-bis(4-phenylbenzoyl)-furan (10c)

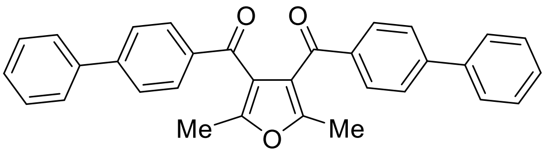

Isolated yield: 52% (118.6 mg, white solid).

^{1}**H NMR** (400 MHz, CD_3CN): δ 7.62-7.60 (m, 4H), 7.57-7.53 (m, 8H), 7.48-7.44 (m, 4H), 7.42-7.40 (m, 2H), 2.37 (s, 6H).

$^{13}C\{^1H\}$ **NMR** (101 MHz, CD_3CN): δ 191.0, 155.6, 145.4, 140.2, 138.4, 129.7, 129.6, 128.8, 127.7, 127.4, 122.5, 13.0.

HRMS: *m/z* for $[C_{32}H_{23}O_3]^+$ $[M-H^+]$ calcd: 455.1642, found: 455.1618.

1-[4-(4-phenoxybenzoyl)-2-methyl-5-(4-phenoxyphenyl)-3-furanyl]-ethanone (11b)

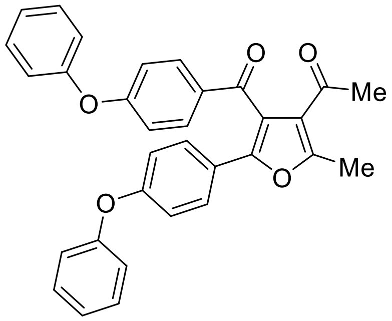

Isolated yield: 30% (73.2 mg, white solid).

^{1}**H NMR** (400 MHz, CD_3CN): δ 7.82 (d, J = 9.1 Hz, 2H), 7.47-7.35 (m, 6H), 7.26-7.21 (m, 1H), 7.18-7.14 (m, 1H), 7.08-7.06 (m, 2H), 7.00-6.98 (m, 2H), 6.94-6.90 (m, 4H), 2.69 (s, 3H), 2.33 (s, 3H).

$^{13}C\{^1H\}$ **NMR** (101 MHz, CD_3CN): δ 193.0, 192.2, 163.0, 158.4, 157.8, 156.9, 155.8, 148.8, 133.0, 132.0, 130.8, 130.6, 127.8, 125.7, 125.5, 124.6, 124.5, 120.9, 120.6, 119.9, 119.0, 117.6, 29.5, 14.7.

HRMS: *m/z* for $[C_{32}H_{25}O_5]^+$ $[M+H^+]$ calcd: 489.1697, found: 489..1708.

2,5-dimethyl-3,4-bis(4-phenoxybenzoyl)-furan (11c)

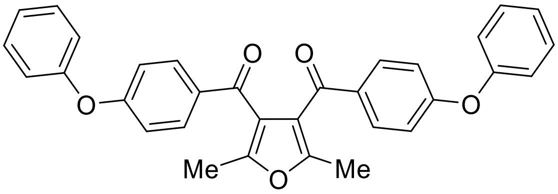

Isolated yield: 43% (105.0 mg, white solid).

^{1}H NMR (400 MHz, CD_3CN): δ 7.51-7.49 (m, 4H), 7.41-7.37 (m, 4H), 7.22-7.18 (m, 2H), 7.01-6.99 (m, 4H), 6.85-6.81 (m, 4H), 2.32 (s, 6H).

^{13}C{^{1}H} NMR (101 MHz, CD_3CN): δ 190.0, 161.9, 156.1, 155.2, 134.2, 131.4, 130.7, 125.2, 122.3, 120.4, 117.7, 13.0.

HRMS: *m/z* for $[C_{32}H_{23}O_5]^+$ $[M\text{-}H^+]$ calcd: 487.1540, found: 487.1514.

[2-methyl-3-(methyl-carbonyl)-4-(naphthalene-2-carbonyl)-5-(2-naphthalene)]-furan (12b)

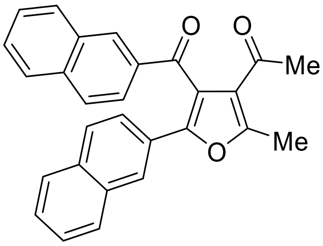

Isolated yield: 20% (40.4 mg, white solid).

^{1}H NMR (400 MHz, CD_3CN): δ 8.38 (s, 1H), 8.10-8.07 (m, 2H), 7.98-7.89 (m, 3H), 7.79-7.76 (m, 3H), 7.63-7.50 (m, 3H), 7.47-7.45 (m, 2H), 2.80 (s, 3H), 2.36 (s, 3H).

^{13}C{^{1}H} NMR (101 MHz, CD_3CN): δ 193.7, 193.1, 158.5, 136.3, 135.8, 133.6, 133.5, 133.1, 132.1, 130.0, 129.4, 129.2, 129.1, 128.7, 128.3, 128.2, 127.6, 127.5, 127.0, 126.2, 125.2, 124.5, 123.4, 121.9, 29.6, 14.9.

HRMS: *m/z* for $[C_{28}H_{20}O_3]^+$ $[M^+]$ calcd: 404.1412, found: 404.1388.

2,5-dimethyl-3,4-bis(naphthalene-2-carbonyl)-furan (12c)

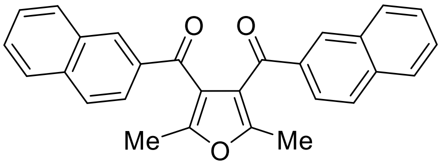

Isolated yield: 40% (80.8 mg, white solid).

^{1}H NMR (400 MHz, CD_3CN): δ 8.15 (s, 2H), 8.03-7.95 (m, 2H), 7.82-7.78 (m, 4H), 7.69-7.67 (m, 2H), 7.53-7.45 (m, 4H), 2.36 (s, 6H).

$^{13}C\{^1H\}$ NMR (101 MHz, CD_3CN): δ 191.3, 155.5, 136.7, 135.7, 132.8, 131.0, 129.6, 129.0, 128.8, 128.2, 127.4, 124.5, 122.8, 13.1.

HRMS: *m/z* for $[C_{28}H_{20}O_3]^+$ $[M^+]$ calcd: 404.1412, found: 404.1359.

[2-methyl-3-(methyl-carbonyl)-4-(2,3-dihydro-1,4-benzodioxin-6-yl-methanone)-5-(2,3-dihydro -1,4-benzodioxin)]-furan (13b)

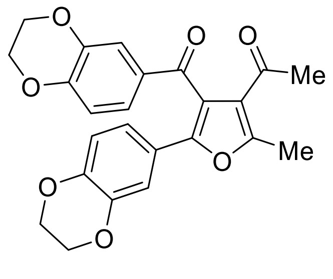

Isolated yield: 22% (46.2 mg, white solid).

^1H NMR (400 MHz, CD_3CN): δ 7.37-7.34 (m, 2H), 7.22-7.21 (m, 1H), 7.17-7.14 (m, 1H), 6.95-6.93 (m, 2H), 4.31-4.27 (m, 8H), 2.22 (s, 3H), 2.10 (s, 3H).

$^{13}C\{^1H\}$ NMR (101 MHz, CD_3CN): δ 196.0, 190.4, 153.7, 153.4, 149.1, 145.8, 144.3, 144.2, 132.5, 124.2, 123.9, 123.2, 122.7, 122.1, 118.6, 117.6, 65.4, 65.2, 64.9, 64.8, 30.3, 12.9.

HRMS: *m/z* for $[C_{24}H_{21}O_7]^+$ $[M+H^+]$ calcd: 421.1282, found: 421.1287.

2,5-dimethyl-3,4-bis(2,3-dihydro-1,4-benzodioxin-6-yl-methanone)-furan (13c)

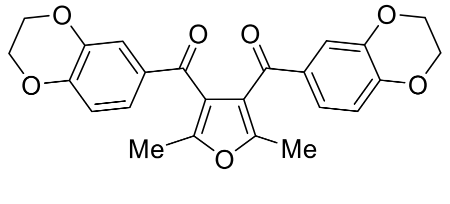

Isolated yield: 40% (80.8 mg, white solid).

^1H NMR (400 MHz, CD_3CN): δ 7.00-6.97 (m, 2H), 6.90 (d, J = 2.0 Hz, 2H), 6.73 (d, J = 8.0 Hz, 2H), 4.26-4.19 (m, 8H), 2.32 (s, 6H).

$^{13}C\{^1H\}$ NMR (101 MHz, CD_3CN): δ 189.4, 154.3, 147.7, 143.4, 132.7, 122.5, 121.9, 117.2, 116.7, 64.7, 64.2, 12.3.

HRMS: *m/z* for $[C_{24}H_{21}O_7]^+$ $[M+H^+]$ calcd: 421.1282, found: 421.1296.

[2-methyl-3-(methyl-carbonyl)-4-(furan-2-carbonyl)-5-furanyl]-furan (14b)

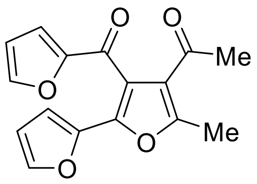

Isolated yield: 40% (56.8 mg, white solid).

^{1}H NMR (400 MHz, $CDCl_3$): δ 7.62 (d, J = 1.1 Hz, 1H), 7.56 (d, J = 1.1 Hz, 1H), 7.23 (d, J = 4.0 Hz, 1H), 7.18 (d, J = 3.1 Hz, 1H), 6.60-6.53 (m, 2H), 2.46 (s, 3H), 2.25 (s, 3H).

^{13}C{^{1}H} NMR (101 MHz, $CDCl_3$): δ 194.3, 177.8, 155.3, 153.0, 146.9, 144.6, 144.0, 143.7, 122.8, 121.0, 119.3, 112.9, 112.5, 111.9, 30.3, 13.2.

HRMS: *m/z* for $[C_{16}H_{13}O_5]^+$ $[M+H^+]$ calcd: 285.0758, found: 285.0759.

2,5-dimethyl-3,4-bis(furan-2-carbonyl)-furan (14c)

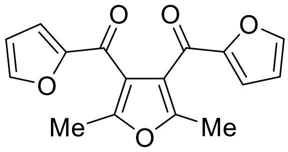

Isolated yield: 46% (65.3 mg, white solid).

^{1}H NMR (400 MHz, $CDCl_3$): δ 7.38 (d, J = 1.0 Hz, 2H), 6.97 (d, J = 4.1 Hz, 2H), 6.36-6.35 (m, 2H), 2.48 (s, 6H).

^{13}C{^{1}H} NMR (101 MHz, $CDCl_3$): δ 177.8, 155.6, 153.0, 146.1, 120.2, 117.8, 112.3, 13.1.

HRMS: *m/z* for $[C_{16}H_{13}O_5]^+$ $[M+H^+]$ calcd: 285.0758, found: 285.0759.

[2-methyl-3-(methyl-carbonyl)-4-(thiophene-2-carbonyl)-5-thienyl]-furan (15b)

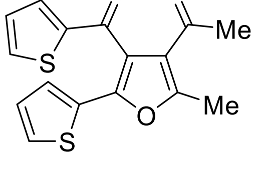

Isolated yield: 43% (67.9 mg, white solid).

^{1}H NMR (400 MHz, CD_3CN): δ 8.08-8.07 (m, 1H), 8.01-8.00 (m, 1H), 7.53-7.47 (m, 4H), 2.30 (s, 3H), 2.12 (s, 3H).

^{13}C{^{1}H} NMR (101 MHz, CD_3CN): δ 196.0, 185.4, 154.3, 149.7, 143.5, 135.3, 130.6, 128.1, 127.6, 127.4, 127.2, 126.5, 123.7, 123.4, 30.4, 13.1.

HRMS: *m/z* for $[C_{16}H_{12}O_3S_2]^+$ $[M^+]$ calcd: 316.0228, found: 316.0220.

2,5-dimethyl-3,4-bis(thiophene-2-carbonyl)-furan (15c)

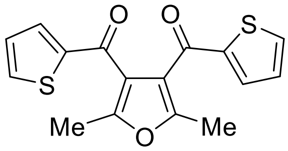

Isolated yield: 47% (74.3 mg, white solid).

^{1}H NMR (400 MHz, CD_3CN): δ 7.83-7.82 (m, 2H), 7.32-7.30 (m, 2H), 7.21-7.20 (m, 2H), 2.34 (s, 6H).

^{13}C{^{1}H} NMR (101 MHz, CD_3CN): δ 184.9, 154.8, 143.8, 133.6, 127.5, 127.3, 123.1, 12.9.

HRMS: *m/z* for $[C_{16}H_{12}O_3S_2]^+$ [M^+] calcd: 316.0228, found: 316.0223.

1-[2-ethoxyl-3-carboethoxy-2,5-dihydro-5-phenyl-3-furanyl]-benzophenone (16b)

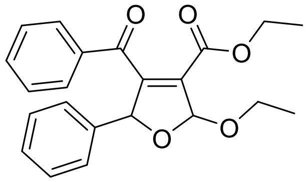

Isolated yield: 50% (91.5 mg, white solid).

^{1}H NMR (400 MHz, $CDCl_3$): δ 7.95-7.93 (m, 2H), 7.88-7.86 (m, 2H), 7.38-7.33 (m, 2H), 7.30-7.22 (m, 4H), 5.38 (s, 1H), 5.31 (s, 1H), 3.93-3.87 (m, 2H), 3.76-3.70 (m, 2H), 0.91 (t, J = 7.1 Hz, 3H), 0.73 (t, J = 7.1 Hz, 3H).

^{13}C{^{1}H} NMR (101 MHz, $CDCl_3$): δ 194.0, 193.2, 167.6, 167.1, 136.2, 135.8, 133.9, 133.8, , 129.5, 129.4, 128.7, 128.6, 62.1, 62.0, 54.2, 53.3, 13.9, 13.6.

HRMS: *m/z* for $[C_{22}H_{21}O_5]^+$ [$M\text{-}H^+$] calcd: 365.1384, found: 365.1380.

1-[2,5-di(ethoxyl)-3-benzoyl-2,3-dihydro-3-furanyl]-benzophenone (16c)

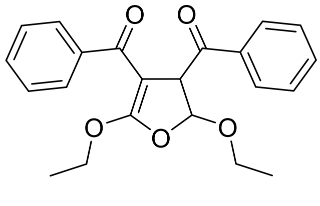

Isolated yield: 22% (40.3 mg, white solid).

^{1}H NMR (400 MHz, $CDCl_3$): δ 8.08 (d, J = 8.0 Hz, 1H), 7.90 (s, 1H), 7.43-7.34 (m, 7H), 7.24-7.22 (m, 1H), 4.28 (q, J = 7.1 Hz, 2H), 4.19 (q, J = 7.1 Hz, 2H), 3.53 (s, 2H), 1.34 (t, J = 7.1 Hz, 3H), 1.27 (t, J = 7.1 Hz, 3H).

^{13}C{^{1}H} NMR (101 MHz, $CDCl_3$): δ 207.0, 171.2, 141.7, 135.1, 129.0, 128.8, 128.6, 126.4, 125.8,

120.3, 119.4, 110.6, 61.1, 61.0, 33.7, 30.9, 14.3, 14.2.

HRMS: *m/z* for $[C_{22}H_{21}O_5]^+$ [M-H$^+$] calcd: 365.1384, found: 365.1378.

1-[2-ethoxyl-3-carboethoxy-2,5-dihydro-5-(4-fluorophenyl)-3-furanyl]-4-fluorobenzophenone (17b)

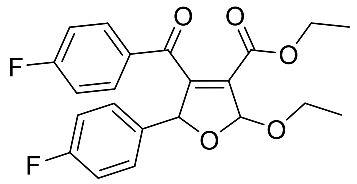

Isolated yield: 45% (90.5 mg, white solid).

^{1}H NMR (400 MHz, $CDCl_3$): δ 8.34-8.30 (m, 2H), 8.27-8.24 (m, 2H), 7.32-7.24 (m, 4H), 5.66 (s, 1H), 5.60 (s, 1H), 4.26-4.24 (m, 2H), 4.10-4.08 (m, 2H), 1.09 (t, *J* = 7.1 Hz, 3H), 1.02 (t, *J* = 7.1 Hz, 3H).

^{19}F{^{1}H} NMR (376 MHz, $CDCl_3$): δ -104.1 (s, 1F), -104.4 (s, 1F).

^{13}C{^{1}H} NMR (101 MHz, $CDCl_3$): δ 192.4, 191.7, 167.1 (d, *J* = 38 Hz), 166.3 (d, *J* = 256 Hz), 166.2 (d, *J* = 256 Hz), 132.4 (d, *J* = 10 Hz), 132.3 (d, *J* = 10 Hz), 132.2 (d, *J* = 2 Hz), 132.1 (d, *J* = 2 Hz), 115.8 (d, *J* = 22 Hz), 85.5, 82.9, 62.3, 62.2, 54.2, 53.2, 13.9, 13.7.

HRMS: *m/z* for $[C_{22}H_{19}F_2O_5]^+$ [M-H$^+$] calcd: 401.1196, found: 401.1180.

1-[2,5-di(ethoxyl)-3-(4-fluorobenzoyl)-2,5-dihydro-3-furanyl]-4-fluorobenzophenone (17c)

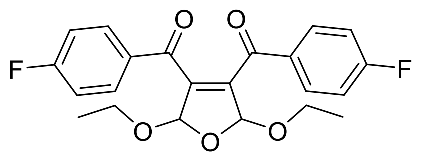

Isolated yield: 25% (50.3 mg, white solid).

^{1}H NMR (400 MHz, $CDCl_3$): δ 8.00-7.96 (m, 4H), 7.17-7.13 (m, 4H), 4.21 (q, *J* = 7.1 Hz, 4H), 3.96 (s, 2H), 1.26 (t, *J* = 7.1 Hz, 6H).

^{19}F{^{1}H} NMR (376 MHz, $CDCl_3$): δ -104.4 (s, 2F).

^{13}C{^{1}H} NMR (101 MHz, $CDCl_3$): δ 190.9, 166.1 (d, *J* = 249 Hz), 132.5 (d, *J* = 3 Hz), 131.2 (d, *J* = 10 Hz), 116.0 (d, *J* = 22 Hz), 87.2, 61.6, 46.0, 14.1.

HRMS: *m/z* for $[C_{22}H_{19}F_2O_5]^+$ [M-H$^+$] calcd: 401.1196, found: 401.1183.

X. NMR spectra of the products

1-(4-benzoyl-2-methyl-5-phenyl-3-furanyl)-ethanone (1b)

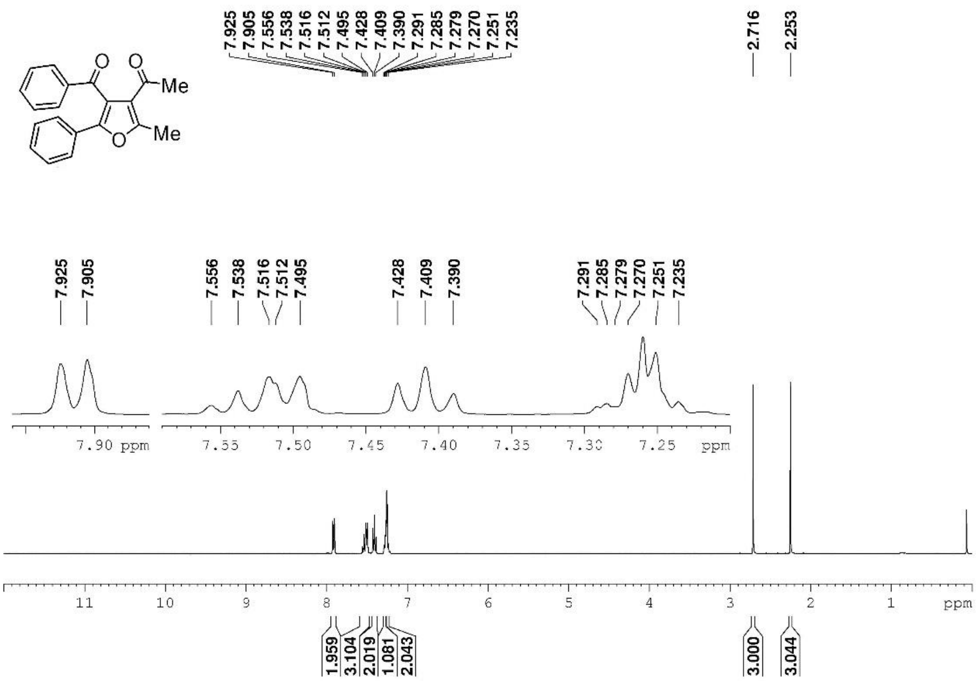

Figure S49. ^{1}H NMR spectrum of **1b** (400 MHz, $CDCl_3$).

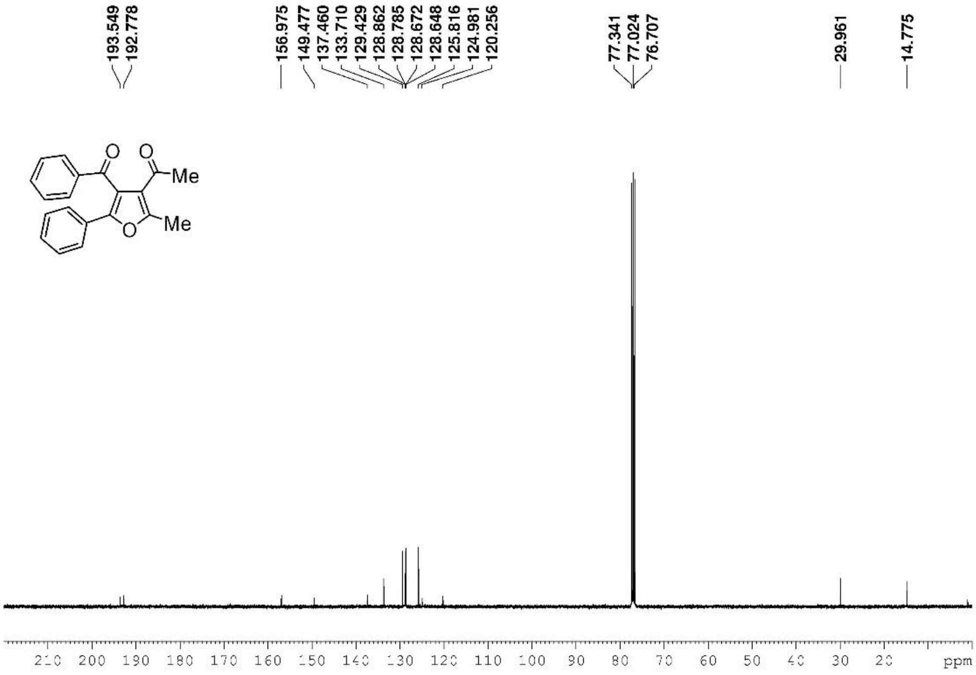

Figure S50. $^{13}C\{^{1}H\}$ NMR spectrum of **1b** (101 MHz, $CDCl_3$).

2,5-dimethyl-3,4-dibenzoyl-furan (1c)

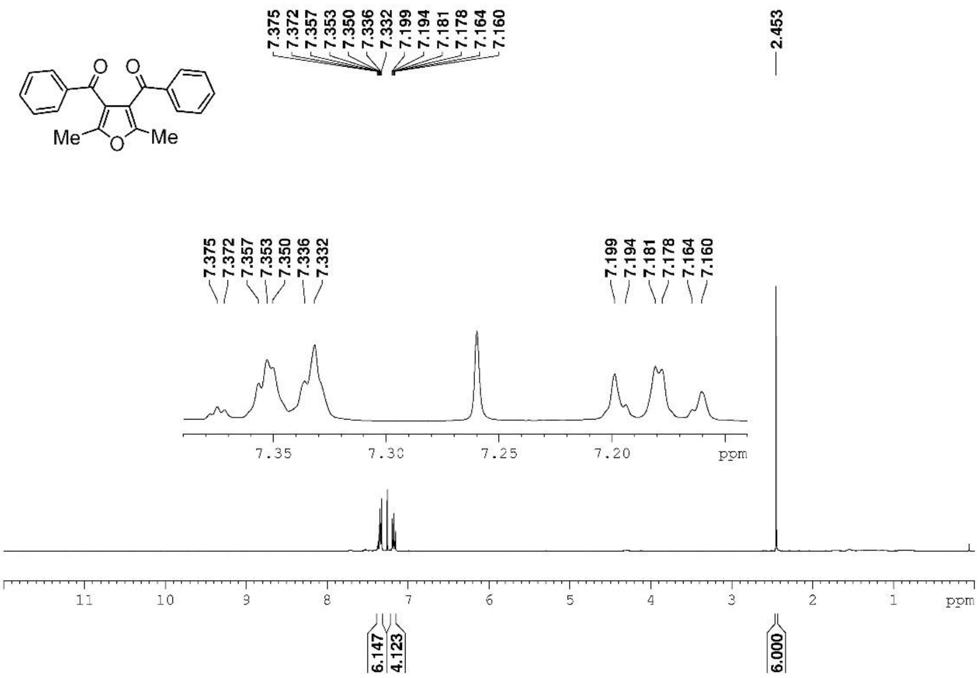

Figure S51. ^{1}H NMR spectrum of **1c** (400 MHz, $CDCl_3$).

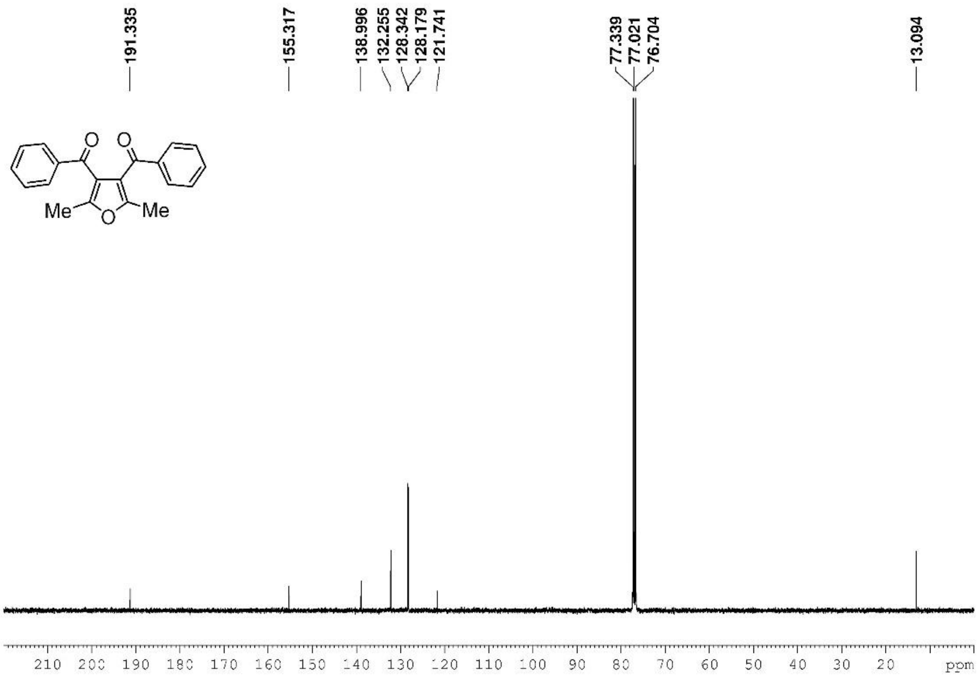

Figure S52. $^{13}C\{^{1}H\}$ NMR spectrum of **1c** (101 MHz, $CDCl_3$).

2,5-dimethyl-3,4-bis(3-methylbenzoyl)-furan (2c)

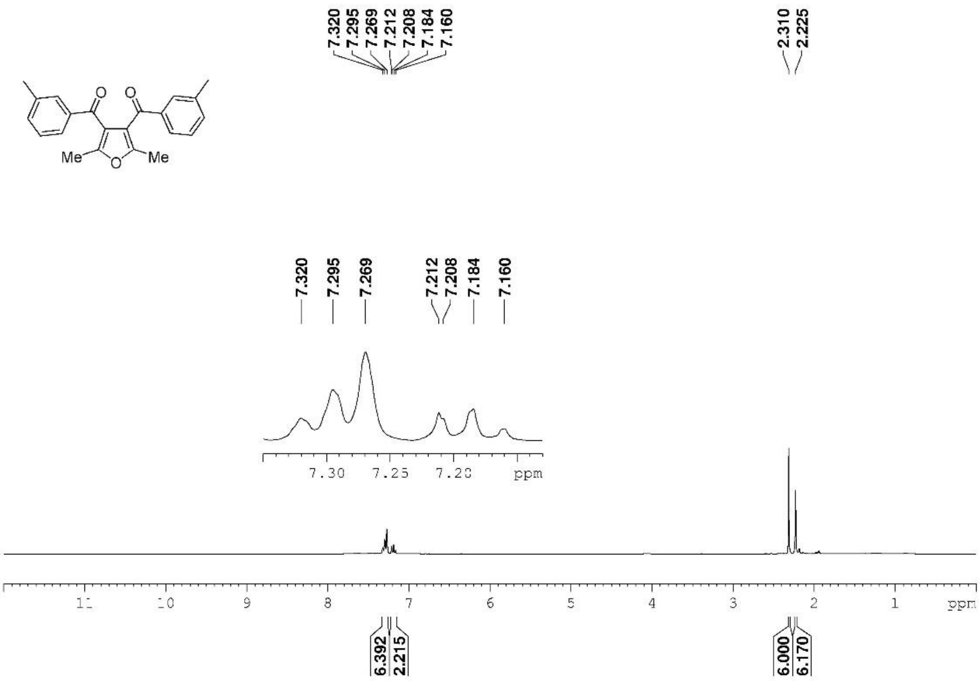

Figure S53. ^{1}H NMR spectrum of **2c** (400 MHz, CD_3CN).

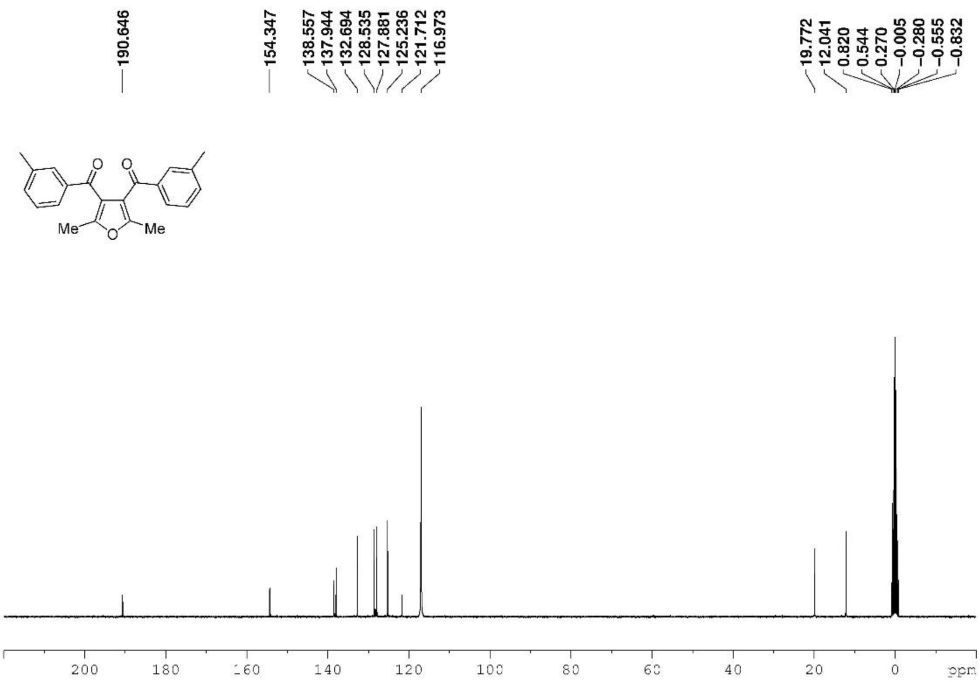

Figure S54. $^{13}C\{^{1}H\}$ NMR spectrum of **2c** (101 MHz, CD_3CN).

1-[4-(4-methylbenzoyl)-2-methyl-5-(4-methylphenyl)-3-furanyl]-ethanone (3b)

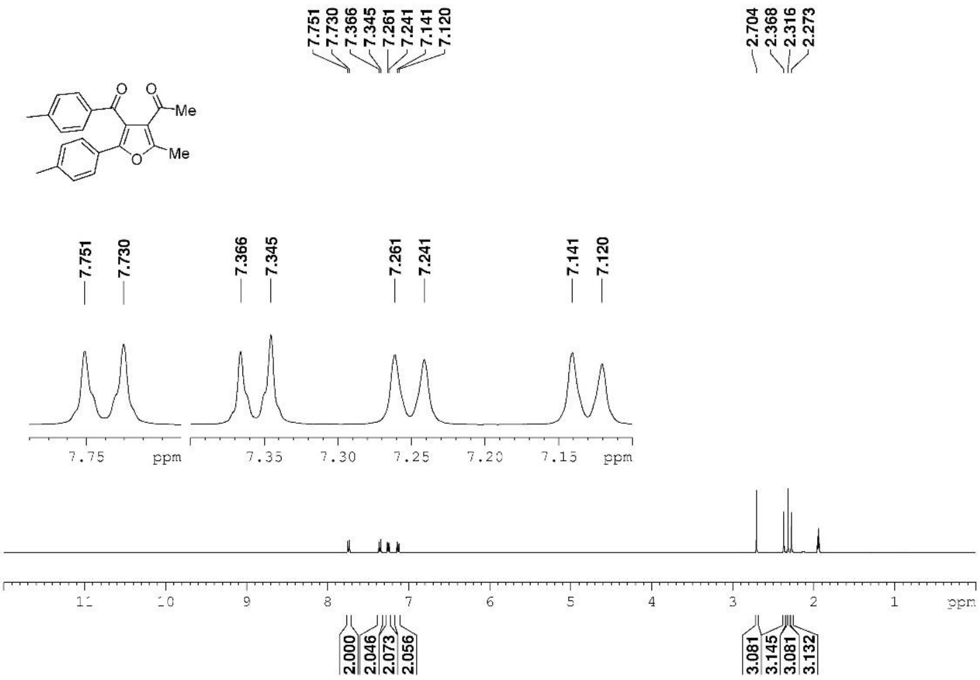

Figure S55. ^{1}H NMR spectrum of **3b** (400 MHz, CD_3CN).

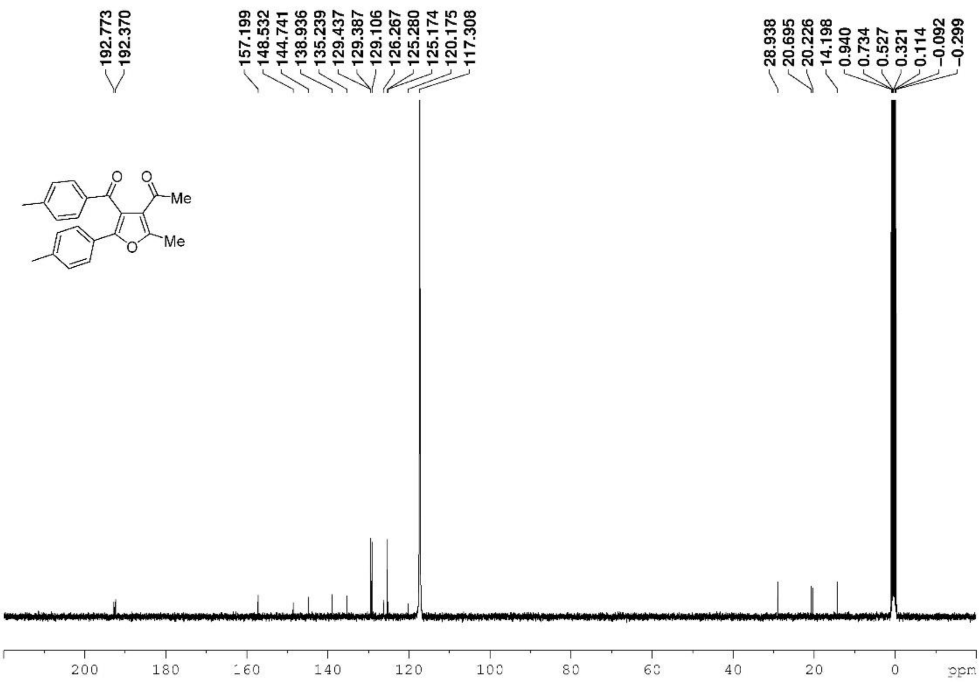

Figure S56. $^{13}C\{^{1}H\}$ NMR spectrum of **3b** (101 MHz, CD_3CN).

2,5-dimethyl-3,4-bis(4-methylbenzoyl)-furan (3c)

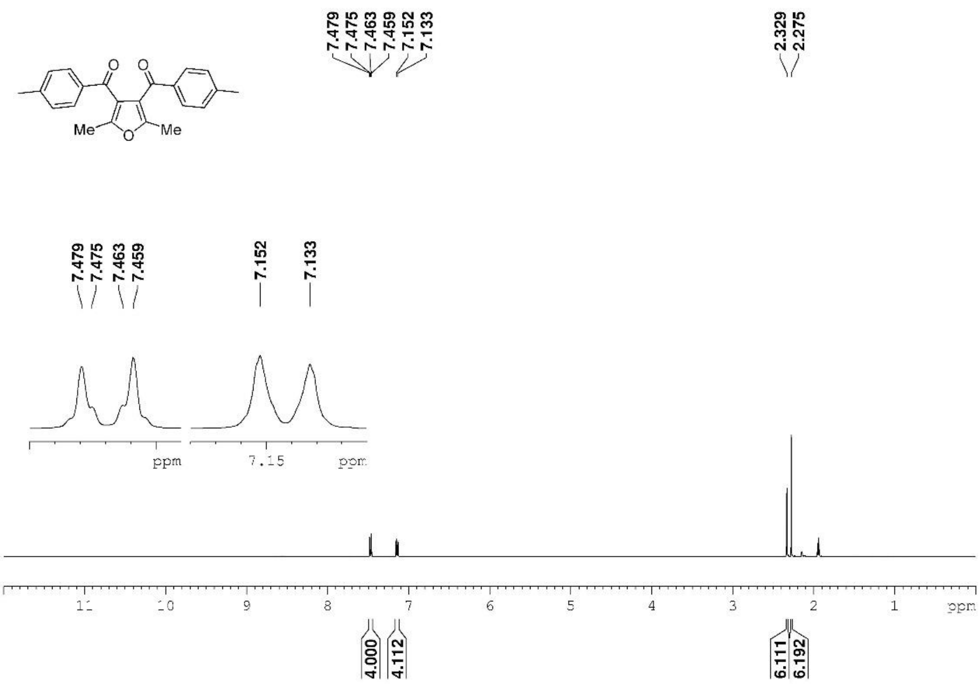

Figure S57. ^{1}H NMR spectrum of **3c** (400 MHz, CD_3CN).

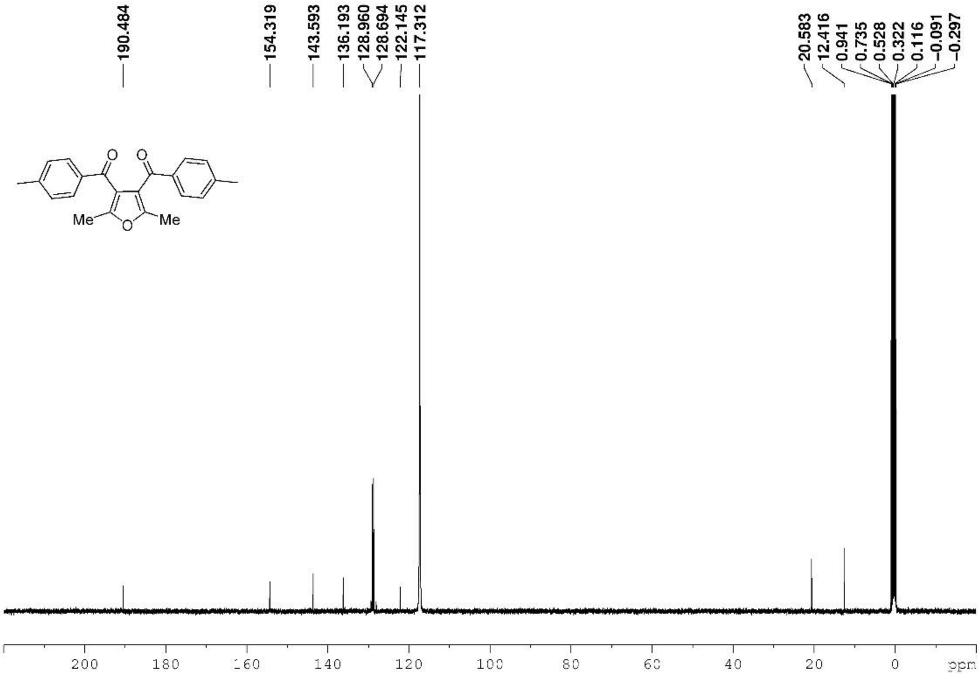

Figure S58. $^{13}C\{^{1}H\}$ NMR spectrum of **3c** (101 MHz, CD_3CN).

1-[4-(4-methoxylbenzoyl)-2-methyl-5-(4-methoxylphenyl)-3-furanyl]-ethanone (4b)

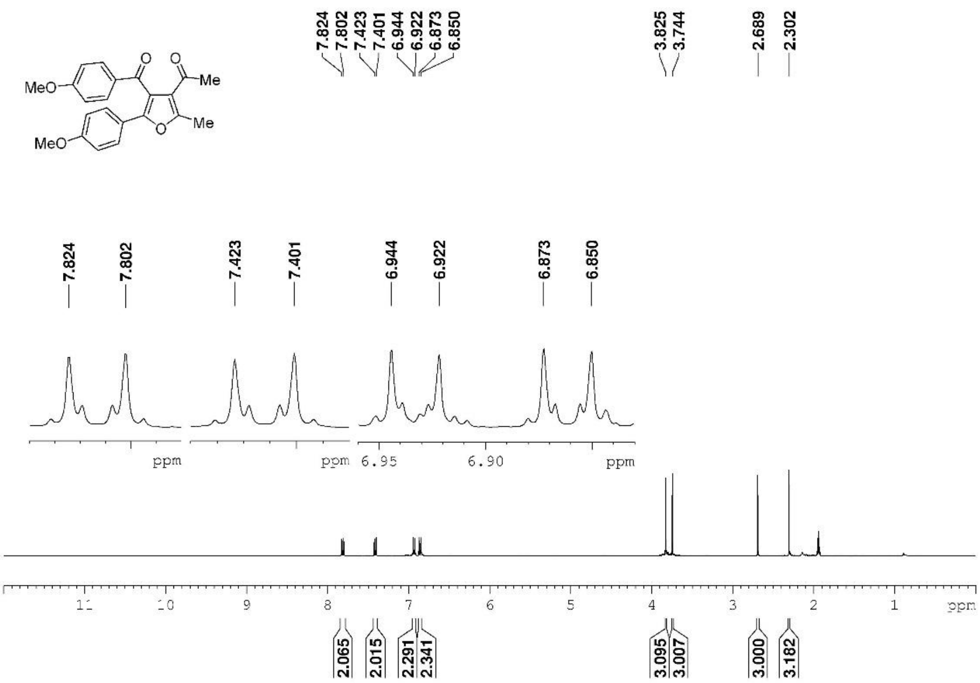

Figure S59. ^{1}H NMR spectrum of **4b** (400 MHz, CD_3CN).

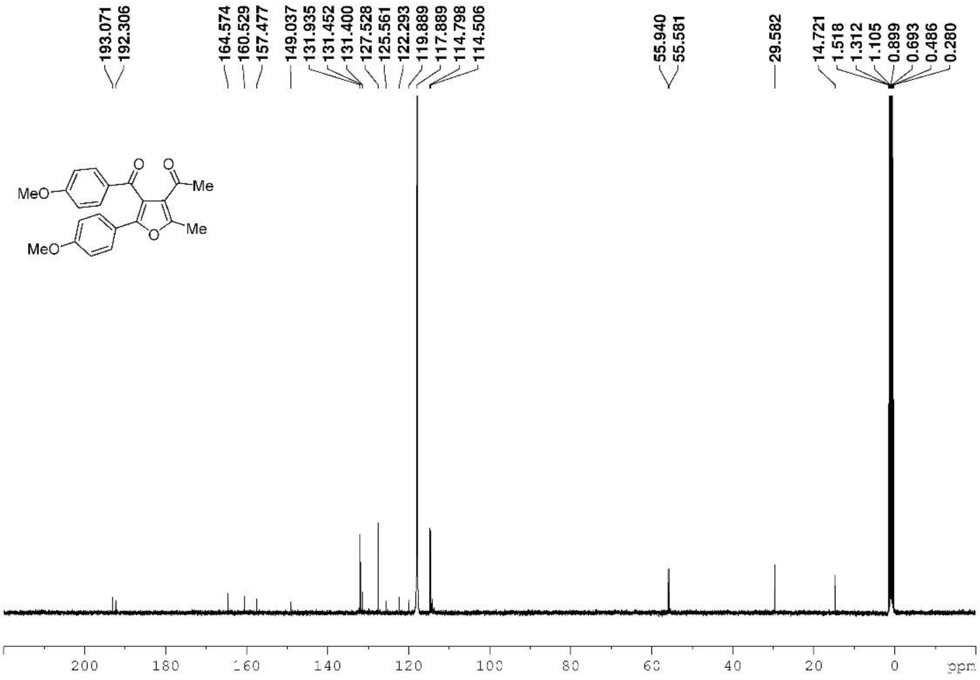

Figure S60. $^{13}C\{^{1}H\}$ NMR spectrum of **4b** (101 MHz, CD_3CN).

2,5-dimethyl-3,4-bis(4-methoxylbenzoyl)-furan (4c)

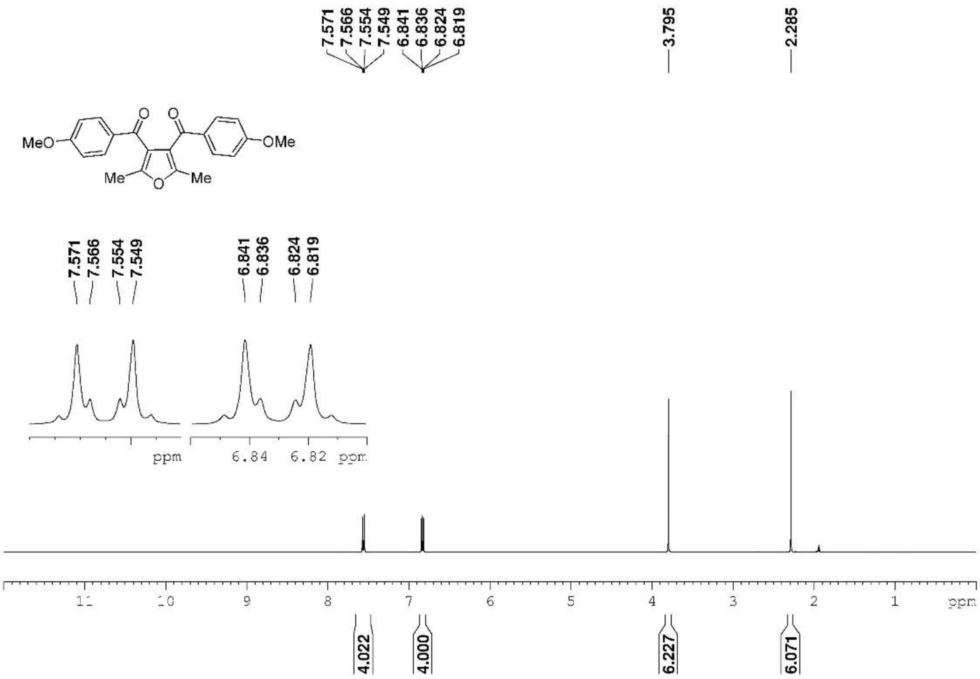

Figure S61. ^{1}H NMR spectrum of **4c** (400 MHz, CD_3CN).

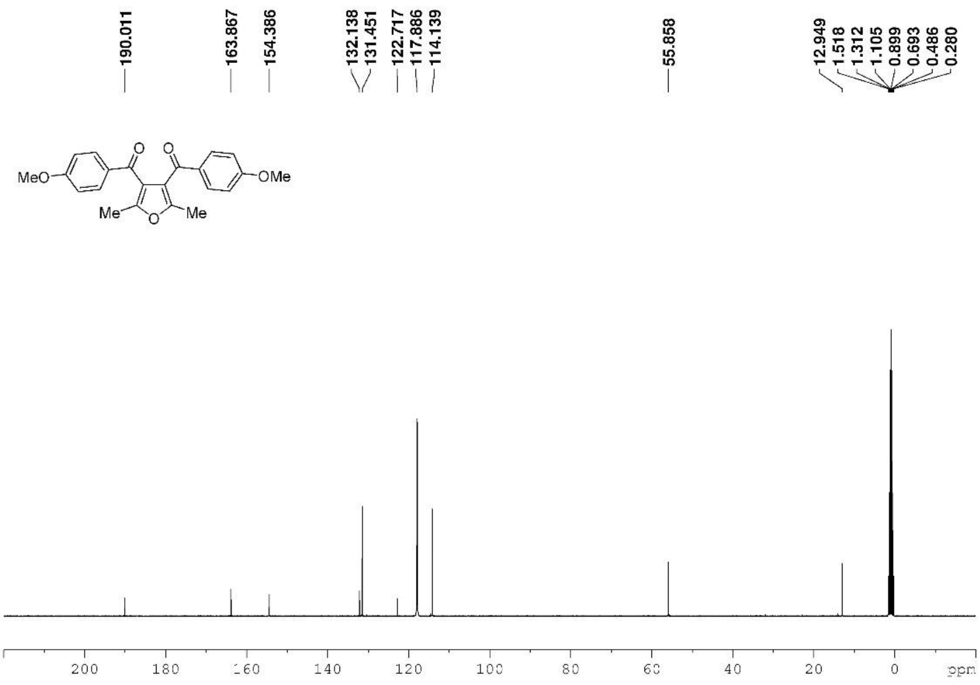

Figure S62. $^{13}C\{^1H\}$ NMR spectrum of **4c** (101 MHz, CD_3CN).

1-[4-(2,4,6-trimethylbenzoyl)-2-methyl-5-(2,4,6-trimethylphenyl)-3-furanyl]-ethanone (5b)

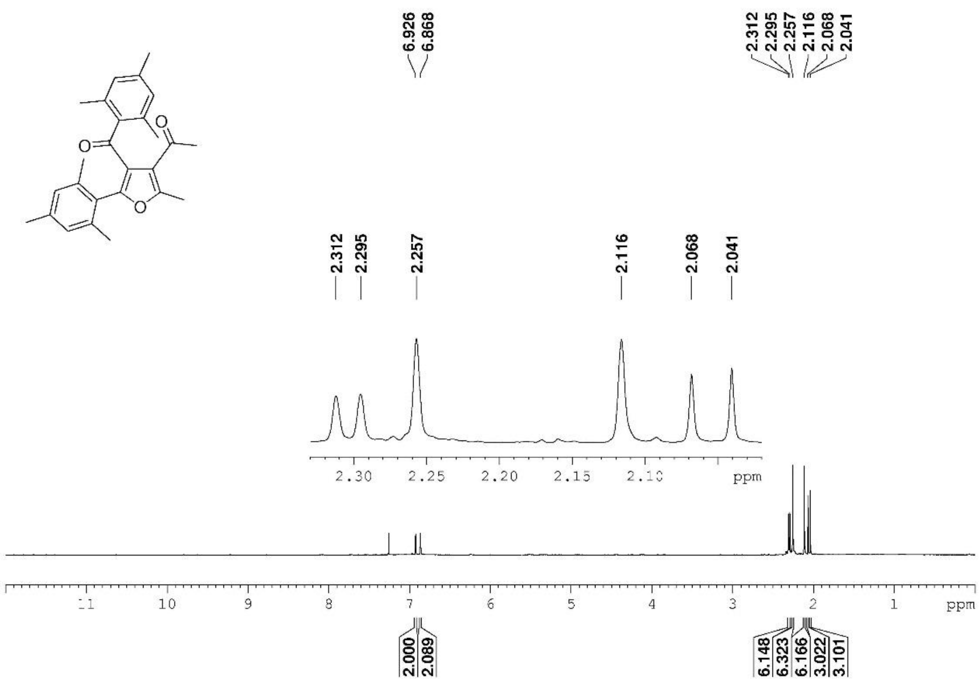

Figure S63. ^{1}H NMR spectrum of **5b** (400 MHz, $CDCl_3$).

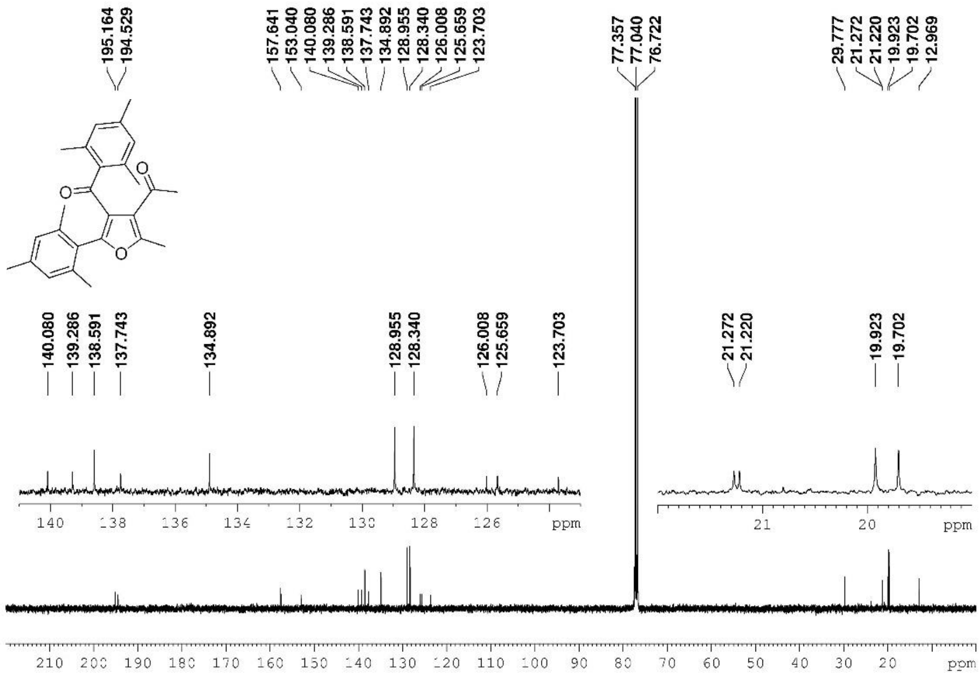

Figure S64. $^{13}C\{^{1}H\}$ NMR spectrum of **5b** (101 MHz, $CDCl_3$).

2,5-dimethyl-3,4-bis(4-fluorobenzoyl)-furan (6c)

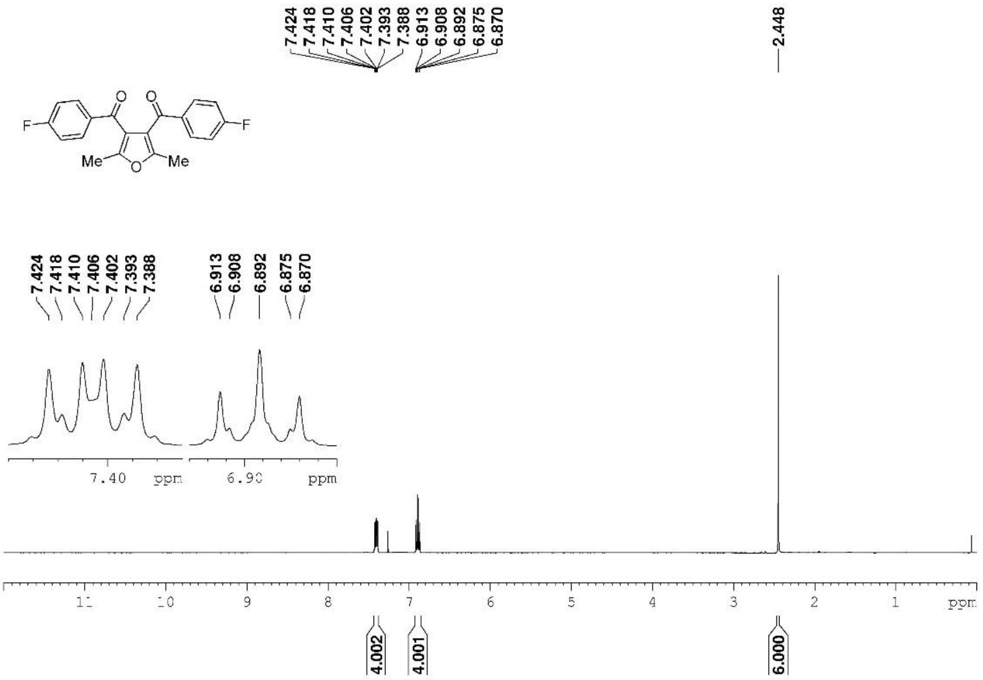

Figure S65. ^{1}H NMR spectrum of **6c** (400 MHz, $CDCl_3$).

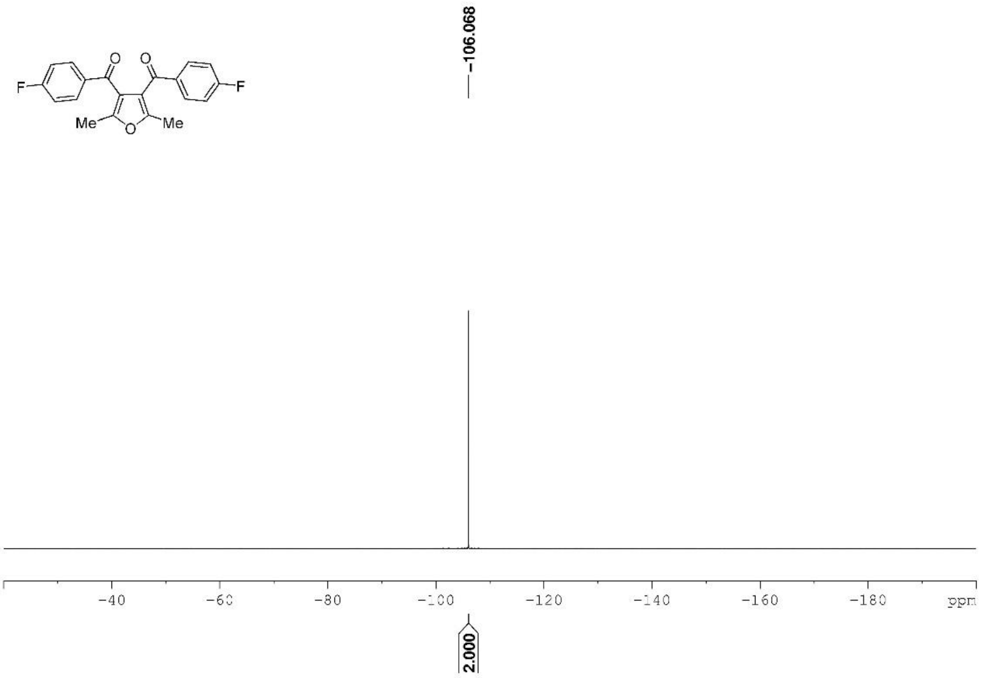

Figure S66. ^{19}F{^{1}H} NMR spectrum of **6c** (376 MHz, $CDCl_3$).

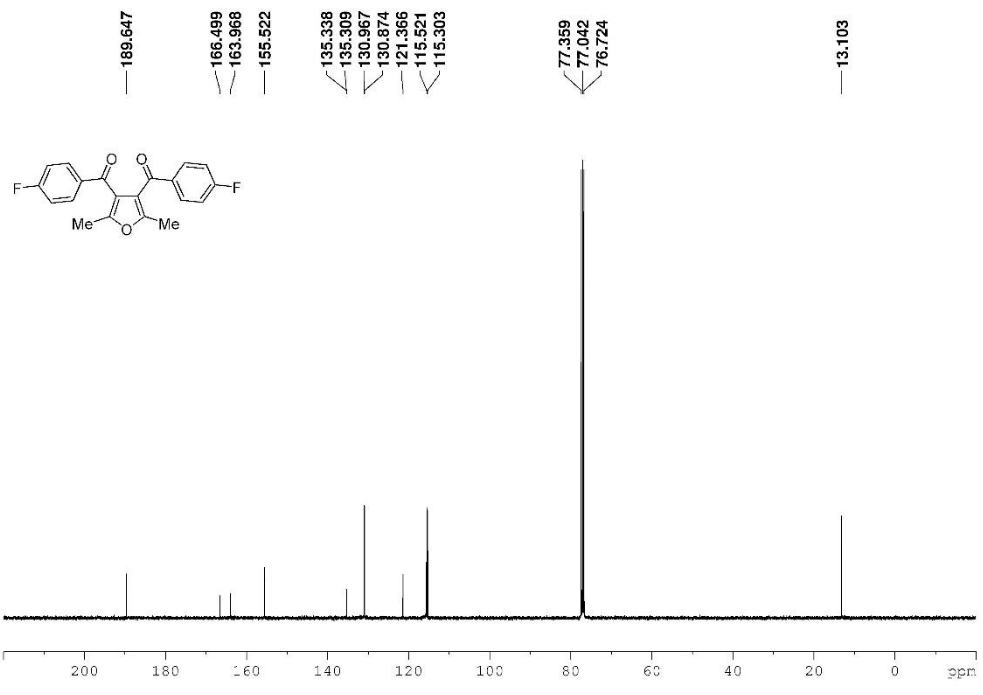

Figure S67. $^{13}C\{^1H\}$ NMR spectrum of **6c** (101 MHz, $CDCl_3$).

(2,5-diphenyl-3,4-furandiyl)-bis-phenyl-methanone (7b)

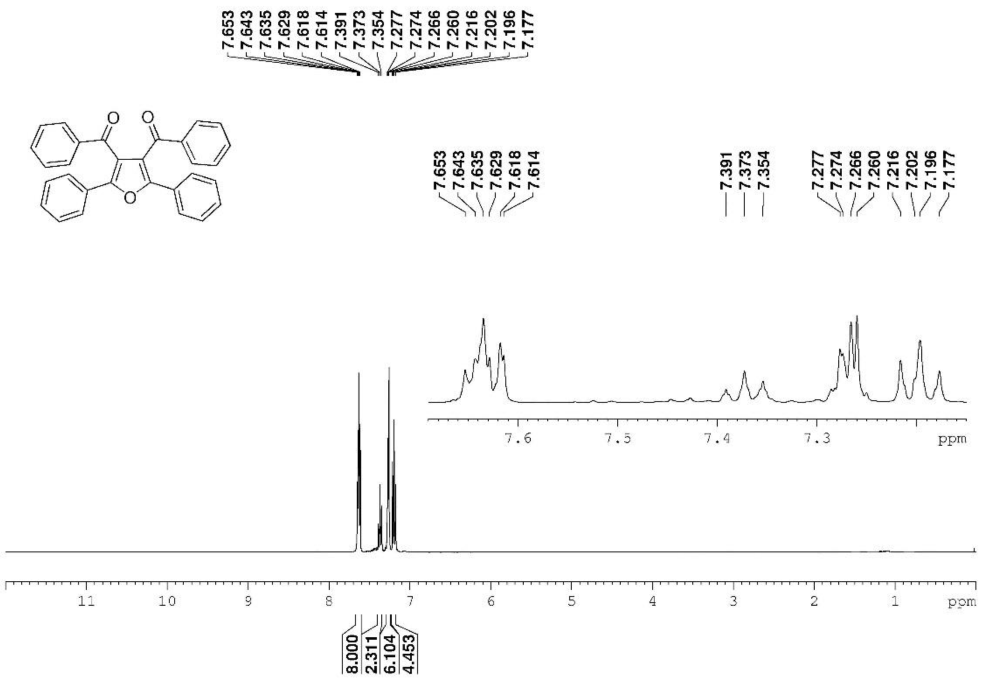

Figure S68. ^1H NMR spectrum of **7b** (400 MHz, $CDCl_3$).

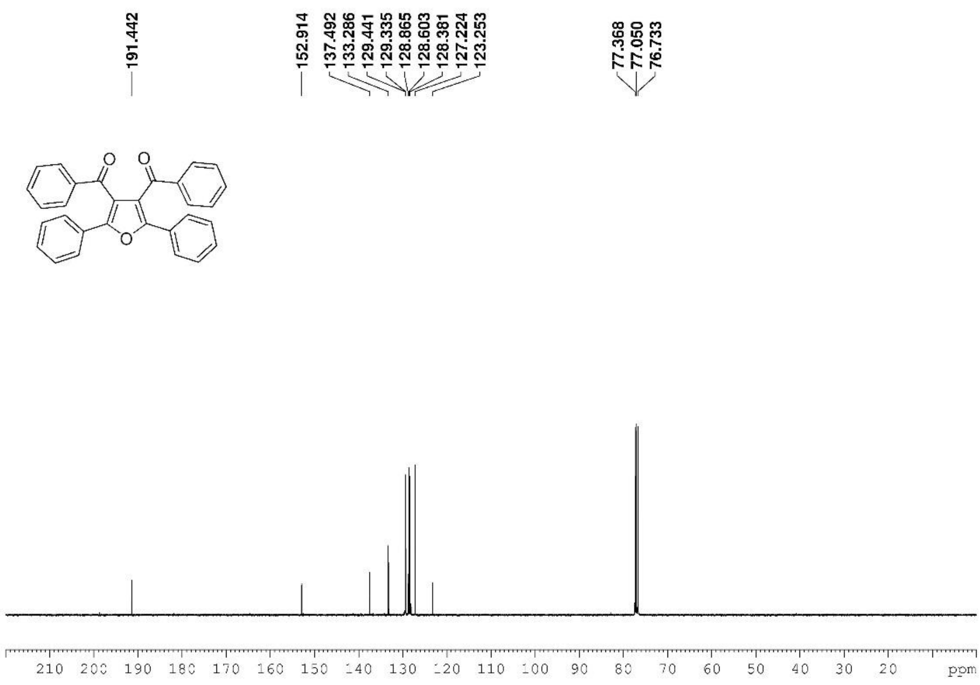

Figure S69. $^{13}C\{^{1}H\}$ NMR spectrum of **7b** (101 MHz, $CDCl_3$).

[2,5-di(4-methoxylphenyl)-3,4-furandiyl)-bis-(4-methoxylphenyl)]-methanone (8b)

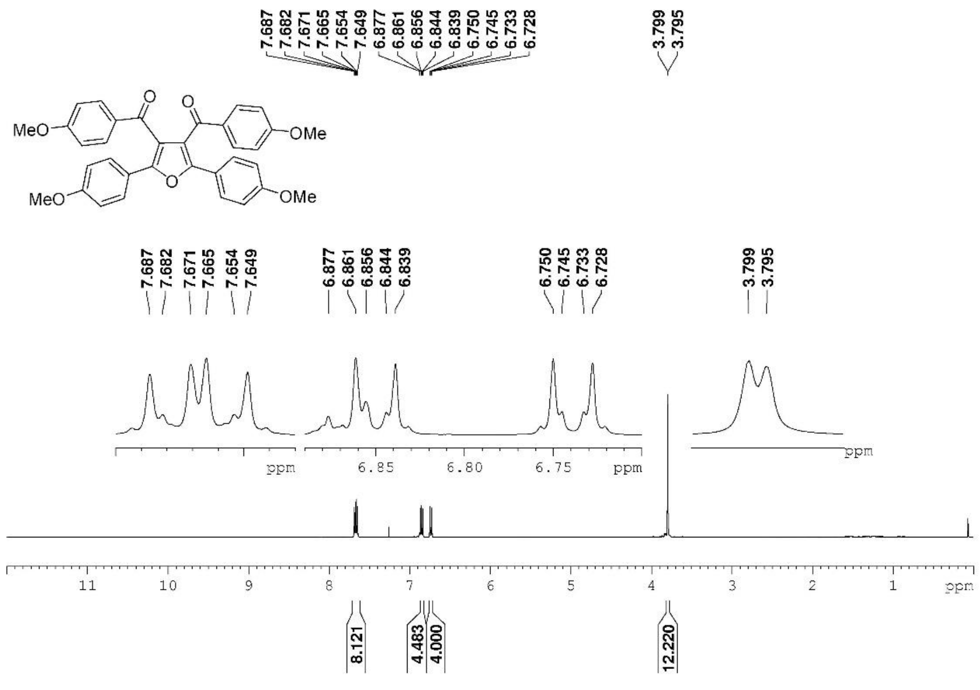

Figure S70. ^{1}H NMR spectrum of **8b** (400 MHz, $CDCl_3$).

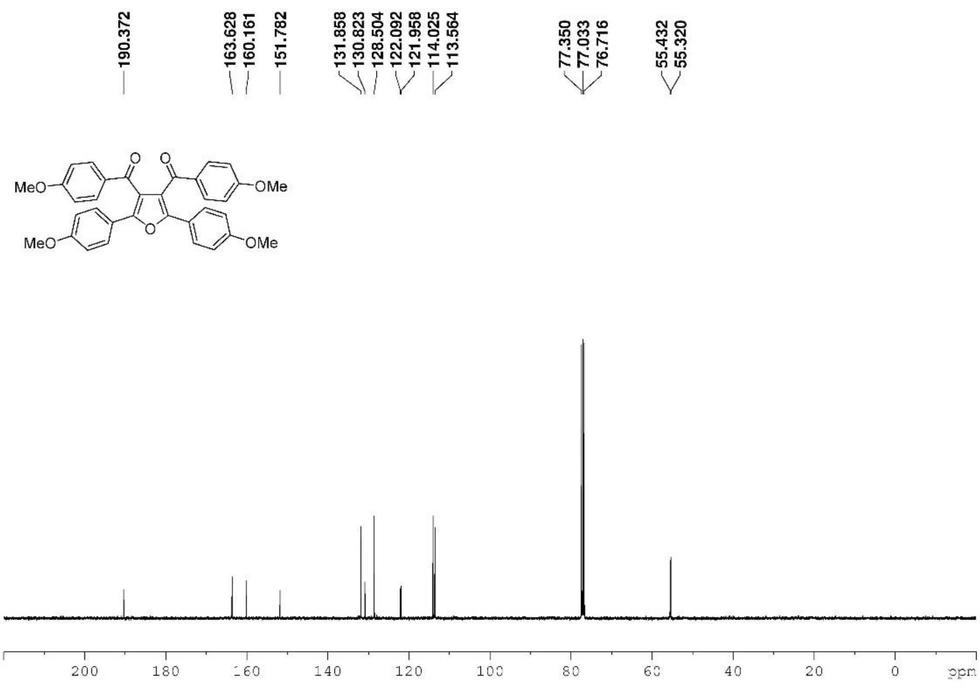

Figure S71. $^{13}C\{^1H\}$ NMR spectrum of **8b** (101 MHz, $CDCl_3$).

[2-(4-*tert*-butyl-phenyl)-3-(4-*tert*-butylbenzoyl)-4-(4-methoxylbenzoyl)-5-(4-methoxylphenyl]-furan (9b)

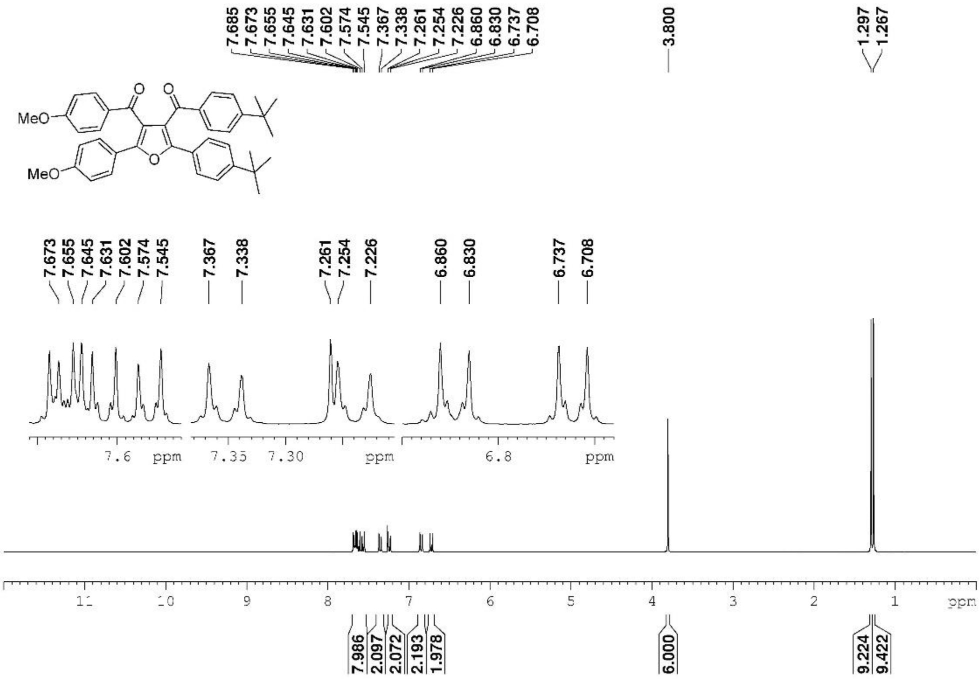

Figure S72. ^1H NMR spectrum of **9b** (400 MHz, $CDCl_3$).

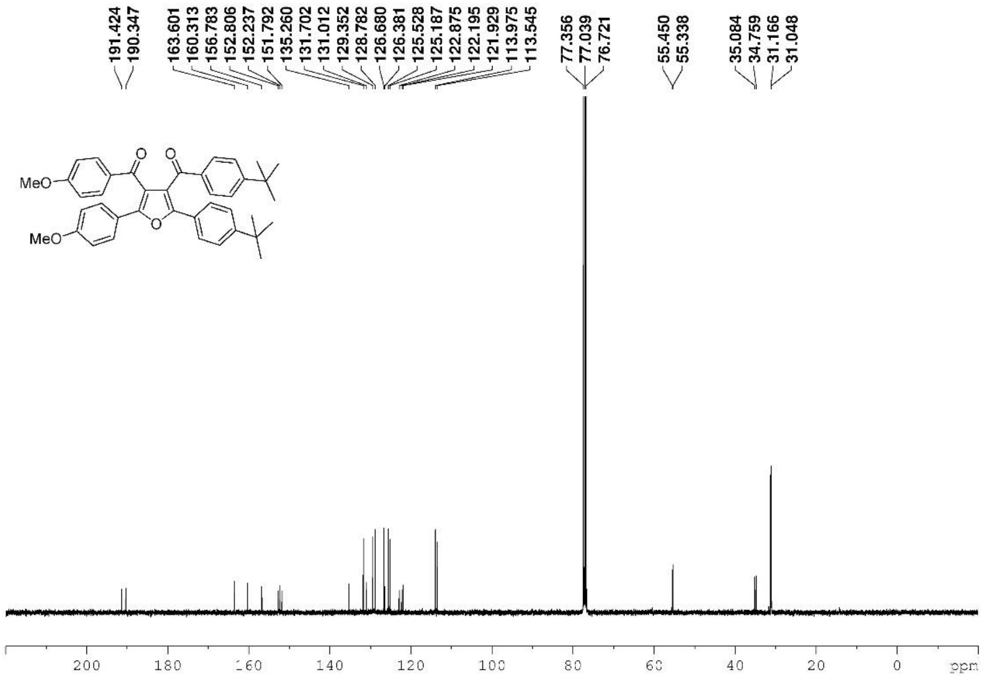

Figure S73. $^{13}C\{^{1}H\}$ NMR spectrum of **9b** (101 MHz, $CDCl_3$).

[2,5-di(4-*tert*-butylphenyl)-3,4-bis(4-methoxylbenzoyl)]-furan (9c)

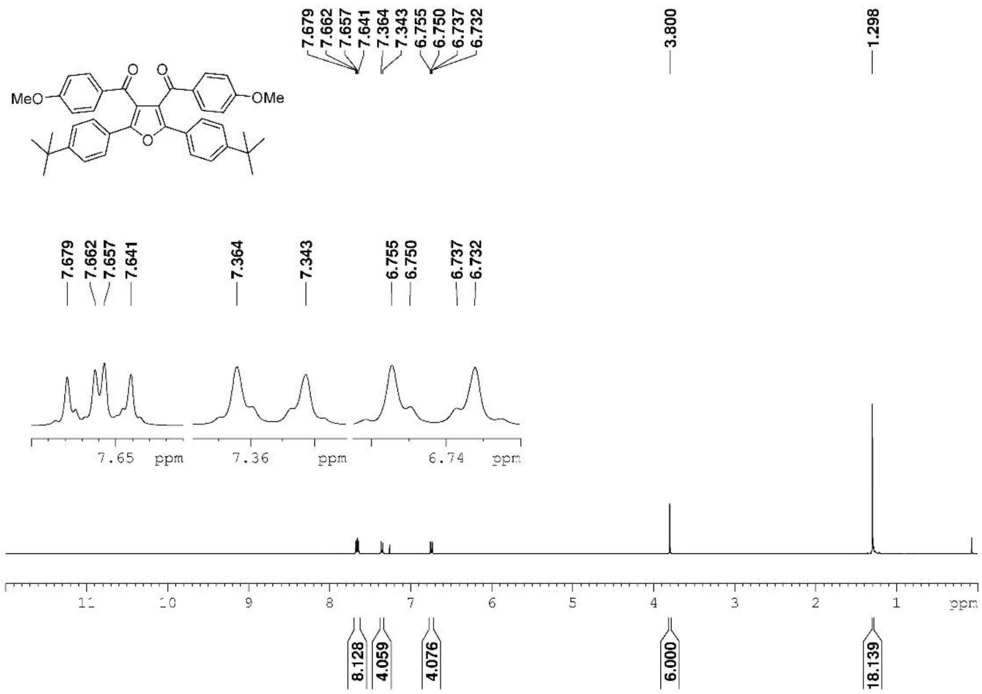

Figure S74. ^{1}H NMR spectrum of **9c** (400 MHz, $CDCl_3$).

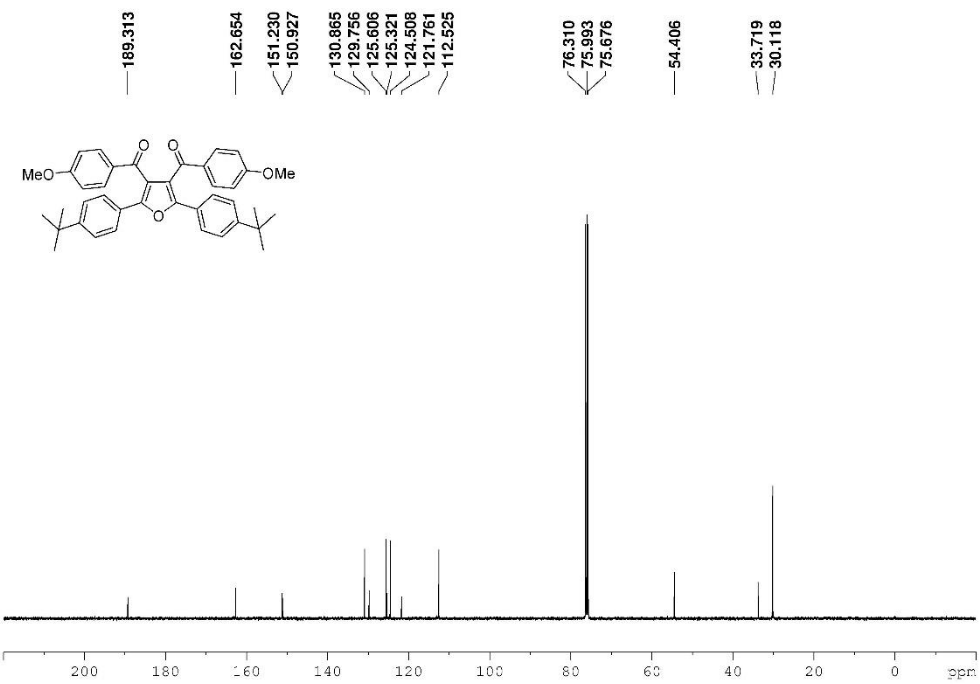

Figure S75. $^{13}C\{^1H\}$ NMR spectrum of **9c** (101 MHz, $CDCl_3$).

[2,5-di(4-methoxylphenyl)-3,4-bis(4-*tert*-butylbenzoyl)]-furan (9d)

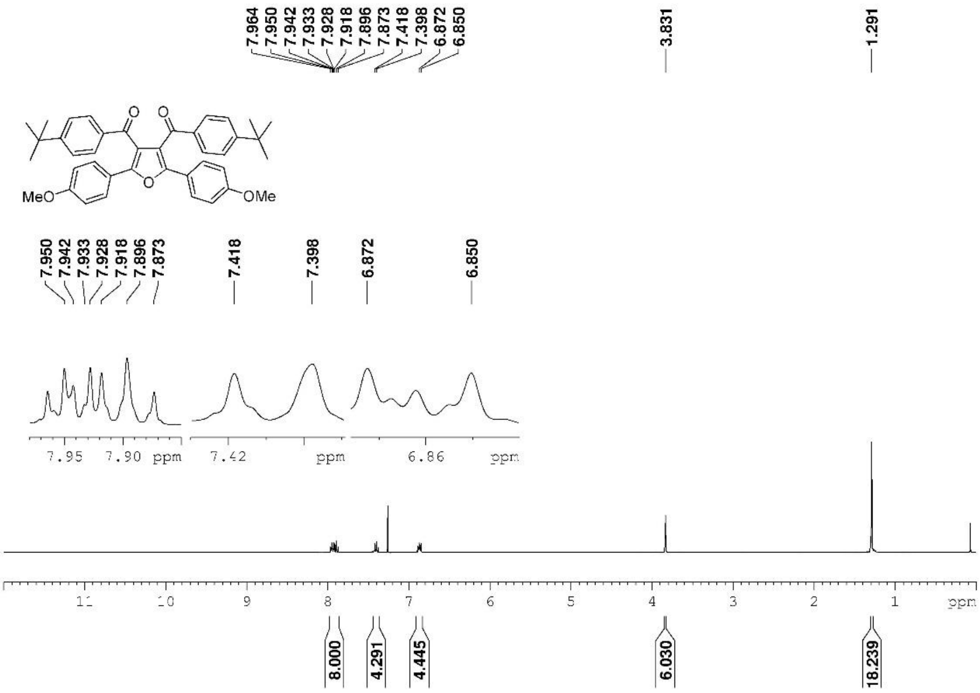

Figure S76. ^1H NMR spectrum of **9d** (400 MHz, $CDCl_3$).

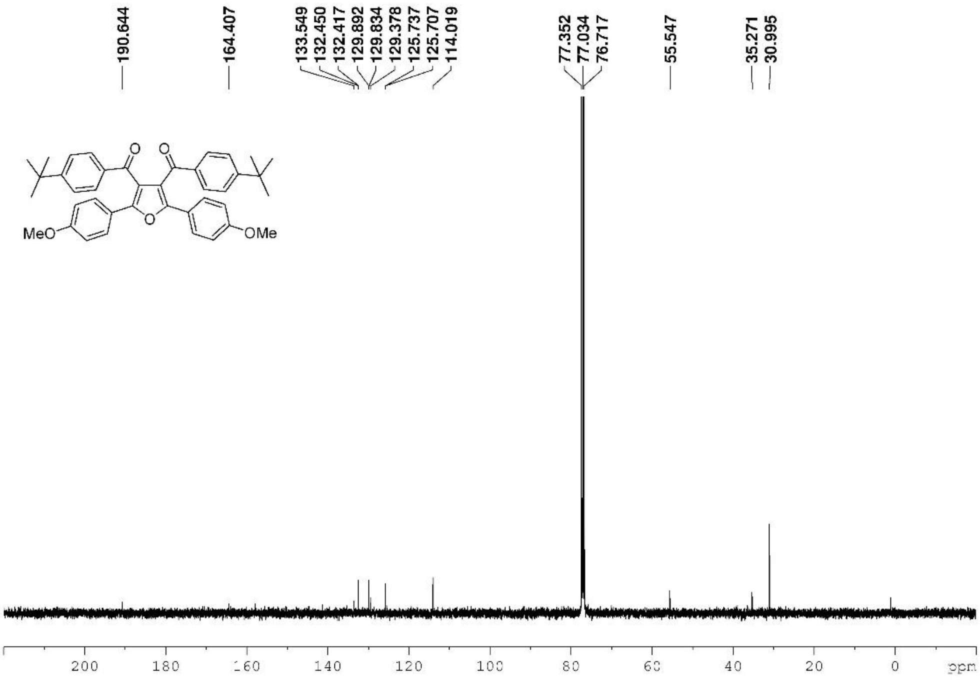

Figure S77. $^{13}C\{^{1}H\}$ NMR spectrum of **9d** (101 MHz, $CDCl_3$).

2,5-dimethyl-3,4-bis(4-phenylbenzoyl)-furan (10c)

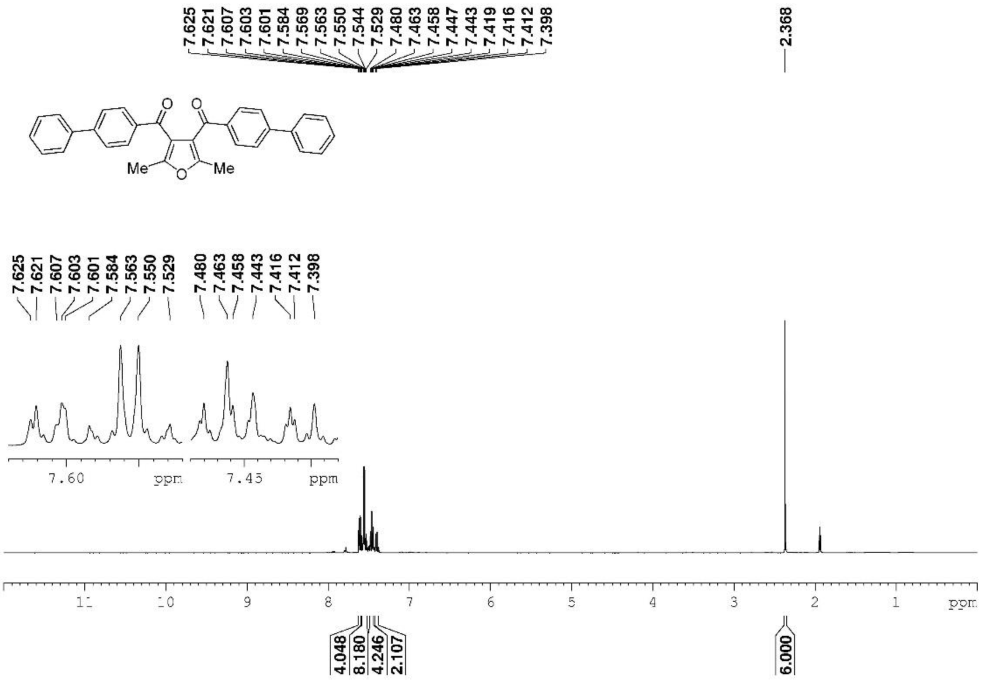

Figure S78. ^{1}H NMR spectrum of **10c** (400 MHz, CD_3CN).

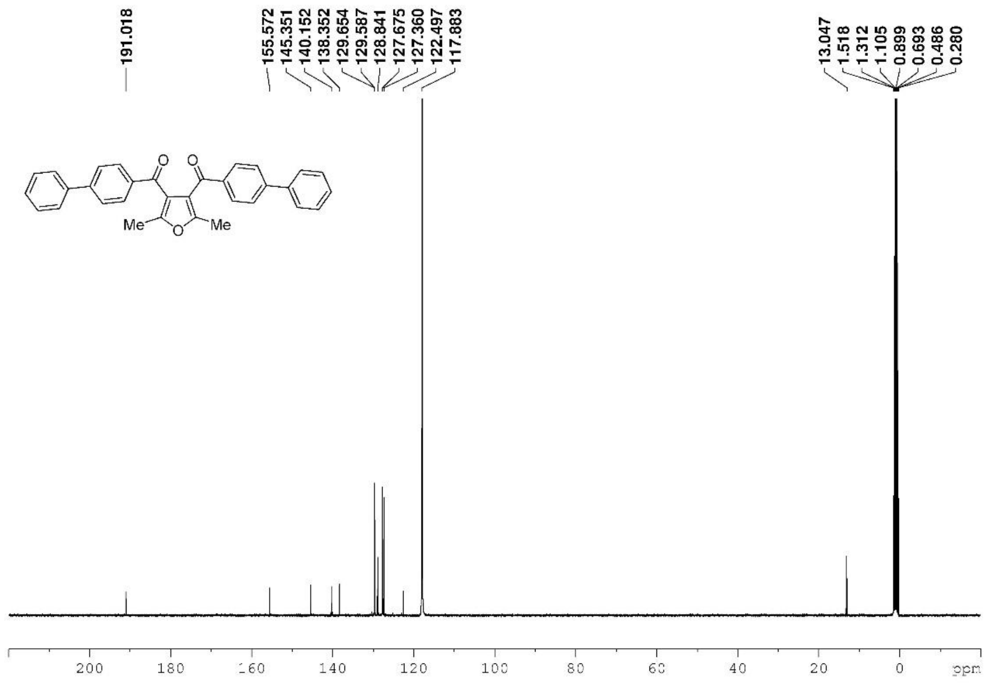

Figure S79. $^{13}C\{^{1}H\}$ NMR spectrum of **10c** (101 MHz, CD_3CN).

1-[4-(4-phenoxybenzoyl)-2-methyl-5-(4-phenoxyphenyl)-3-furanyl]-ethanone (11b)

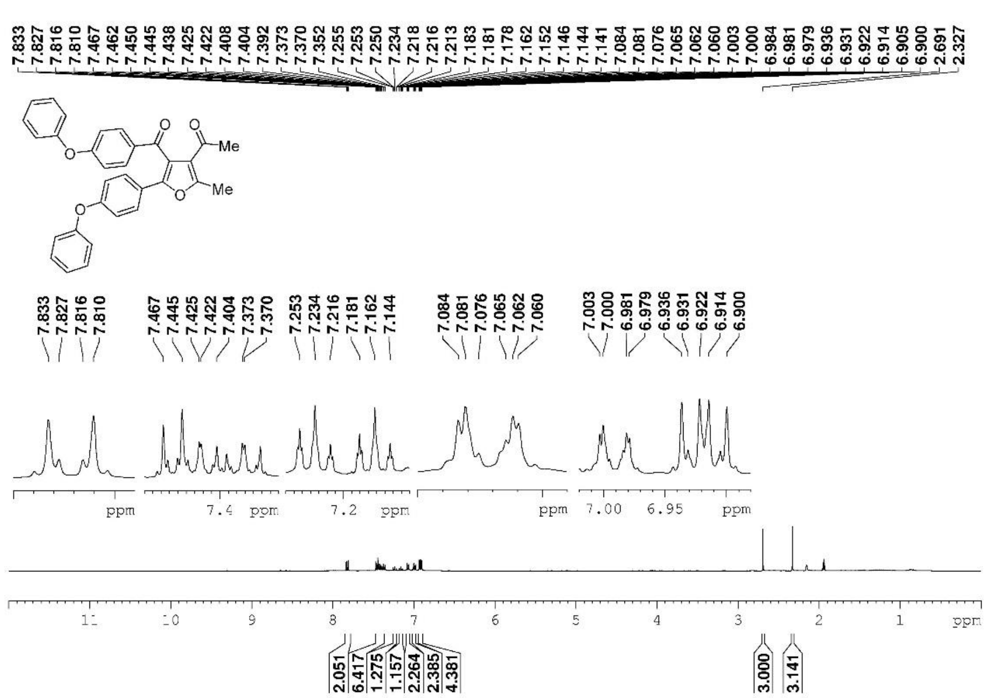

Figure S80. ^{1}H NMR spectrum of **11b** (400 MHz, CD_3CN).

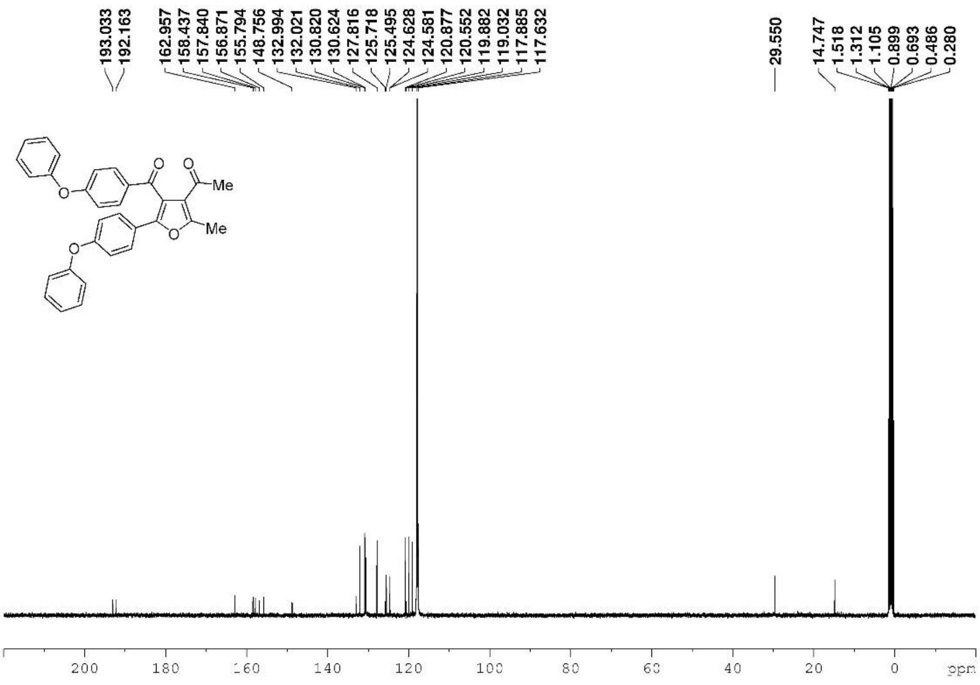

Figure S81. $^{13}C\{^{1}H\}$ NMR spectrum of **11b** (101 MHz, CD_3CN).

2,5-dimethyl-3,4-bis(4-phenoxybenzoyl)-furan (11c)

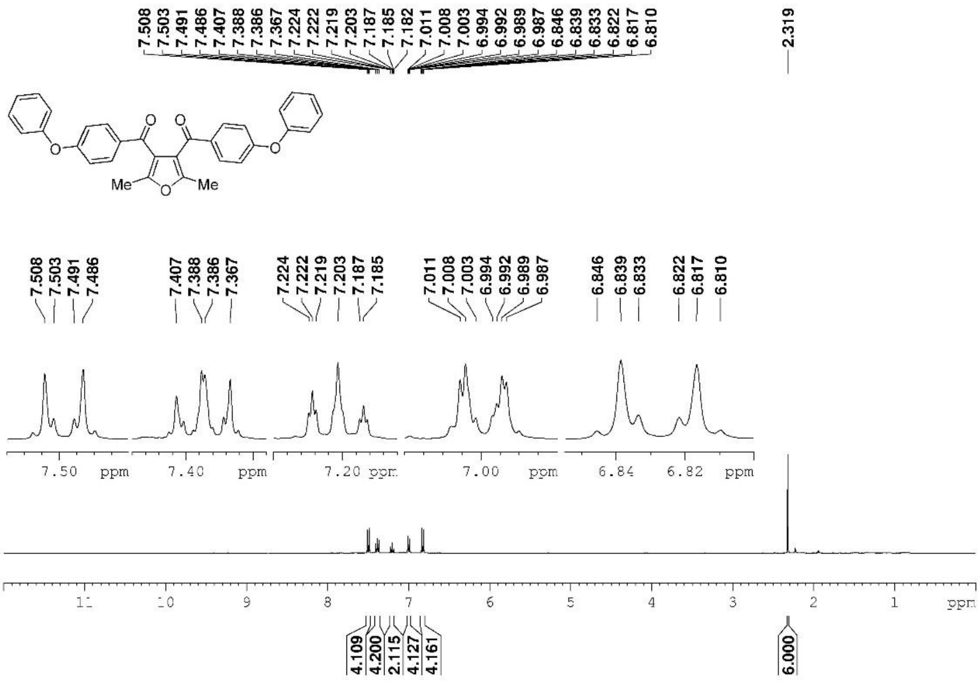

Figure S82. ^{1}H NMR spectrum of **11c** (400 MHz, CD_3CN).

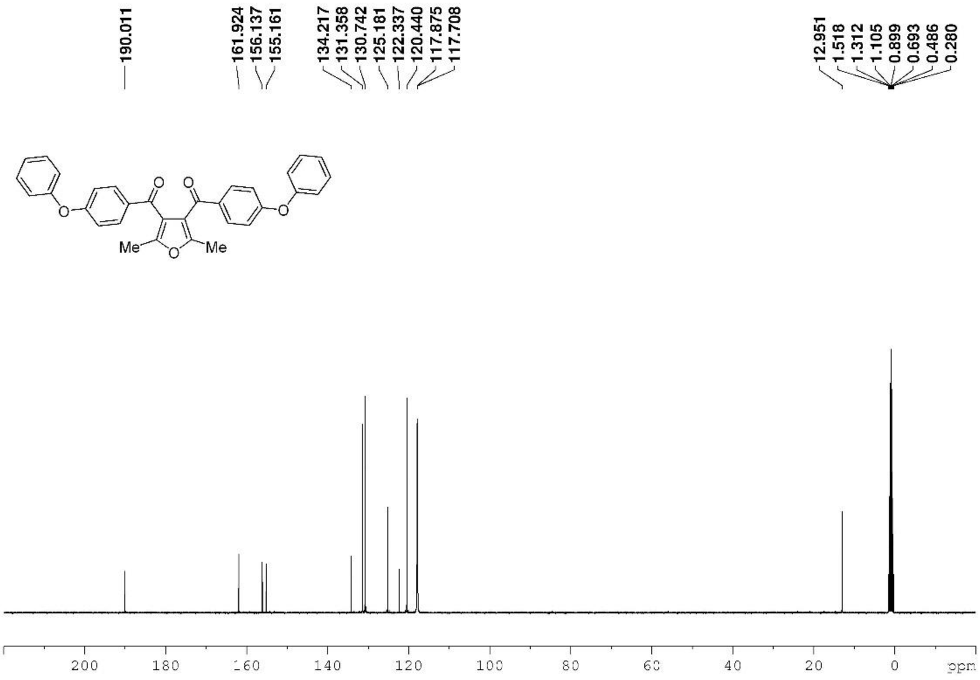

Figure S83. $^{13}C\{^{1}H\}$ NMR spectrum of **11c** (101 MHz, CD_3CN).

[2-methyl-3-(methyl-carbonyl)-4-(naphthalene-2-carbonyl)-5-(2-naphthalene)]-furan (12b)

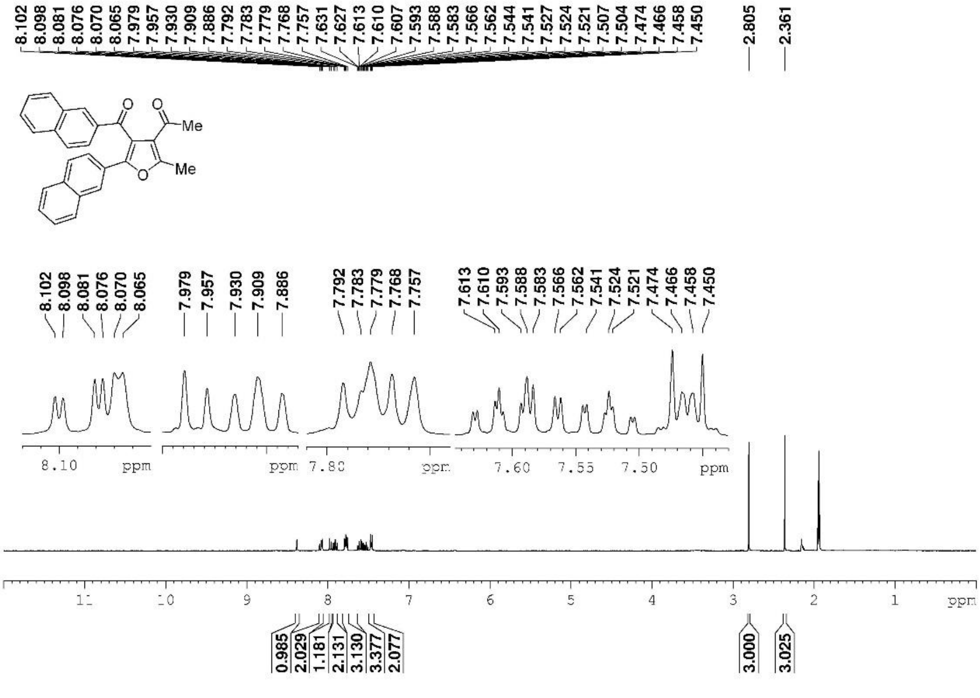

Figure S84. ^{1}H NMR spectrum of **12b** (400 MHz, CD_3CN).

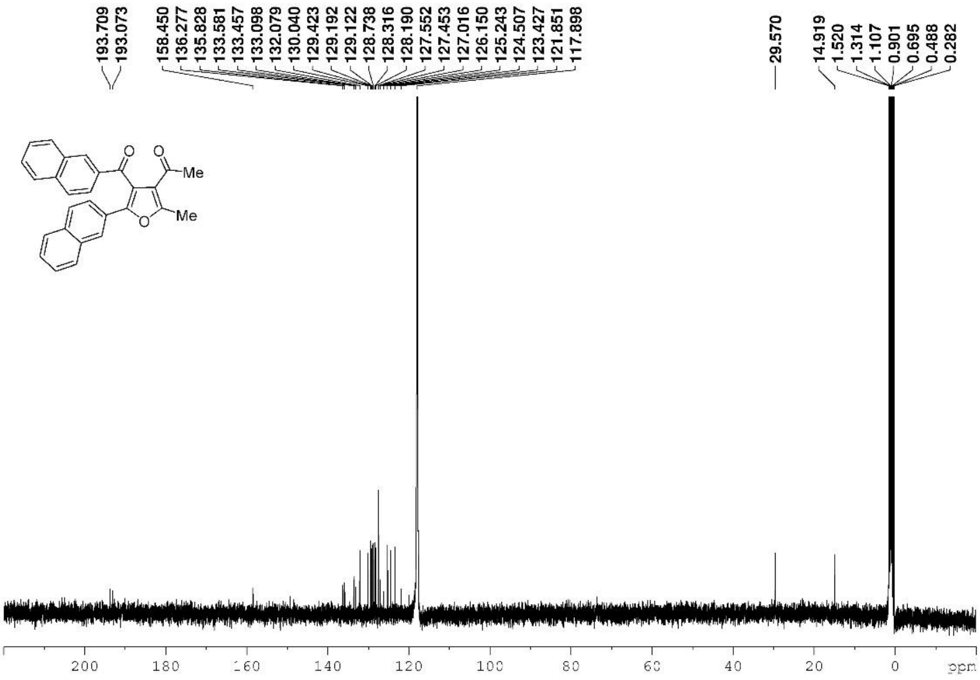

Figure S85. $^{13}C\{^{1}H\}$ NMR spectrum of **12b** (101 MHz, CD_3CN).

2,5-dimethyl-3,4-bis(naphthalene-2-carbonyl)-furan (12c)

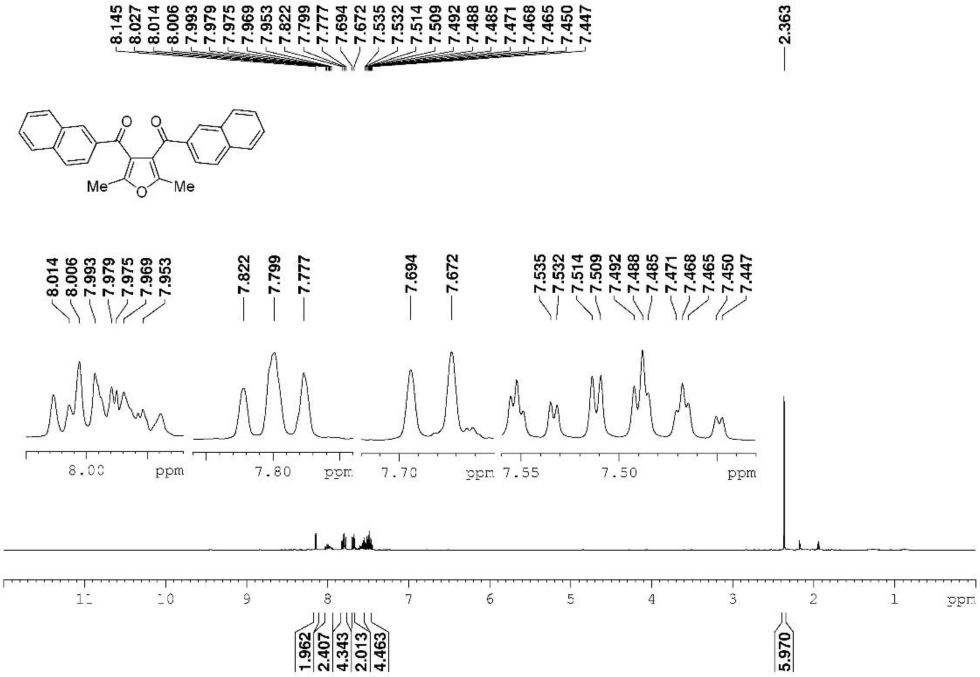

Figure S86. ^{1}H NMR spectrum of **12c** (400 MHz, CD_3CN).

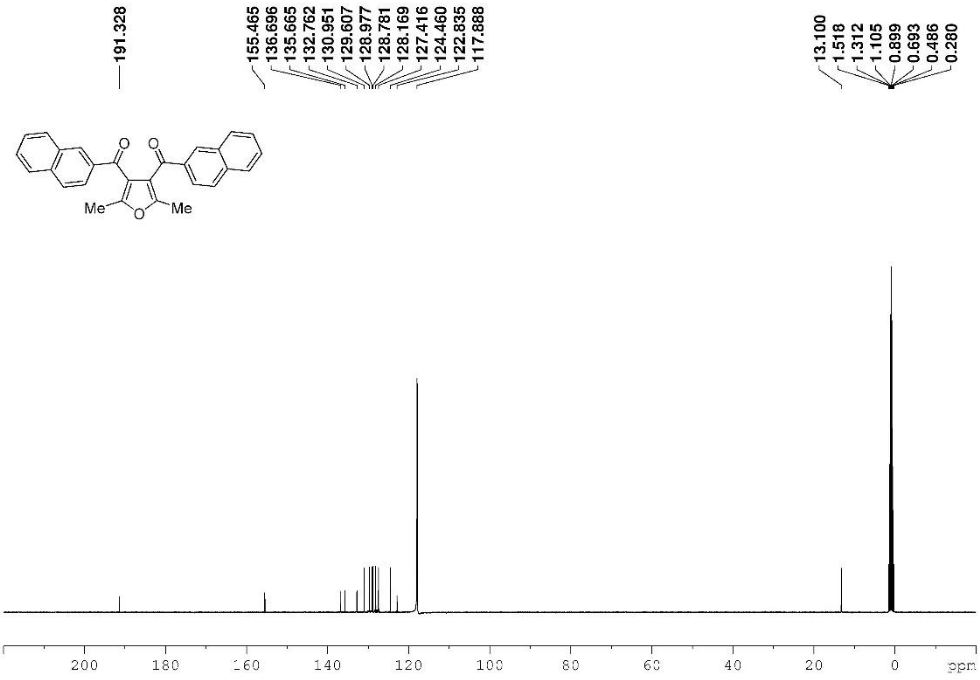

Figure S87. $^{13}C\{^{1}H\}$ NMR spectrum of **12c** (101 MHz, CD_3CN).

[2-methyl-3-(methyl-carbonyl)-4-(2,3-dihydro-1,4-benzodioxin-6-yl-methanone)-5-(2,3-dihydro -1,4-benzodioxin)]-furan (13b)

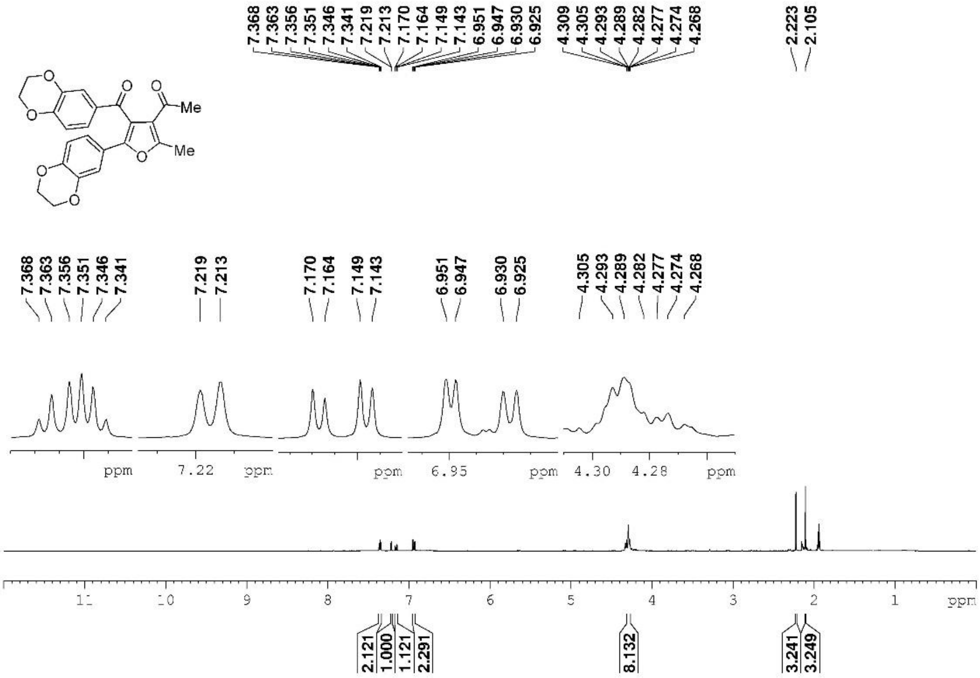

Figure S88. ^{1}H NMR spectrum of **13b** (400 MHz, CD_3CN).

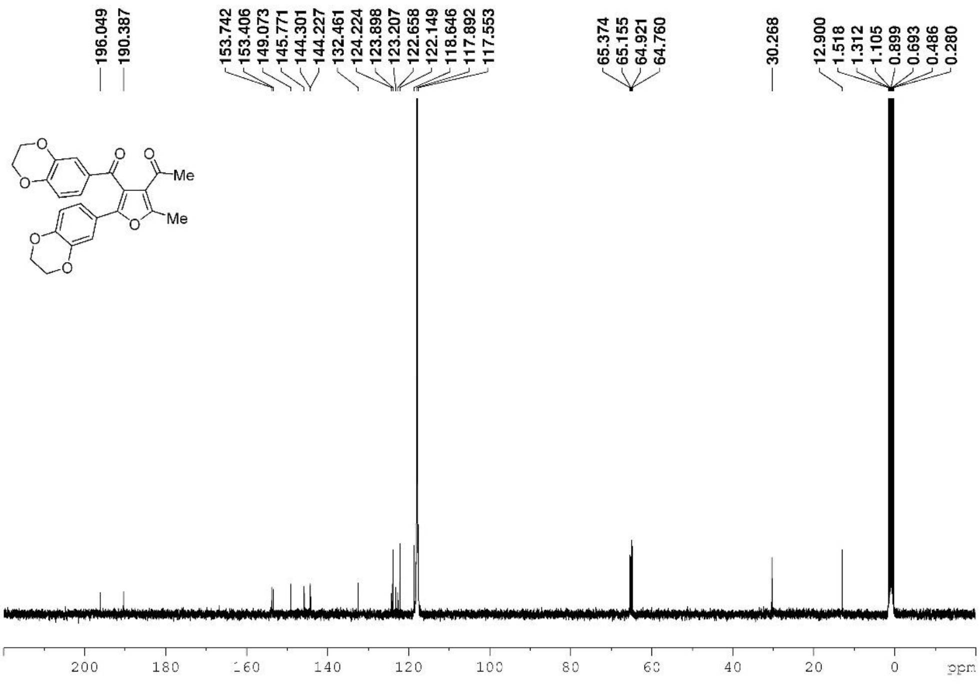

Figure S89. $^{13}C\{^{1}H\}$ NMR spectrum of **13b** (101 MHz, CD_3CN).

2,5-dimethyl-3,4-bis(2,3-dihydro-1,4-benzodioxin-6-yl-methanone)-furan (13c)

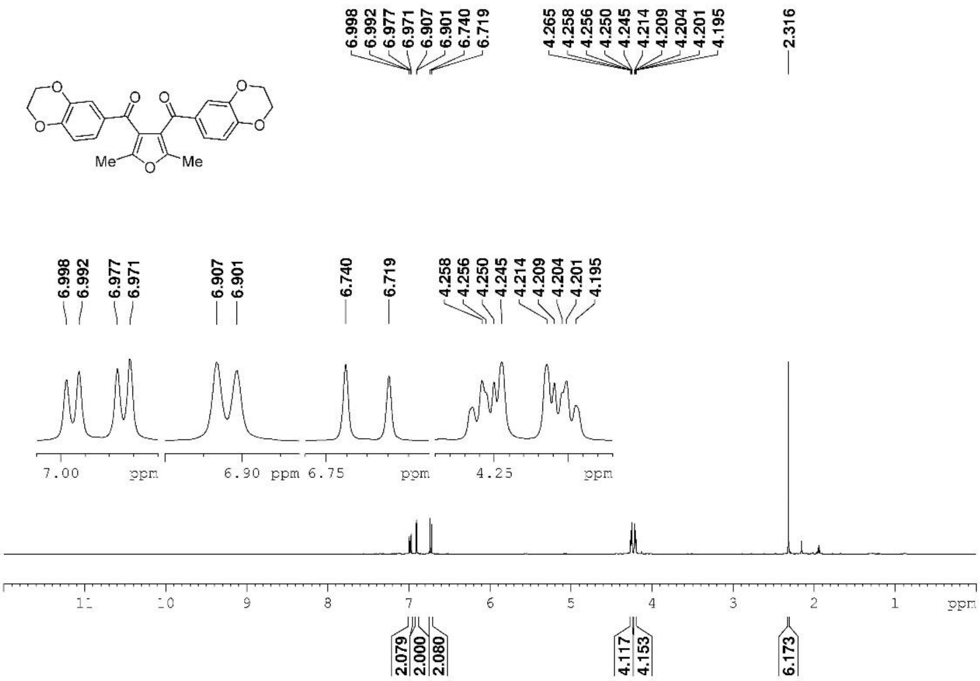

Figure S90. ^{1}H NMR spectrum of **13c** (400 MHz, CD_3CN).

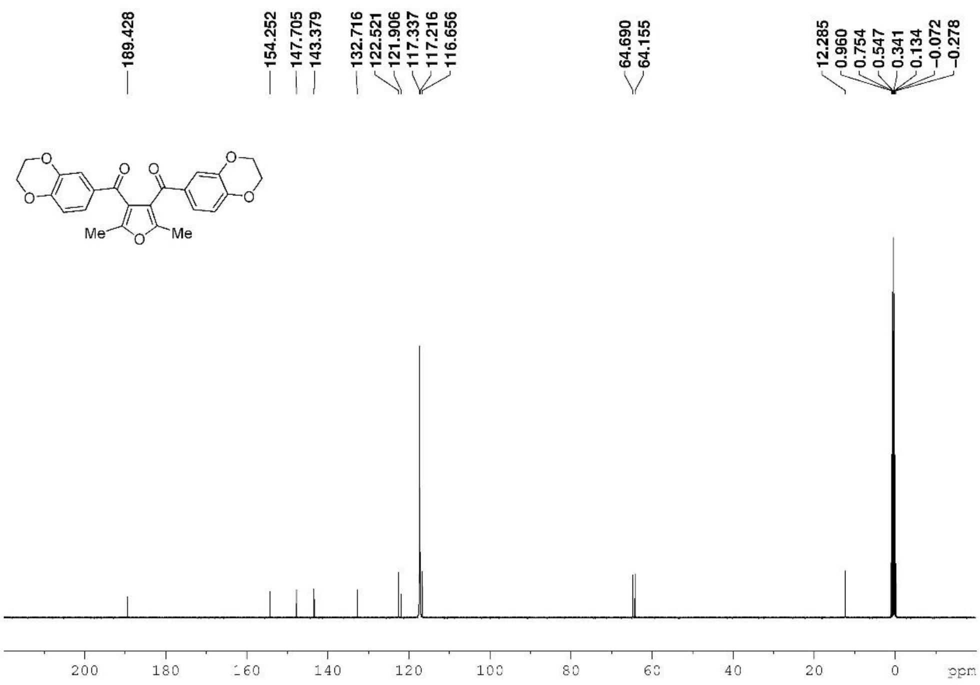

Figure S91. $^{13}C\{^1H\}$ NMR spectrum of **13c** (101 MHz, CD_3CN).

[2-methyl-3-(methyl-carbonyl)-4-(furan-2-carbonyl)-5-furanyl]-furan (14b)

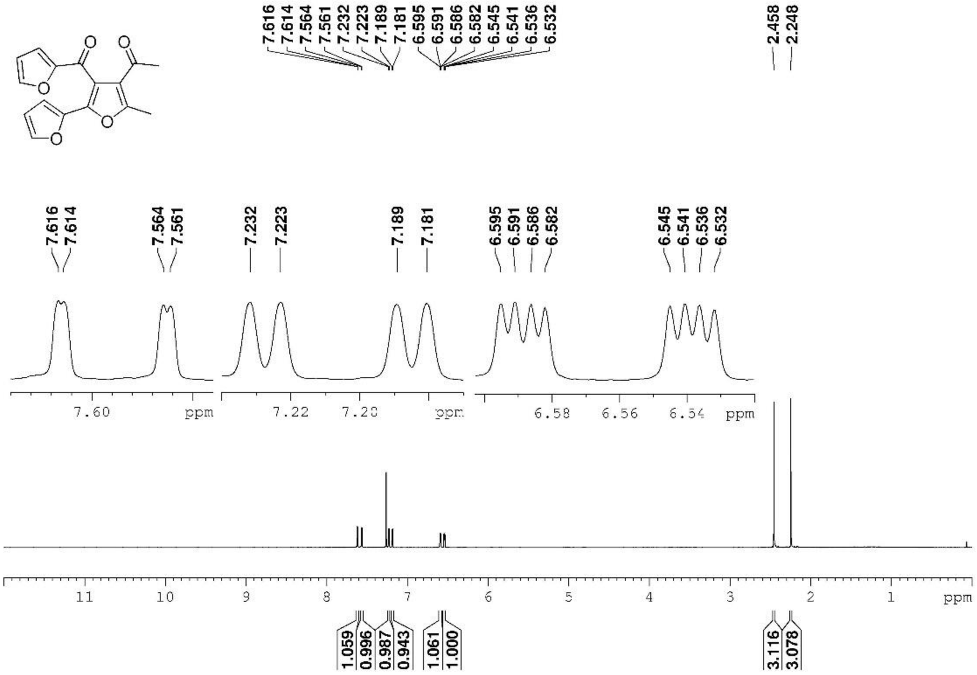

Figure S92. ^1H NMR spectrum of **14b** (400 MHz, $CDCl_3$).

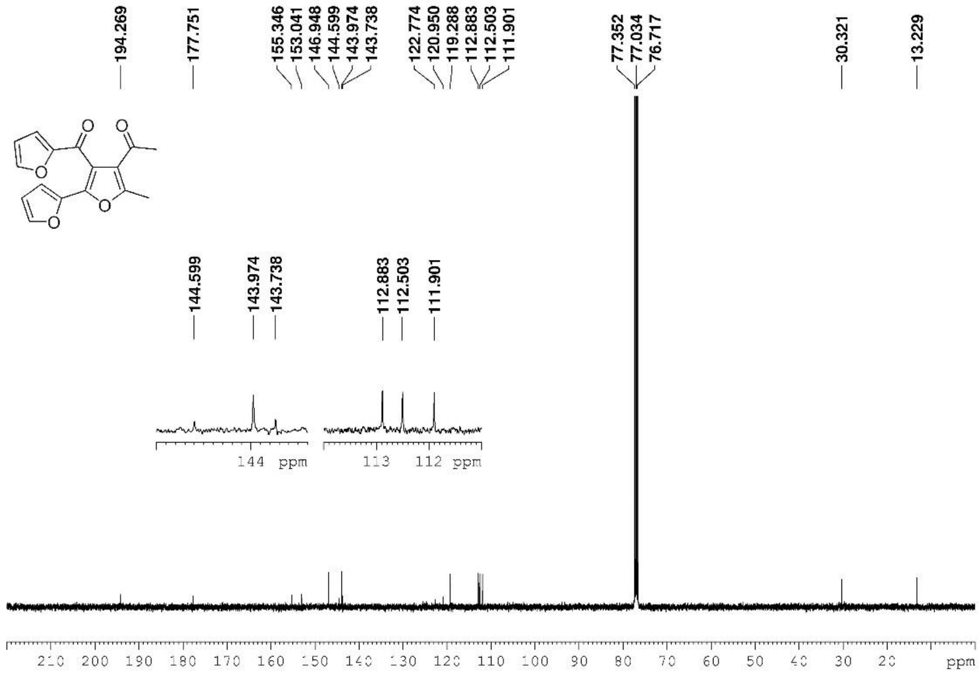

Figure S93. $^{13}C\{^{1}H\}$ NMR spectrum of **13c** (101 MHz, $CDCl_3$).

2,5-dimethyl-3,4-bis(furan-2-carbonyl)-furan (14c)

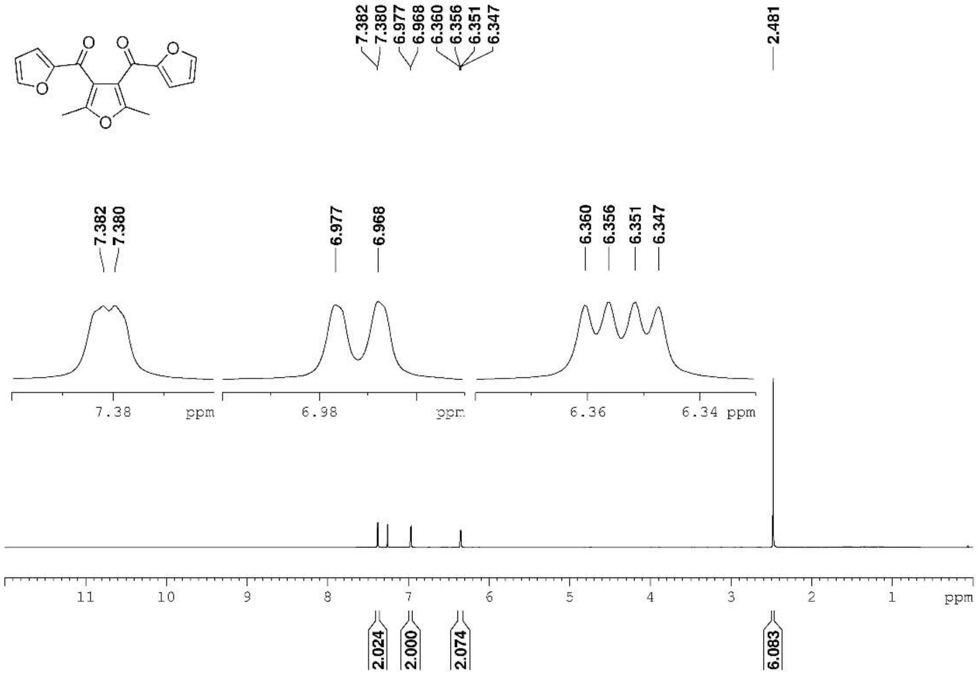

Figure S94. ^{1}H NMR spectrum of **14c** (400 MHz, $CDCl_3$).

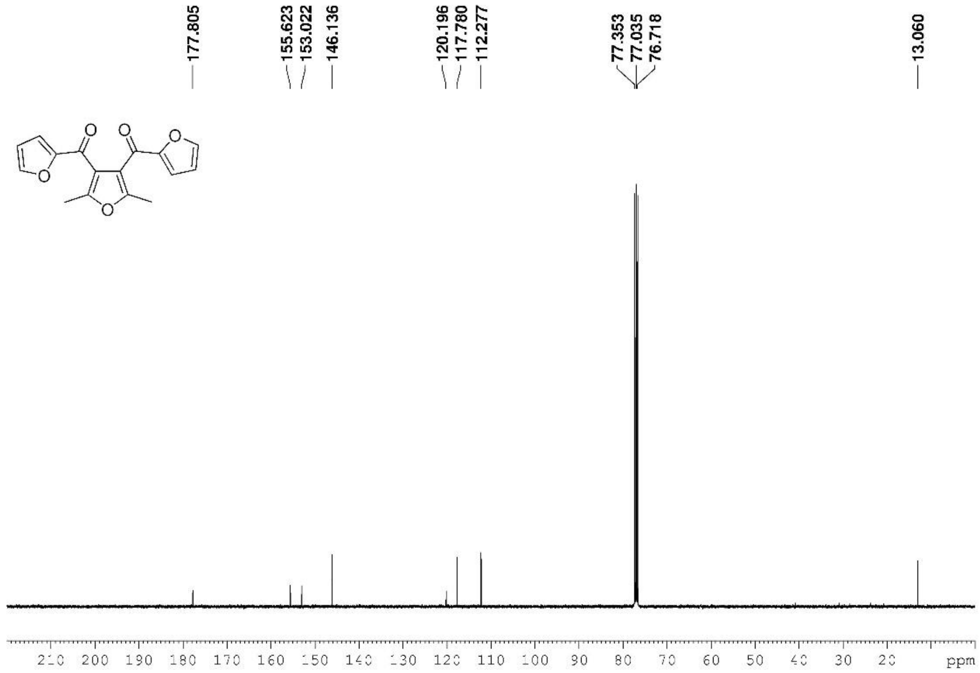

Figure S95. $^{13}C\{^{1}H\}$ NMR spectrum of **14c** (101 MHz, $CDCl_3$).

[2-methyl-3-(methyl-carbonyl)-4-(thiophene-2-carbonyl)-5-thienyl]-furan (15b)

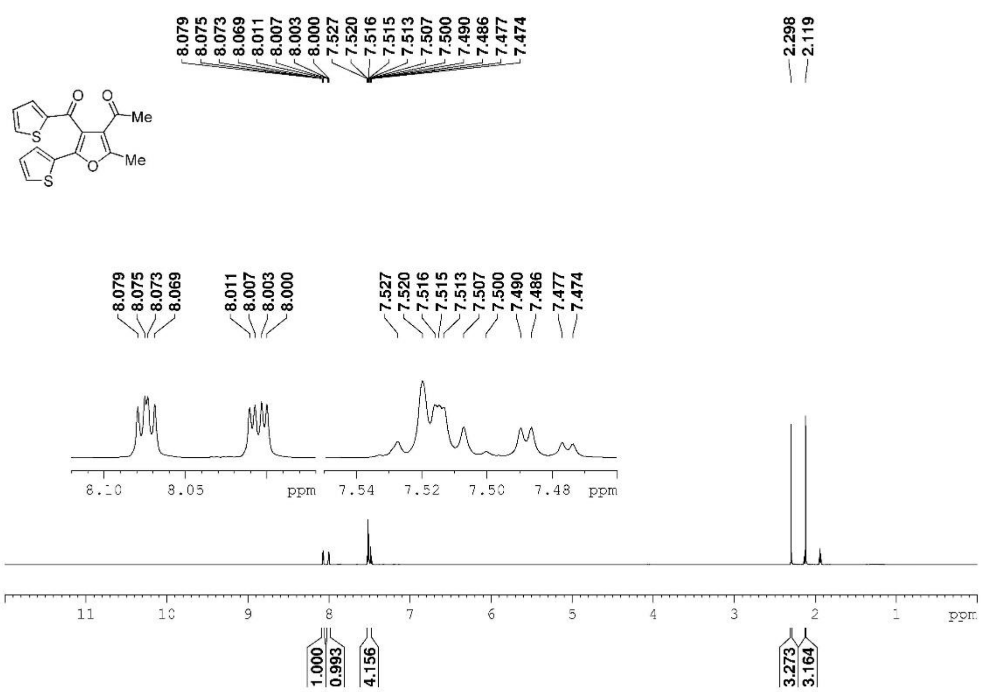

Figure S96. ^{1}H NMR spectrum of **15b** (400 MHz, CD_3CN).

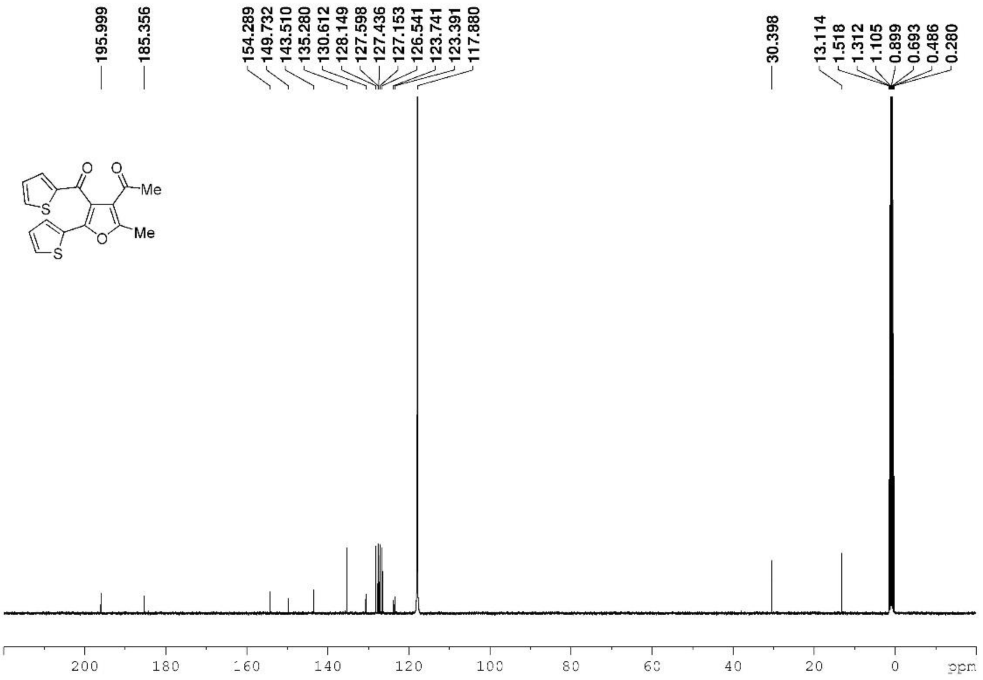

Figure S97. $^{13}C\{^{1}H\}$ NMR spectrum of **15b** (101 MHz, CD_3CN).

2,5-dimethyl-3,4-bis(thiophene-2-carbonyl)-furan (15c)

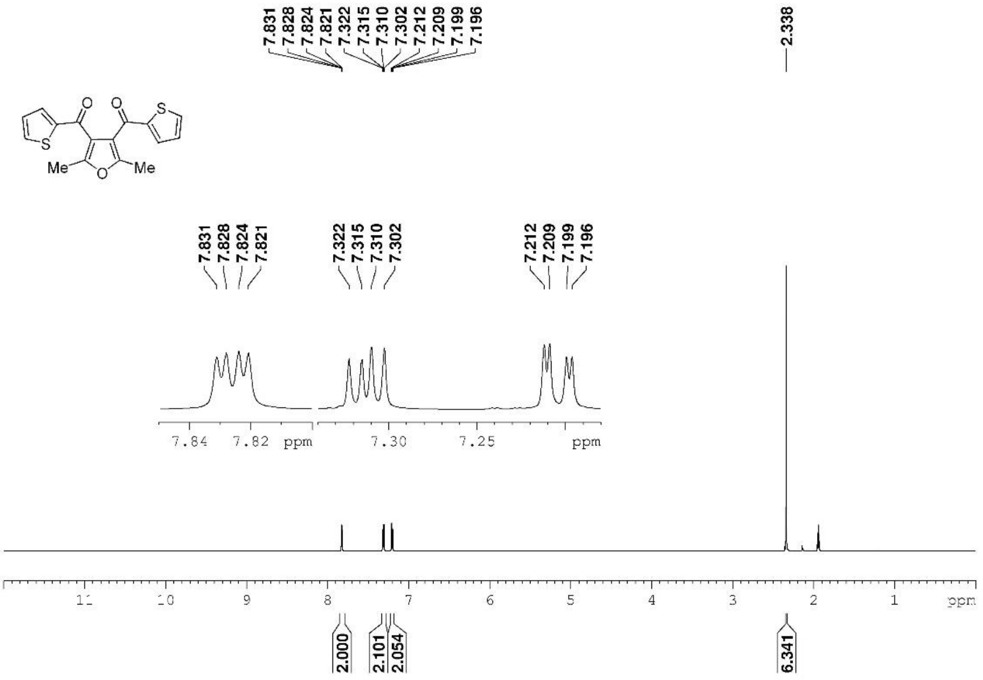

Figure S98. ^{1}H NMR spectrum of **15c** (400 MHz, CD_3CN).

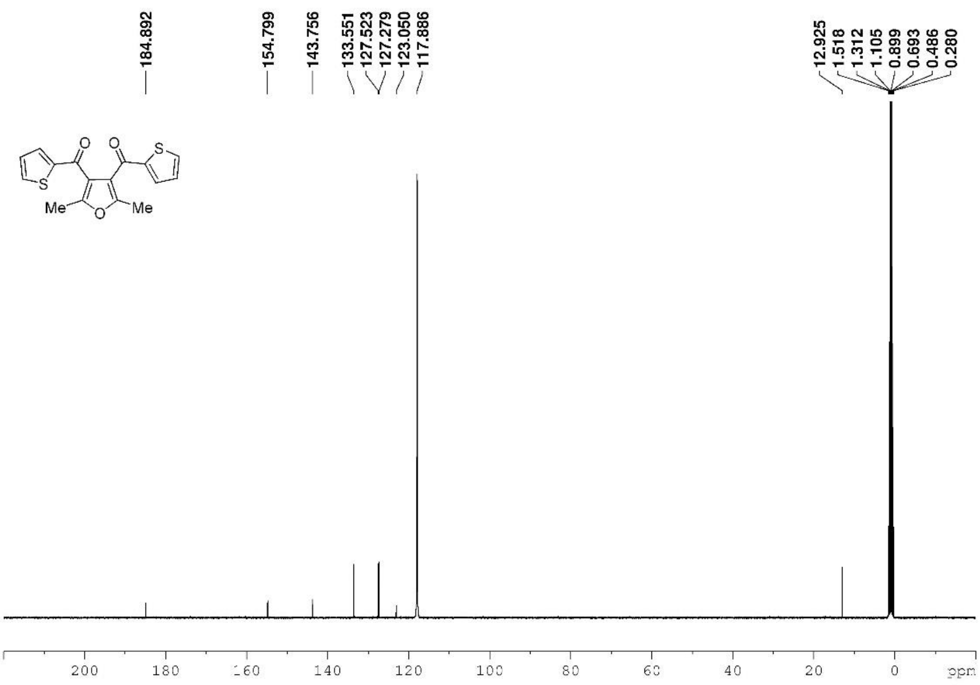

Figure S99. $^{13}C\{^1H\}$ NMR spectrum of **15c** (101 MHz, CD_3CN).

1-[2-ethoxyl-3-carboethoxy-2,5-dihydro-5-phenyl-3-furanyl]-benzophenone (16b)

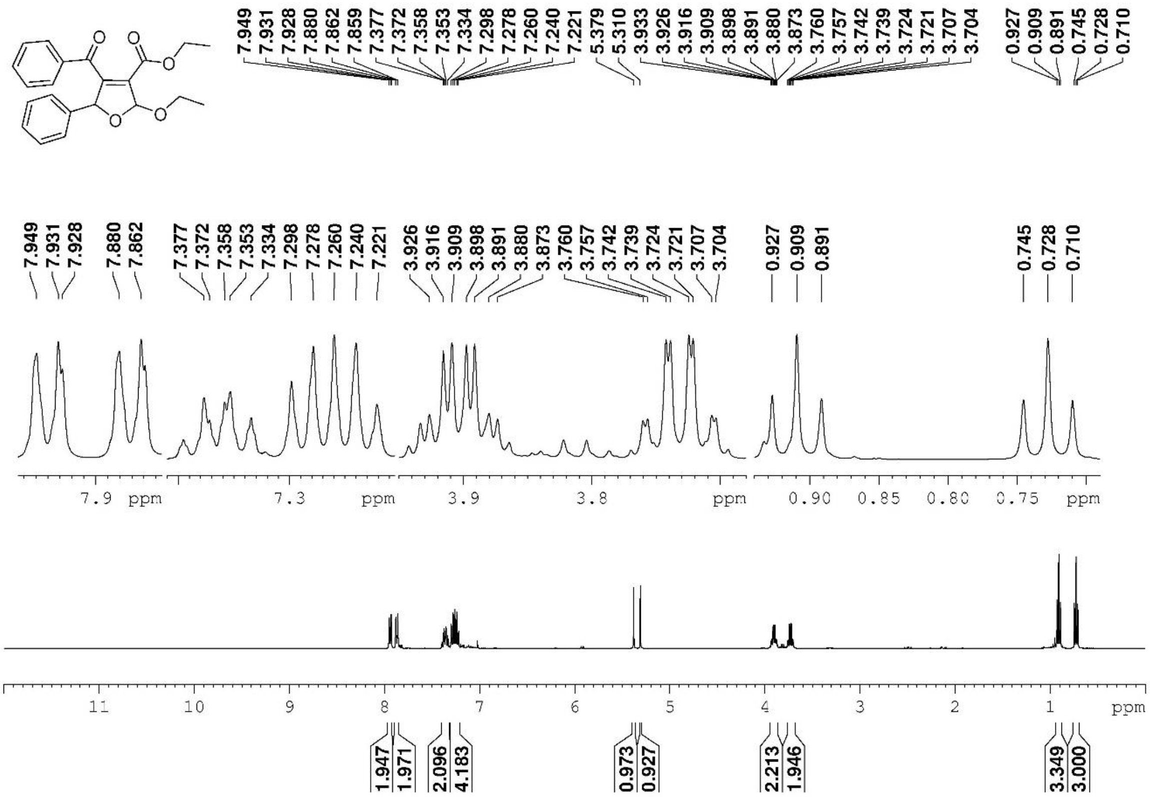

Figure S100. ^1H NMR spectrum of **16b** (400 MHz, $CDCl_3$).

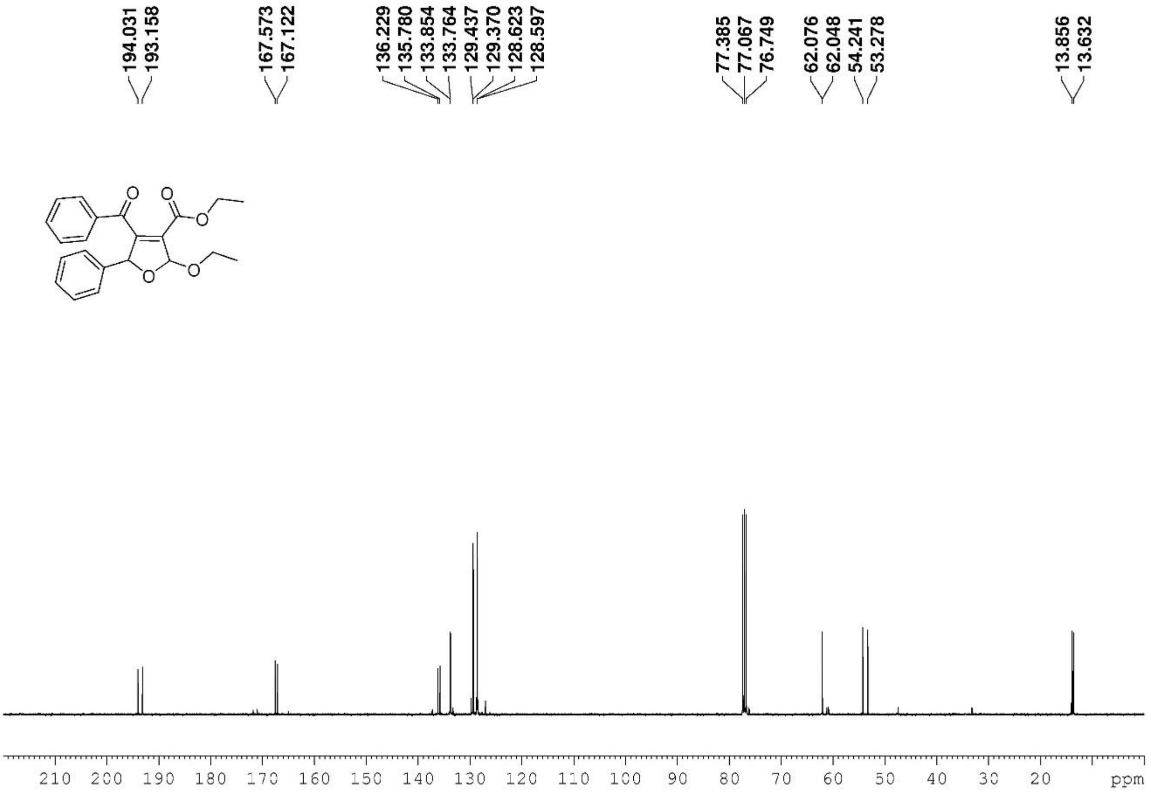

Figure S101. $^{13}C\{^{1}H\}$ NMR spectrum of **16b** (101 MHz, $CDCl_3$).

1-[2,5-di(ethoxyl)-3-benzoyl-2,3-dihydro-3-furanyl]-benzophenone (16c)

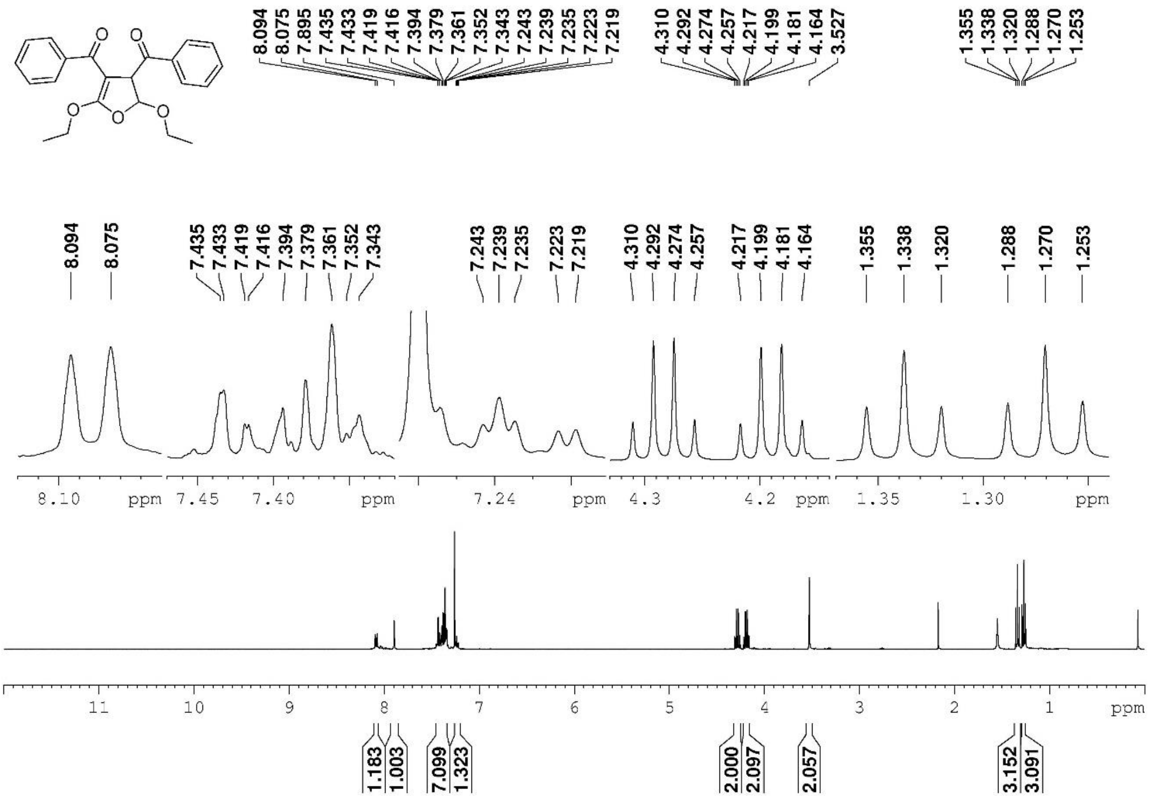

Figure S102. ^{1}H NMR spectrum of **16c** (400 MHz, $CDCl_3$).

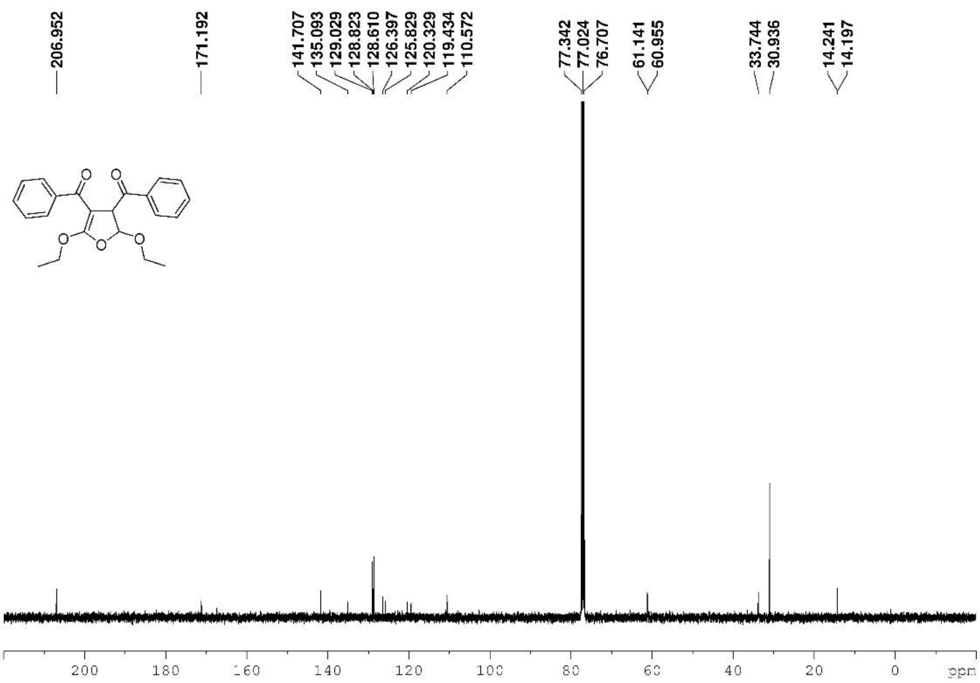

Figure S103. $^{13}C\{^{1}H\}$ NMR spectrum of **16c** (101 MHz, $CDCl_3$).

1-[2-ethoxyl-3-carboethoxy-2,5-dihydro-5-(4-fluorophenyl)-3-furanyl]-4-fluorobenzophenone (17b)

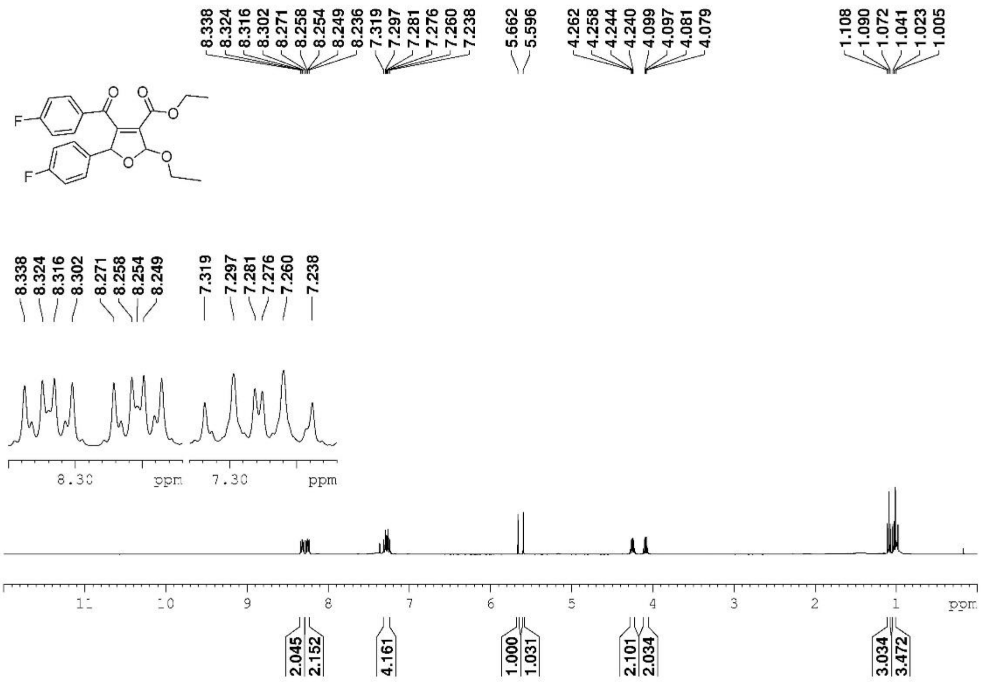

Figure S104. ^{1}H NMR spectrum of **17b** (400 MHz, $CDCl_3$).

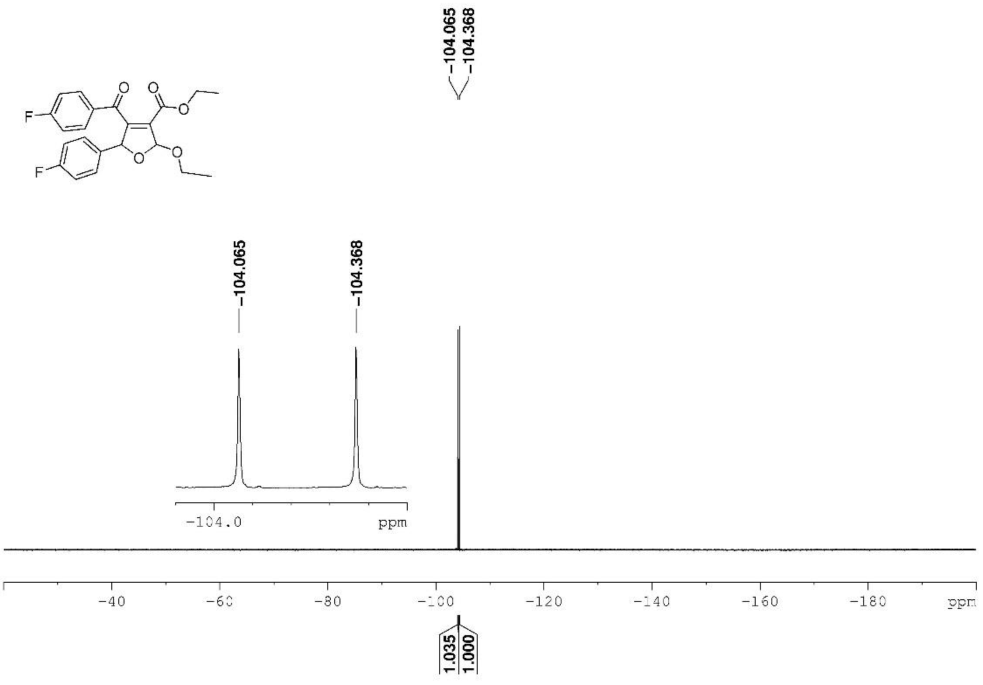

Figure S105. $^{19}F\{^1H\}$ NMR spectrum of **17b** (376 MHz, $CDCl_3$).

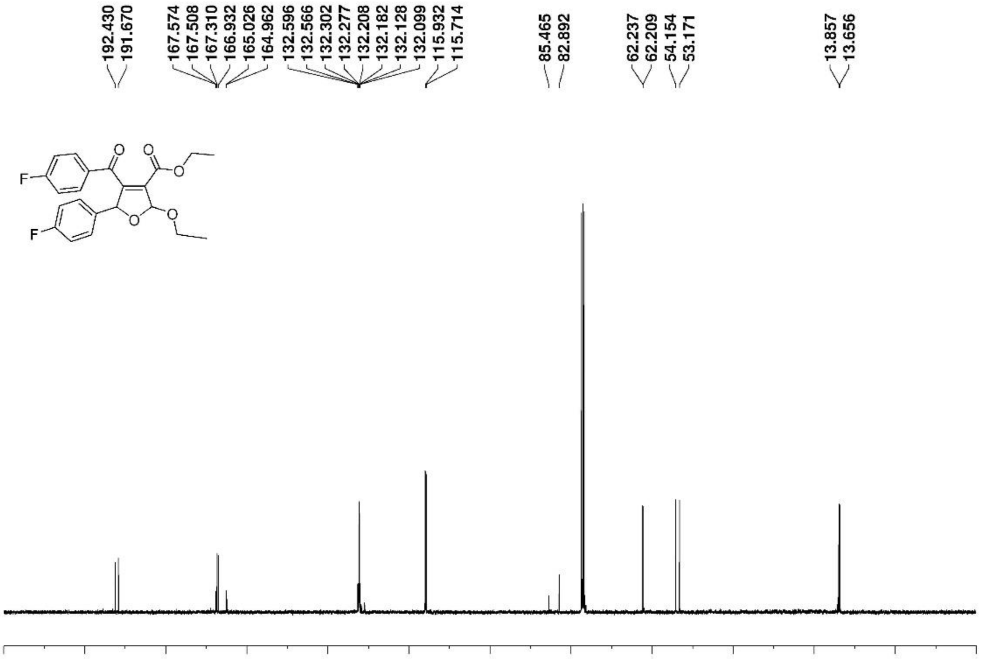

Figure S106. $^{13}C\{^1H\}$ NMR spectrum of **17b** (101 MHz, $CDCl_3$).

1-[2,5-di(ethoxyl)-3-(4-fluorobenzoyl)-2,5-dihydro-3-furanyl]-4-fluorobenzophenone (17c)

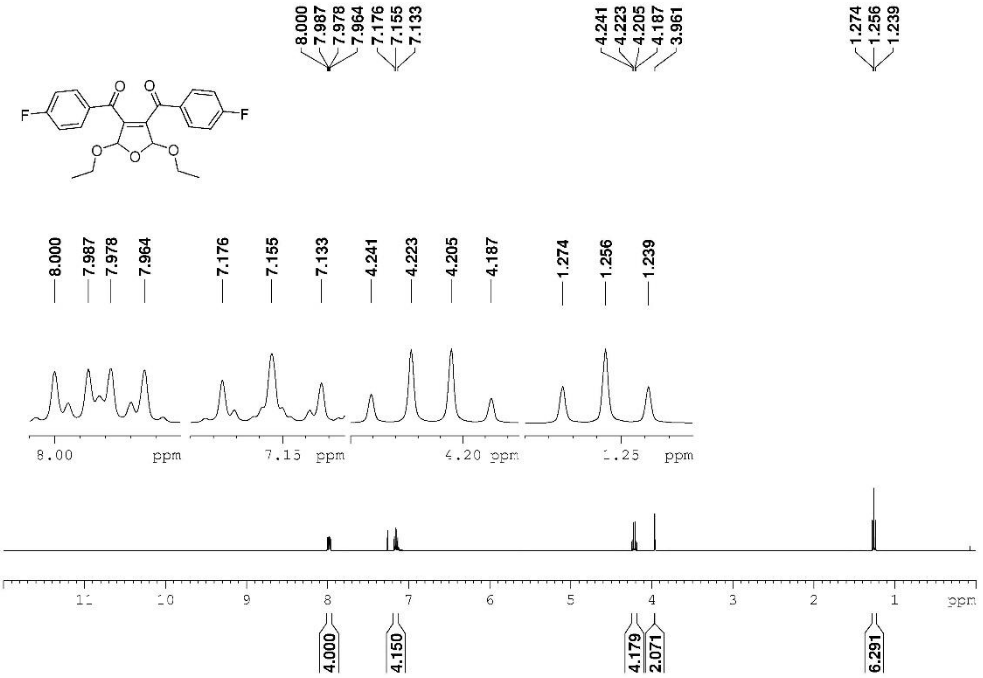

Figure S107. ^{1}H NMR spectrum of **17c** (400 MHz, $CDCl_3$).

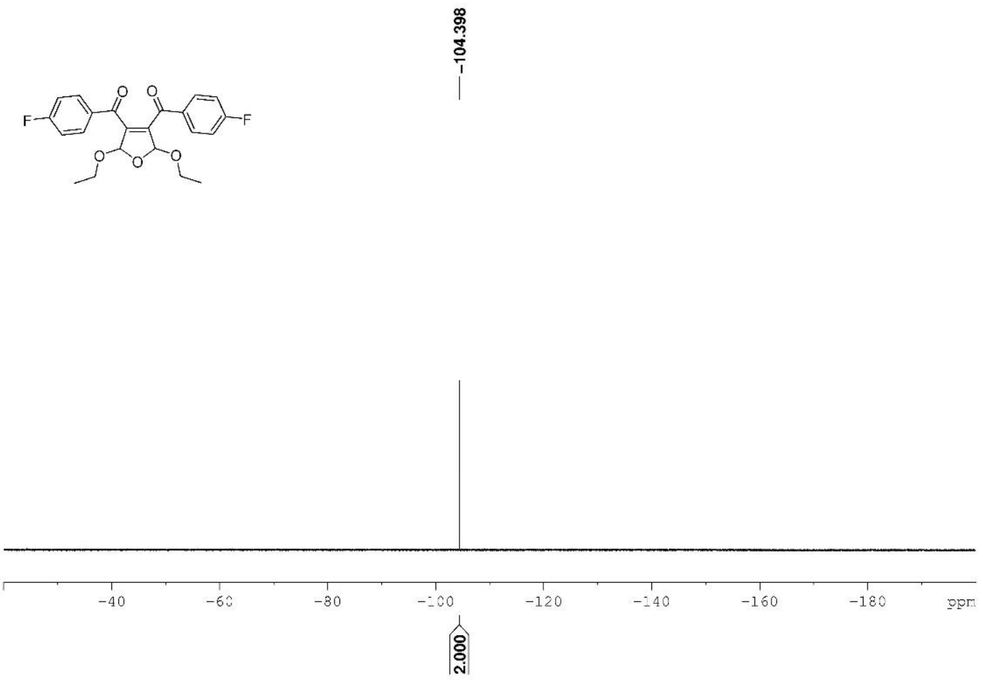

Figure S108. $^{19}F\{^{1}H\}$ NMR spectrum of **17c** (376 MHz, $CDCl_3$).

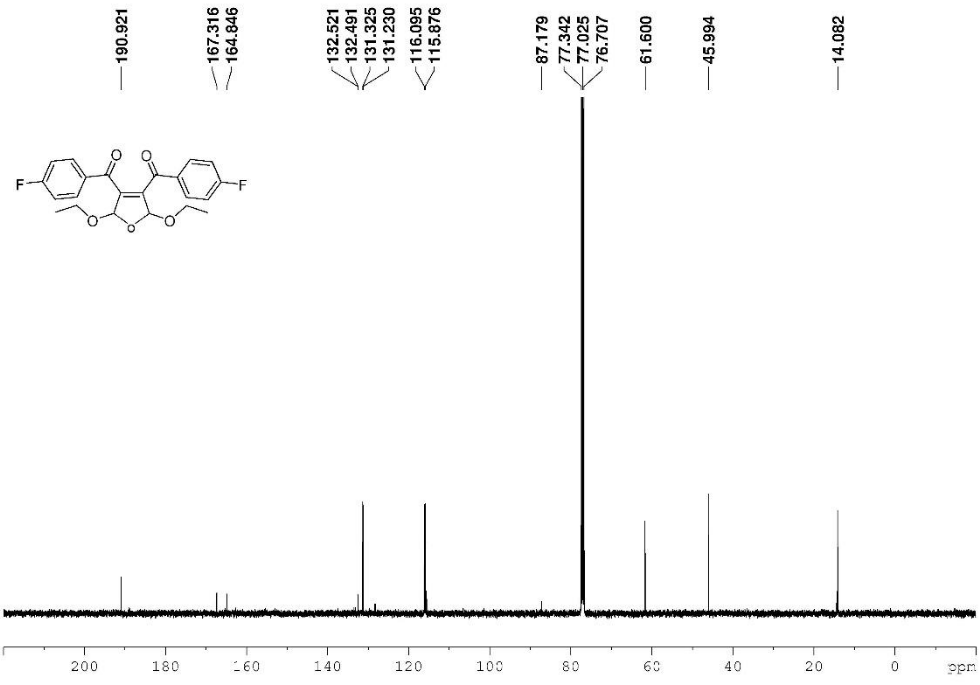

Figure S109. $^{13}C\{^{1}H\}$ NMR spectrum of **17c** (101 MHz, $CDCl_3$).

XI. Single-crystal X-ray diffraction analysis of product 1b

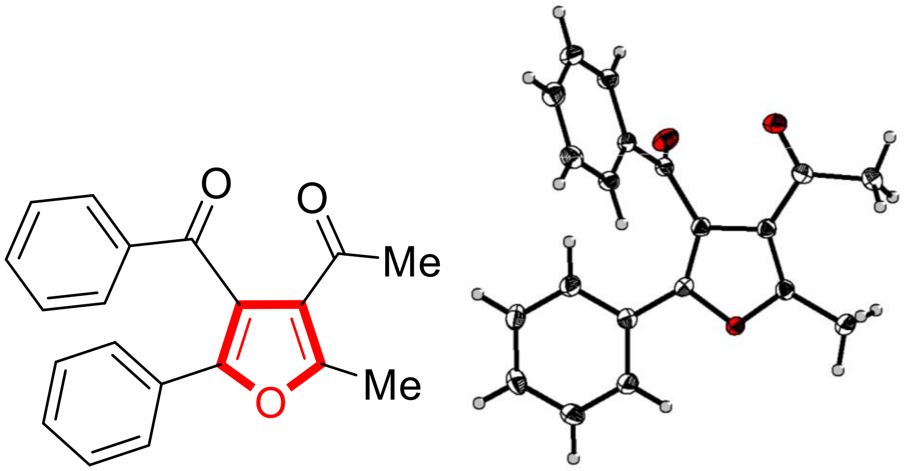

Figure S110. Experimental. Single clear colourless prism crystals of **U020** were used as supplied. A suitable crystal with dimensions 0.13 × 0.09 × 0.04 mm^3 was selected and mounted on a XtaLAB Synergy R, DW system, HyPix-Arc 150 diffractometer. The crystal was kept at a steady T = 123.00(10) K during data collection. The structure was solved with the ShelXT 2018/2 (Sheldrick, 2018) solution program using dual methods and by using Olex2 (Dolomanov et al., 2009) as the graphical interface. The model was refined with ShelXL 2018/3 (Sheldrick, 2015) using full matrix least squares minimisation on $\boldsymbol{F}^2$. **Crystal Data.** $C_{20}H_{16}O_3$, M_r = 304.33, monoclinic, $P2_1/n$ (No.14), a = 7.53430(10) Å, b = 17.21120(10) Å, c = 11.54650(10) Å, β = 95.3790(10)°, $\alpha = \gamma$ = 90°, V = 1490.69(3) $Å^3$, T = 123.00(10) K, Z = 4, Z' = 1, μ(Cu K_α) = 0.730, 16491 reflections measured, 2918 unique (R_{int} = 0.0159) which were used in all calculations. The final wR_2 was 0.0822 (all data) and R_1 was 0.0317 [I≥2σ(I)].

Table S7. Single-crystal X-ray diffraction data and structure refinement of product 1b

**Compound**	**U020**
CCDC number	2113371
Formula	$C_{20}H_{16}O_3$
$D_{calc.}$/ g cm^{-3}	1.356
μ/mm^{-1}	0.730
Formula Weight	304.33
Colour	clear colourless
Shape	prism
Size/mm^3	0.13×0.09×0.04
T/K	123.00(10)
Crystal System	monoclinic
Space Group	$P2_1/n$
a/Å	7.53430(10)
b/Å	17.21120(10)
c/Å	11.54650(10)
α/°	90
β/°	95.3790(10)
γ/°	90
V/Å^3	1490.69(3)
Z	4
Z'	1
Wavelength/Å	1.54184
Radiation type	Cu K$_\alpha$
Θ_{min}/°	4.626
Θ_{max}/°	73.045
Measured Refl's.	16491
Indep't Refl's	2918
Refl's I≥2 σ(I)	2764
R_{int}	0.0159
Parameters	210
Restraints	0
Largest Peak	0.245
Deepest Hole	-0.208
GooF	1.037
wR_2 (all data)	0.0822
wR_2	0.0813
R_1 (all data)	0.0331
R_1	0.0317

XII. Possible mechanism for the transformation of Int4 to Int5 and Int6

The intermediacy of **Int4**, **Int5**, and **Int6** is proposed based on the detection of the dimerization product of **Int5** and literature precedents of furan formation from **Int6**-type structures. A detailed study of the mechanism responsible for the transformation of **Int4** to **Int5** and **Int6** is beyond the scope of this synthetic report, but we postulate a possible pathway as illustrated in Scheme S1. Additional reaction paths are considered in Scheme S2 and Scheme S3.

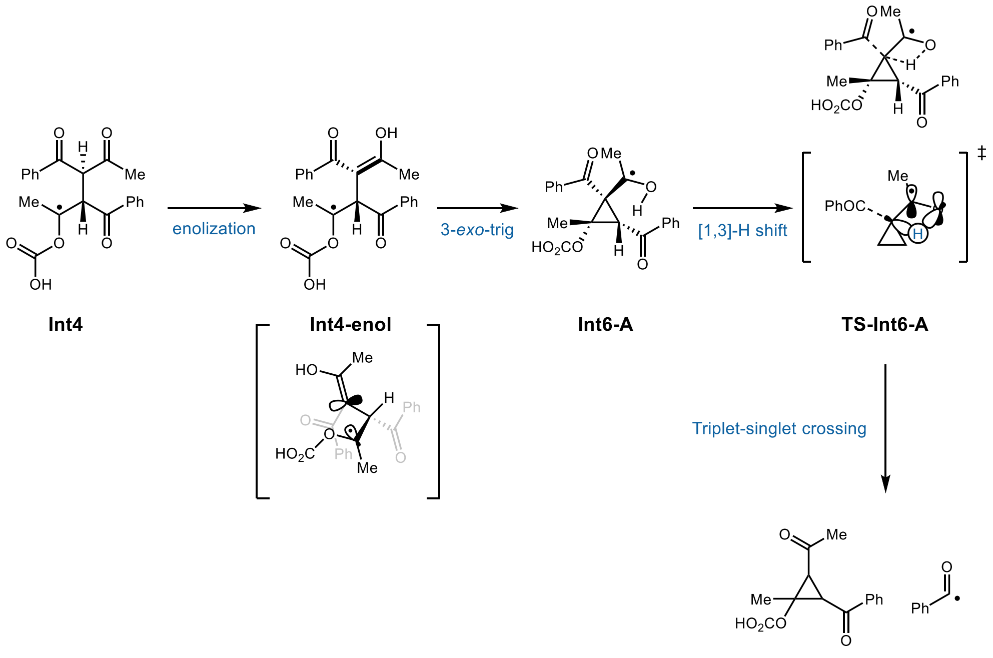

Int4 Int4-enol Int6-A TS-Int6-A

Int6 Int5

Scheme S1. Enolization on the methyl side generates **Int4-enol**, which could undergo 3-*exo*-trig with the existing radical (important orbitals depicted). The resulting α-hydroxy radical **Int6-A** would be viable for a [1,3]-hydrogen shift that ejects the opposing benzoyl radical **Int5**, possibly through transition state **TS-Int6-A** (orbital overlaps depicted). A diradical is formed due to the orthogonal orbital orientation of the C-centered and O-centered radicals, and relaxation yields **Int6**.

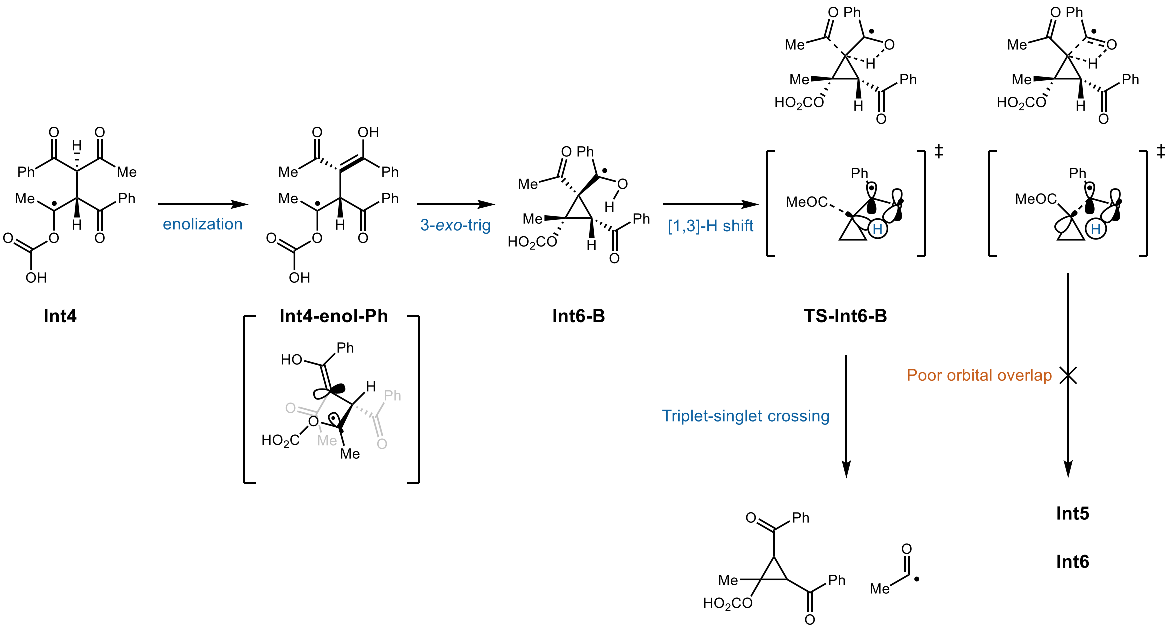

Scheme S2. If enolization occurs on the phenyl side, **Int4-enol-Ph** is formed. The radical cyclization of this species gives **Int6-B**, whose subsequent [1,3]-hydrogen shift via **TS-Int6-B** would give an acetyl radical and a dibenzoyl cyclopropane product. **Int5** and **Int6** would not be formed due to the two necessary orbitals pointing away from each other.

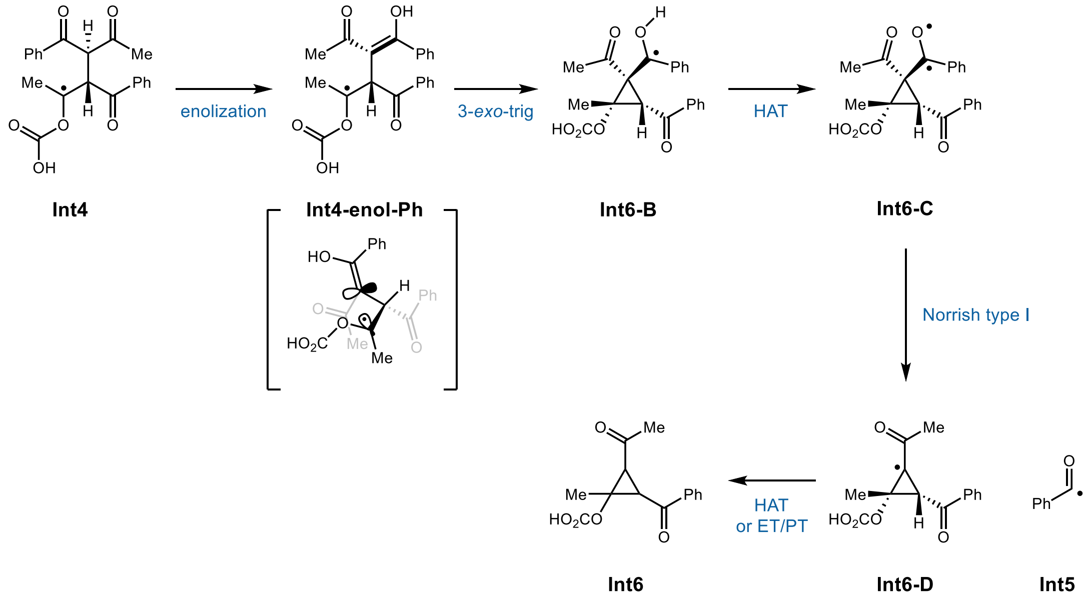

Scheme S3. From **Int6-B**, the direct HAT of the significantly weakened O–H could first yield diradical species **Int6-C** as the two orbitals may be orthogonal to each other. Under Norrish type I reactivity, **Int6-C** could undergo β-scission, affording **Int5** and a radical on the cyclopropane. Thereafter, **Int6-D** could be converted to **Int6** through simple HAT or sequential electron transfer and proton transfer.

XIII. Energy computations of key intermediates

All calculations were performed in the Gaussian 16 series of computer programs.[6] Geometries of molecules (**Int6**, **Int8**, **Int9**) were initially optimized using the DFT method,[7] B3LYP functionals,[8] and the 6-31+G(d,p) basis set,[9] together with CPCM solvation modelling in *N*,*N*-dimethylformamide (DMF),[10] before being subjected to further optimizations and energy calculations at the CBS-QB3 level of theory[11] also with CPCM solvation modelling in DMF. Computational resource restrictions resulted in the use of a lower level of theory for the larger molecules (**Int4**). These radicals and their products (**Int4**–**Int6**) were optimized using (U)ωB97XD functionals[12] and the 6-311++G(d,p) basis set,[13] with CPCM solvation modelling in DMF. Their energies were computed at the same level of theory, and compared to each other rather than against the CBS-QB3 energies of **Int6**. All geometry optimizations and energies were calculated at 298.15 K and 1 atm. The lack of imaginary frequencies in vibrational analysis confirmed that all optimized geometries were local minima of the structures.

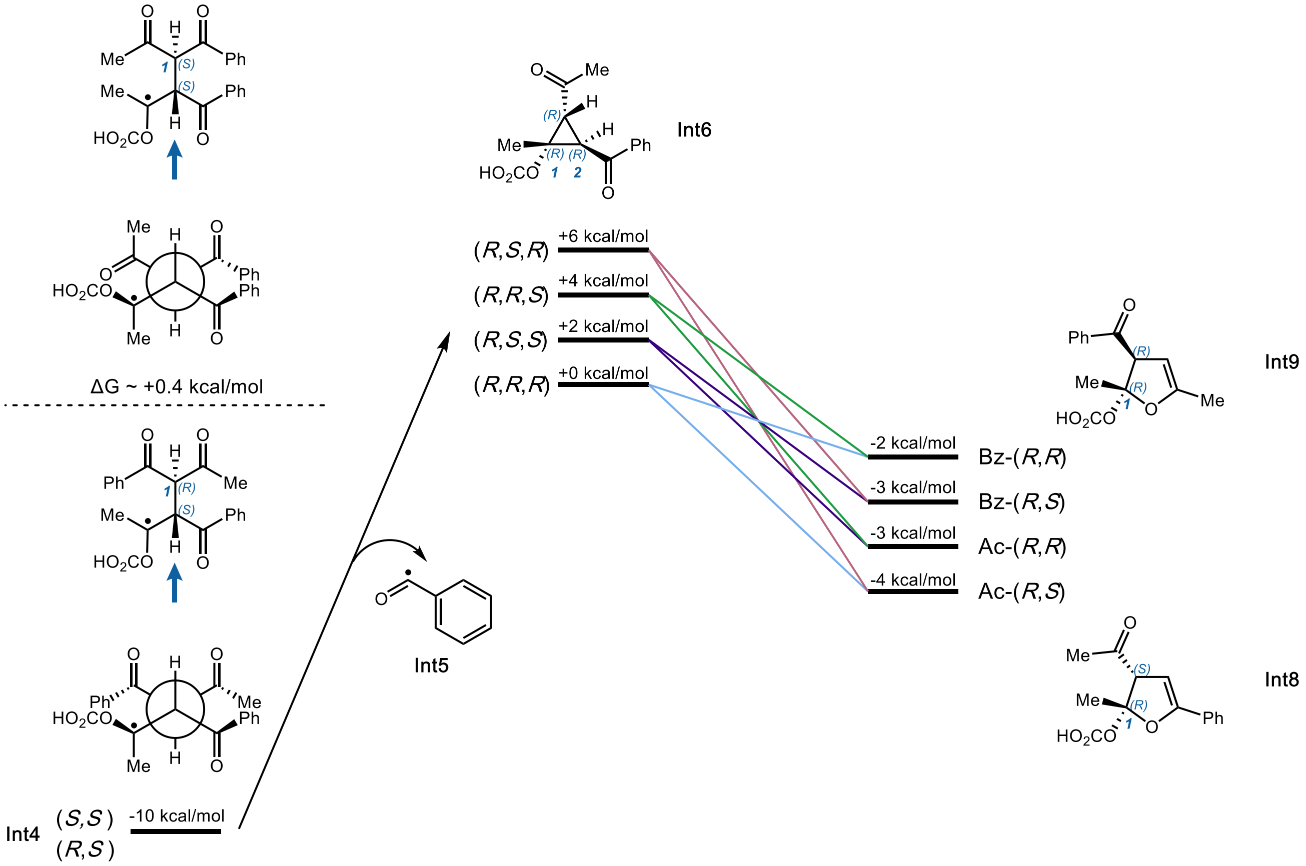

Figure S111. Energy profile of the transformation from **Int4** through **Int6** to **Int8**/**Int9**, considering

the various possible diastereomers.

Optimized geometry:

Optimized geometries in Cartesian coordinates (Å) and energies (hartrees) for stationary points.

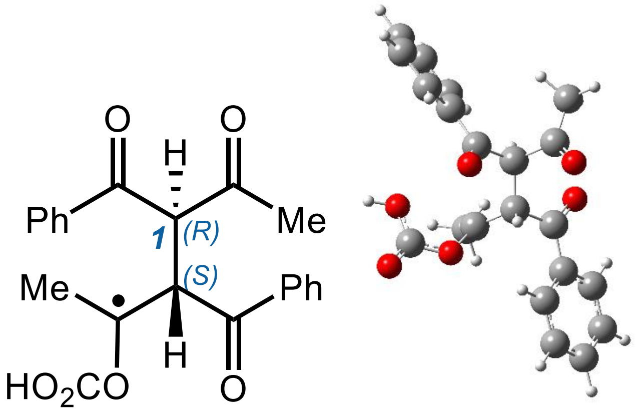

(*R*,*S*)-Int4:

E(UwB97XD) = -1262.943519

Zero-point correction= 0.359061 (Hartree/Particle)

Thermal correction to Energy= 0.384877

Thermal correction to Enthalpy= 0.385822

Thermal correction to Gibbs Free Energy= 0.297095

Sum of electronic and zero-point Energies= -1262.584459

Sum of electronic and thermal Energies= -1262.558642

Sum of electronic and thermal Enthalpies= -1262.557698

Sum of electronic and thermal Free Energies= -1262.646424

Charge = 0 Multiplicity = 2

C	0.60862400	1.00329500	0.55831600
C	0.71204900	-0.27163400	-0.22160400
H	0.90725500	-0.02522600	-1.26674600
C	1.87041400	-1.12372900	0.31416000
C	-0.60488500	-1.05722200	-0.15640900
H	-0.82935800	-1.31959300	0.87552300
C	-1.74075700	-0.22647100	-0.76918600
C	-0.53283300	-2.36640700	-0.96049000
O	1.64703300	-2.17288100	0.88345900
O	-1.53810300	0.37896400	-1.80131700
O	0.16417000	-2.44502700	-1.94489400
C	0.79960700	1.11404000	2.02428900
H	0.17791200	1.90652500	2.44746500
H	0.53623400	0.17297800	2.51177600
H	1.84667200	1.34032800	2.27174100
C	-1.38594000	-3.49271800	-0.45880600
H	-2.40942000	-3.14536100	-0.28968200
H	-1.37973400	-4.32424600	-1.16143200
H	-0.99382600	-3.81892600	0.50906300

C	3.26036800	-0.60139800	0.17343400
C	3.57959200	0.46959000	-0.66570600
C	4.27347000	-1.21897800	0.91319400
C	4.89301200	0.91147400	-0.76365600
H	2.81628700	0.96544800	-1.25193500
C	5.58236500	-0.77202000	0.81919600
H	4.01831700	-2.04730200	1.56315500
C	5.89369600	0.29398500	-0.02137100
H	5.13411900	1.73870900	-1.42069200
H	6.36131200	-1.25232400	1.39974900
H	6.91725300	0.64314500	-0.09710100
C	-3.08385800	-0.22166000	-0.12444300
C	-4.18150500	0.14705400	-0.90989500
C	-3.28149800	-0.54098400	1.22112400
C	-5.45443000	0.18248300	-0.36350200
H	-4.02174100	0.39604300	-1.95204100
C	-4.55660500	-0.48937900	1.77100800
H	-2.44994300	-0.80861800	1.86058700
C	-5.64301800	-0.13415700	0.97996400
H	-6.30110900	0.45731800	-0.98153600
H	-4.69980100	-0.72690800	2.81840400
H	-6.63783200	-0.10252000	1.40950500
O	0.77641500	2.16225300	-0.19692000
C	-0.18256400	3.09292900	-0.26113300
O	-1.26842500	2.76068800	0.43038200
H	-1.92037500	3.46424400	0.32903500
O	-0.03409700	4.10321900	-0.89142800

(*S*,*S*)-Int4:

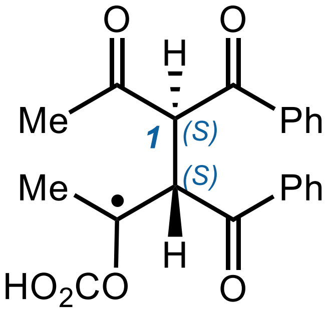

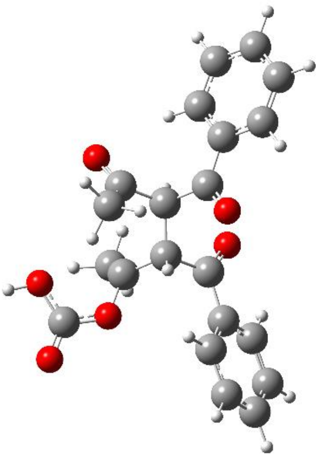

E(UwB97XD) = -1262.942552

Zero-point correction= 0.359570 (Hartree/Particle)

Thermal correction to Energy= 0.385361

Thermal correction to Enthalpy= 0.386305

Thermal correction to Gibbs Free Energy= 0.296835

Sum of electronic and zero-point Energies= -1262.582982

Sum of electronic and thermal Energies= -1262.557191

Sum of electronic and thermal Enthalpies= -1262.556247

Sum of electronic and thermal Free Energies= -1262.645717

Charge = 0 Multiplicity = 2

C	1.37298500	1.21750400	0.70537300
C	0.49035400	0.23713700	-0.00850700
H	0.74815100	0.24758100	-1.06680500
C	0.75432900	-1.16982100	0.56475100
C	-0.99344400	0.59711800	0.15220900
C	-1.86382400	-0.37081500	-0.66145600
O	-0.01976400	-1.64982700	1.36710600
O	-1.38132800	-0.96364400	-1.60483700
C	1.38378800	1.40489500	2.17798800
H	1.71175400	2.41199200	2.44502500
H	0.38811400	1.24633000	2.59414800
H	2.06797900	0.69078600	2.65778500
C	2.01206500	-1.87548900	0.18749500
C	2.76321800	-1.53476500	-0.94006900
C	2.43500700	-2.93421400	0.99862400
C	3.91581600	-2.24523000	-1.25165100
H	2.45942300	-0.72359400	-1.58840500
C	3.59154100	-3.63381100	0.69272100
H	1.84804900	-3.19361000	1.87147900
C	4.33272800	-3.29068400	-0.43574300
H	4.48860100	-1.98010000	-2.13239000
H	3.91704800	-4.44690000	1.33098400

H	5.23558200	-3.83965100	-0.67828400
C	-3.29524400	-0.54456600	-0.28752600
C	-4.05844800	-1.45785600	-1.02353500
C	-3.89587900	0.16747500	0.75474800
C	-5.39548800	-1.65885600	-0.72102800
H	-3.58733700	-2.00523000	-1.83082300
C	-5.23848800	-0.03167200	1.05206400
H	-3.33326800	0.88869900	1.33398100
C	-5.98745100	-0.94467500	0.31846600
H	-5.97893400	-2.37062100	-1.29316700
H	-5.69851700	0.52618900	1.85904700
H	-7.03376700	-1.10074700	0.55553700
O	2.57056700	1.47349600	0.03974200
C	2.99336700	2.72339100	-0.18249800
O	2.07550100	3.63513400	0.12731200
H	2.44026000	4.50882200	-0.05845300
O	4.08141800	2.94704500	-0.63388100
H	-1.27443900	0.53183500	1.20167400
C	-1.26094900	2.04441000	-0.29405900
C	-0.80562000	2.44723500	-1.66874300
H	-1.34961300	3.33318100	-1.99257200
H	0.25942300	2.69424100	-1.62175900
H	-0.92662200	1.63656300	-2.38964800
O	-1.81190300	2.81579300	0.45766900

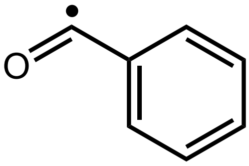

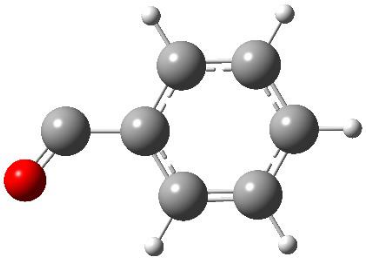

Int5:

E(UwB97XD) = -344.891021

Zero-point correction= 0.098140 (Hartree/Particle)

Thermal correction to Energy= 0.104447

Thermal correction to Enthalpy= 0.105391

Thermal correction to Gibbs Free Energy= 0.066861

Sum of electronic and zero-point Energies= -344.792881

Sum of electronic and thermal Energies= -344.786574

Sum of electronic and thermal Enthalpies= -344.785629

Sum of electronic and thermal Free Energies= -344.824159

Charge = 0 Multiplicity = 2

C	-0.55974400	-0.22905900	-0.00001300
C	0.33458600	-1.29968100	0.00002800
C	1.70154500	-1.05425900	0.00006300
C	2.16778400	0.25645100	0.00005500
C	1.27497300	1.32774900	0.00001300
C	-0.09011900	1.09050600	-0.00002100
H	-0.04775700	-2.31409000	0.00003200
H	2.40164800	-1.88099700	0.00009500
H	3.23487300	0.44775900	0.00008100
H	1.64820000	2.34507800	0.00000700
H	-0.79696700	1.91222500	-0.00005300
C	-2.00779800	-0.50731000	-0.00004900
O	-2.92092000	0.24795400	-0.00007800

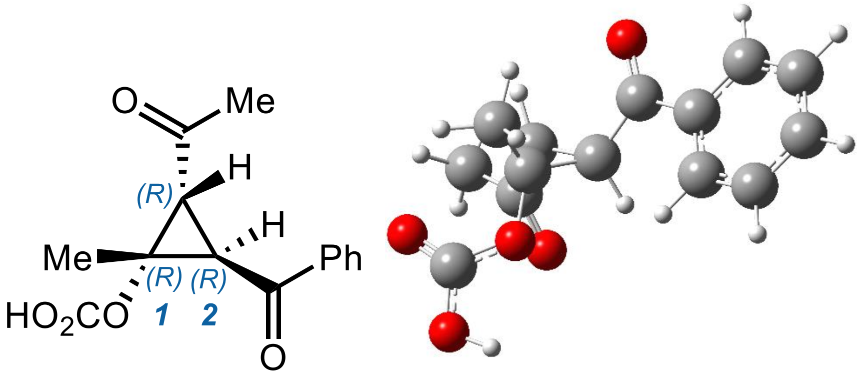

(*R*,*R*,*R*)-Int6 (ωB97XD):

E(RwB97XD) = -918.016737
Zero-point correction= 0.258428 (Hartree/Particle)
Thermal correction to Energy= 0.276514
Thermal correction to Enthalpy= 0.277458
Thermal correction to Gibbs Free Energy= 0.210406
Sum of electronic and zero-point Energies= -917.758309
Sum of electronic and thermal Energies= -917.740223
Sum of electronic and thermal Enthalpies= -917.739279
Sum of electronic and thermal Free Energies= -917.806331
Charge = 0 Multiplicity = 1

C	1.32300600	-1.18974000	0.10644800
C	0.01068300	-0.54306300	-0.26168900
C	0.94785600	0.16414600	0.67024400
H	1.27971200	-1.99107100	0.83507400
H	-0.01510100	-0.11658600	-1.25861500
C	0.66425200	0.33528400	2.13615600
H	1.59533900	0.49457600	2.68202500
H	0.00699600	1.19437600	2.28904400
H	0.17855300	-0.55470600	2.53670100
O	1.52092500	1.31800000	0.07728400
C	2.82981500	1.53649900	0.26810800
O	3.20745900	2.66509000	-0.31718400
H	2.45708200	3.07913300	-0.75997800
O	3.58365500	0.82775800	0.87418300
C	2.39281800	-1.31256900	-0.93335900
O	2.37103600	-0.65221100	-1.95212100
C	3.48723200	-2.29020300	-0.61988900
H	3.93114100	-2.03581000	0.34626500
H	3.06313400	-3.29458200	-0.52825400
H	4.24714900	-2.27782200	-1.39946200
C	-1.27725800	-1.10983100	0.25772900
C	-2.52258600	-0.34449700	-0.02944400
C	-3.75812500	-0.96778300	0.16703400
C	-2.48060000	0.98438100	-0.45826100

C	-4.93482700	-0.27473400	-0.07215500
H	-3.78144800	-1.99784100	0.50243400
C	-3.66122900	1.68149300	-0.68368800
H	-1.53130900	1.48710200	-0.60210600
C	-4.88681500	1.05171100	-0.49595700
H	-5.89073800	-0.76452200	0.07218300
H	-3.62383100	2.71510300	-1.00677700
H	-5.80720400	1.59456900	-0.67906700
O	-1.29742700	-2.14094700	0.90069900

(*R*,*R*,*R*)-Int6 (CBS-QB3):

CBS-QB3 (0 K)= -916.626331

CBS-QB3 Energy= -916.608016

CBS-QB3 Enthalpy= -916.607072

CBS-QB3 Free Energy= -916.674515

Charge = 0 Multiplicity = 1

C	1.24611800	-1.16003600	0.02577800
C	-0.02194900	-0.32817700	-0.10730000
C	1.10448300	0.14510900	0.77868000
H	1.16471600	-2.03362800	0.66162800
H	-0.07484200	0.21381400	-1.04233100
C	1.02868600	0.17104900	2.28303000
H	2.03418900	0.13199200	2.70489400
H	0.54017500	1.09069500	2.61406500
H	0.46045300	-0.68165300	2.65108000
O	1.74196700	1.30418200	0.23677200
C	3.09100400	1.37169800	0.29891600
O	3.52197500	2.53223100	-0.19892300
H	2.76826900	3.06293800	-0.49541200
O	3.82636200	0.52686200	0.73125500
C	2.15741500	-1.28171800	-1.15997600
O	2.10867100	-0.49585200	-2.08769100
C	3.13639800	-2.42748600	-1.11740100
H	3.74443600	-2.35687800	-0.21124800
H	2.59594200	-3.37800700	-1.07117300
H	3.77509200	-2.40767700	-1.99920100
C	-1.30283500	-0.85141600	0.47160300
C	-2.58653600	-0.20990800	0.05796000
C	-3.78705200	-0.84147900	0.41851000
C	-2.63708200	1.00170900	-0.64592300
C	-5.00947700	-0.28055000	0.07405600
H	-3.73841300	-1.77341500	0.96743000
C	-3.86426300	1.56872100	-0.98015800
H	-1.72668200	1.51821200	-0.92152300
C	-5.05000800	0.92756000	-0.62596500

H	-5.93108200	-0.77968700	0.34963900
H	-3.89385100	2.50942100	-1.51704500
H	-6.00401500	1.36742300	-0.89314300
O	-1.29195000	-1.78167500	1.26502200

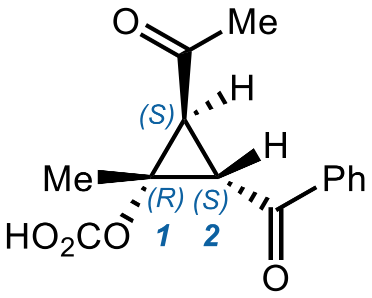

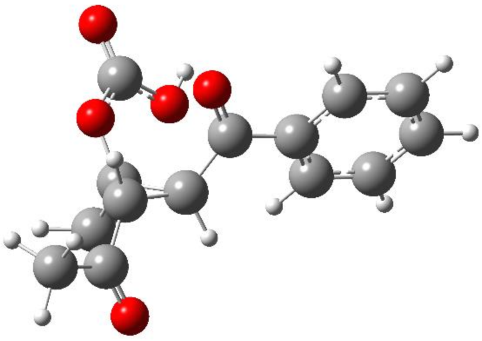

(*R*,*S*,*S*)-Int6 (ωB97XD):

E(RwB97XD) = -918.014033

Zero-point correction= 0.258621 (Hartree/Particle)

Thermal correction to Energy= 0.276617

Thermal correction to Enthalpy= 0.277561

Thermal correction to Gibbs Free Energy= 0.210999

Sum of electronic and zero-point Energies= -917.755412

Sum of electronic and thermal Energies= -917.737416

Sum of electronic and thermal Enthalpies= -917.736472

Sum of electronic and thermal Free Energies= -917.803034

Charge = 0 Multiplicity = 1

C	1.98754100	-0.65020100	-0.48864100
C	0.58963900	-0.58280200	0.08769700
C	1.60585700	0.48229300	0.42383100
C	2.13683100	0.63515100	1.82117300
H	3.11983000	1.11025600	1.79283300
H	1.45863700	1.25010000	2.41443200
H	2.22993600	-0.33960400	2.29811800
O	1.54981300	1.71024400	-0.29142800
C	0.58379600	2.60248200	-0.07584500
O	-0.32802400	2.18743700	0.80042900
H	-1.00008300	2.87336400	0.88949400
O	0.57177100	3.66116700	-0.64296000
H	0.40313100	-1.26865800	0.90495200
C	-0.55487200	-0.30041000	-0.83320900
C	-1.93402200	-0.54677500	-0.32156300
C	-2.97025500	-0.69466900	-1.24711500
C	-2.21775400	-0.60155500	1.04473900
C	-4.27010700	-0.90814400	-0.81295200
H	-2.74253300	-0.64774500	-2.30541700
C	-3.52300800	-0.79839000	1.47785700
H	-1.43083500	-0.46348400	1.77649000
C	-4.54782200	-0.95775300	0.55088200
H	-5.06814300	-1.03418200	-1.53533300
H	-3.73985300	-0.82716500	2.53921200
O	-0.36713100	0.11671800	-1.96039800

H	-5.56444800	-1.11978000	0.89089200
H	2.06553100	-0.35157600	-1.52807100
C	2.93041100	-1.72049100	-0.03333800
C	4.14887700	-1.91390100	-0.88831700
H	3.84188700	-2.21952800	-1.89307100
H	4.67851100	-0.96253000	-0.99236100
H	4.80615600	-2.66650000	-0.45606900
O	2.71752200	-2.37581300	0.96594600

(*R*,*S*,*S*)-Int6 (CBS-QB3):

CBS-QB3 (0 K)= -916.623323

CBS-QB3 Energy= -916.605054

CBS-QB3 Enthalpy= -916.604110

CBS-QB3 Free Energy= -916.671308

Charge = 0 Multiplicity = 1

C	1.94639100	-0.69910300	-0.50109600
C	0.56164500	-0.55193100	0.11950000
C	1.65277900	0.45047900	0.43237400
C	2.23587600	0.56439100	1.81729700
H	3.23136800	1.01153200	1.76164100
H	1.60021200	1.19256700	2.44277300
H	2.31887200	-0.41957400	2.27543100
O	1.65523500	1.69236200	-0.28022800
C	0.76788400	2.66622900	-0.01985200
O	-0.14358700	2.31487900	0.89913400
H	-0.74331700	3.06548500	1.01951500
O	0.81895300	3.72834700	-0.57929800
H	0.37150600	-1.23681400	0.93516600
C	-0.58826000	-0.21590500	-0.78236500
C	-1.97687000	-0.48695000	-0.29973800
C	-3.02084600	-0.42145600	-1.23524800
C	-2.27773400	-0.77782900	1.03818000
C	-4.33333800	-0.65343000	-0.84540800
H	-2.78028800	-0.18962700	-2.26506300
C	-3.59578200	-0.99855400	1.42974500
H	-1.49497100	-0.81629600	1.78446600
C	-4.62350600	-0.94196900	0.48986500
H	-5.13121900	-0.60944500	-1.57750700
H	-3.81933900	-1.21456000	2.46778700
O	-0.39207400	0.26066300	-1.89041000
H	-5.64787200	-1.12095700	0.79589900
H	1.99827400	-0.39402600	-1.53946700
C	2.81821100	-1.84830500	-0.09099300
C	3.91426000	-2.21958000	-1.05791300
H	3.47561800	-2.52529400	-2.01308500

H	4.54527700	-1.34935300	-1.26236100
H	4.51561700	-3.03120200	-0.65127500
O	2.64597400	-2.44112100	0.95830400

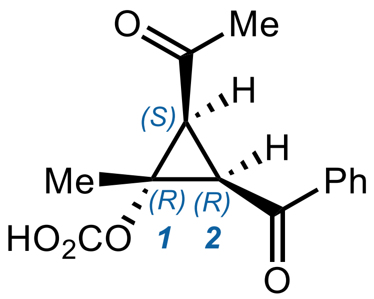

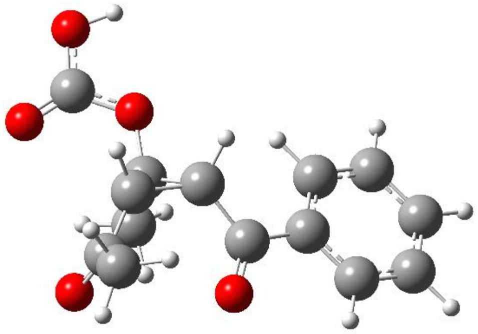

(*R*,*R*,*S*)-Int6 (ωB97XD):

E(RwB97XD) = -918.010683

Zero-point correction= 0.258265 (Hartree/Particle)

Thermal correction to Energy= 0.276437

Thermal correction to Enthalpy= 0.277381

Thermal correction to Gibbs Free Energy= 0.209490

Sum of electronic and zero-point Energies= -917.752418

Sum of electronic and thermal Energies= -917.734246

Sum of electronic and thermal Enthalpies= -917.733302

Sum of electronic and thermal Free Energies= -917.801193

Charge = 0 Multiplicity = 1

C	-1.56226900	0.80386800	-0.59308300
C	-0.16539100	0.23805900	-0.76118000
C	-1.14309000	-0.37787600	0.21526000
H	0.00560500	-0.33739100	-1.66363900
C	-0.88844800	-0.37218500	1.69709200
H	-1.82108700	-0.51715700	2.24256600
H	-0.19529400	-1.17972400	1.94574500
H	-0.45901300	0.57703000	2.01287200
O	-1.61758700	-1.63813500	-0.25476600
C	-2.91307000	-1.92128100	-0.05825800
O	-3.20281100	-3.13327000	-0.51812900
H	-2.41507500	-3.54632600	-0.89112900
O	-3.72673800	-1.20496100	0.45267700
C	1.03754100	0.94967300	-0.22148100
C	2.30815600	0.17645200	-0.14613700
C	3.50317200	0.86299200	0.08662100
C	2.32470700	-1.21493800	-0.27179800
C	4.69905400	0.16833900	0.18314700
H	3.48071000	1.94172600	0.18547700
C	3.52256500	-1.91015200	-0.16222500
H	1.40584900	-1.76417100	-0.44116000
C	4.70892500	-1.21953800	0.06061400
H	5.62471400	0.70496600	0.35530400
H	3.52940200	-2.99000000	-0.25148700
H	5.64379200	-1.76270000	0.13996800

O	0.97003300	2.10446300	0.15328400
H	-2.20220500	0.58547400	-1.44308500
C	-1.84228600	2.17229300	-0.02053100
C	-1.82221600	3.28781700	-1.02812900
H	-0.89909500	3.25180700	-1.60957300
H	-2.65643300	3.15303800	-1.72408300
H	-1.91979000	4.25085600	-0.52893600
O	-2.13866900	2.34333100	1.14089500

(*R*,*R*,*S*)-Int6 (CBS-QB3):

CBS-QB3 (0 K)= -916.619774

CBS-QB3 Energy= -916.601305

CBS-QB3 Enthalpy= -916.600361

CBS-QB3 Free Energy= -916.668814

Charge = 0 Multiplicity = 1

C	-1.47489300	0.76676000	-0.65485100
C	-0.11978500	0.06189200	-0.57159600
C	-1.29249200	-0.34410100	0.31848200
H	0.07644600	-0.63170100	-1.37790600
C	-1.26815000	-0.18886200	1.81680300
H	-2.28726800	-0.17776500	2.20363900
H	-0.72540800	-1.02730700	2.26017100
H	-0.78624800	0.74284500	2.10306200
O	-1.81349000	-1.62007100	-0.09244500
C	-3.15573800	-1.78339900	-0.06493500
O	-3.47941400	-3.02446300	-0.44103600
H	-2.67612600	-3.52575000	-0.64275600
O	-3.96964300	-0.95747100	0.24311600
C	1.07494300	0.74018200	0.02167900
C	2.39311400	0.04173400	-0.03976000
C	3.54458300	0.77102900	0.29487800
C	2.51768600	-1.31080100	-0.38838700
C	4.79371900	0.16529400	0.26952100
H	3.43789400	1.81279600	0.56981400
C	3.77004800	-1.91927400	-0.40186200
H	1.64289800	-1.90016300	-0.63215100
C	4.90837500	-1.18248400	-0.07882200
H	5.67845700	0.73834700	0.52101900
H	3.85657400	-2.96687700	-0.66465800
H	5.88306200	-1.65642400	-0.09686300
O	0.97023500	1.84018300	0.54750400
H	-2.01047700	0.51013800	-1.56417500
C	-1.70929400	2.21323400	-0.26123200
C	-1.33649900	3.23349500	-1.31018100
H	-0.26511200	3.17796400	-1.51624300

H	-1.86095900	3.02123800	-2.24681900
H	-1.59560100	4.23286200	-0.96238400
O	-2.23791800	2.51952200	0.78633900

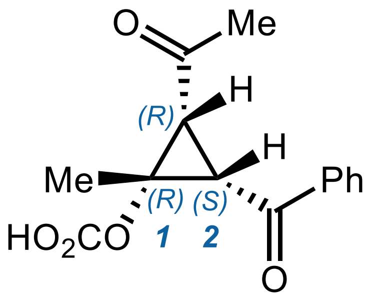

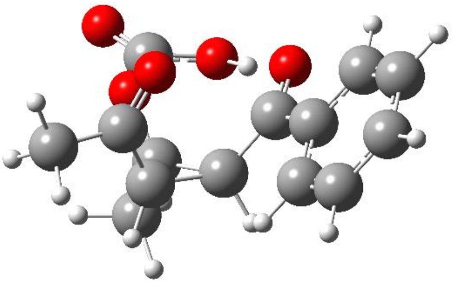

(*R*,*S*,*R*)-Int6 (ωB97XD):

E(RwB97XD) = -918.009588

Zero-point correction= 0.258252 (Hartree/Particle)

Thermal correction to Energy= 0.275852

Thermal correction to Enthalpy= 0.276796

Thermal correction to Gibbs Free Energy= 0.211500

Sum of electronic and zero-point Energies= -917.751336

Sum of electronic and thermal Energies= -917.733736

Sum of electronic and thermal Enthalpies= -917.732792

Sum of electronic and thermal Free Energies= -917.798088

Charge = 0 Multiplicity = 1

C	-0.68942400	1.47724700	0.63558900
C	-0.33713300	0.07623900	1.09842700
C	-1.76687800	0.42347500	0.80896200
H	-0.68592100	2.26887300	1.37588500
C	-2.75382500	0.55965100	1.93757500
H	-3.54812600	1.25284500	1.65392400
H	-3.19560700	-0.41023300	2.18027900
H	-2.25984000	0.93921300	2.83261200
O	-2.37827100	0.05389100	-0.41517700
C	-3.09581200	-1.09944400	-0.49547200
O	-2.54326500	-2.16838100	0.03432800
H	-1.54863000	-2.07786900	0.06608500
O	-4.16067300	-1.10282100	-1.04950800
C	-0.34463000	1.89945200	-0.76013400
O	0.10114000	1.11150100	-1.56698900
C	-0.60491100	3.34084900	-1.08278900
H	-1.64864600	3.58314600	-0.86306700
H	0.01355900	3.97437500	-0.44009300
H	-0.38255700	3.54424200	-2.12882000
H	-0.09891000	0.00229900	2.15644900
C	0.51276900	-0.86596600	0.29554200
C	1.96768600	-0.60884700	0.20183800
C	2.76428500	-1.50805800	-0.51321500
C	2.55619100	0.50045200	0.81440300
C	4.13009300	-1.29694200	-0.61803900
H	2.30063900	-2.36607300	-0.98434300

C	3.92528100	0.70623300	0.71270400
H	1.95342100	1.20731000	1.37145600
C	4.71146300	-0.18976000	-0.00438600
H	4.74411700	-1.99375400	-1.17624100
H	4.37815300	1.56651400	1.19086800
O	0.02450200	-1.88828500	-0.16954900
H	5.77995300	-0.02561000	-0.08586700

(*R*,*S*,*R*)-Int6 (CBS-QB3):

CBS-QB3 (0 K)= -916.618664

CBS-QB3 Energy= -916.600750

CBS-QB3 Enthalpy= -916.599806

CBS-QB3 Free Energy= -916.665699

Charge = 0 Multiplicity = 1

C	-0.73048600	1.49414000	0.64132800
C	-0.31745000	0.09555600	1.08346700
C	-1.77031000	0.39684100	0.83527300
H	-0.73488000	2.27729600	1.39041300
C	-2.73370300	0.48597800	1.99451100
H	-3.56772200	1.14243400	1.73726700
H	-3.12661300	-0.50379600	2.24148700
H	-2.23662500	0.88233800	2.88063200
O	-2.40900400	0.02557000	-0.38579400
C	-3.09554100	-1.16340300	-0.48190400
O	-2.48977500	-2.22214300	0.02712800
H	-1.49394800	-2.09088700	0.02807900
O	-4.16539100	-1.19053600	-1.02592800
C	-0.43016800	1.94189400	-0.76010900
O	0.05066100	1.18336500	-1.57910900
C	-0.77673400	3.37288300	-1.08192100
H	-1.83247700	3.55968100	-0.86393600
H	-0.19477200	4.04816200	-0.44711400
H	-0.56873700	3.58156300	-2.13011200
H	-0.05308600	0.02831800	2.13571600
C	0.54288300	-0.82632200	0.25914400
C	2.00123500	-0.57384000	0.17774500
C	2.80146000	-1.48317400	-0.53178200
C	2.60166500	0.52912100	0.80153000
C	4.17335600	-1.28960500	-0.61900500
H	2.33053400	-2.33356600	-1.00779200
C	3.97762000	0.71673200	0.71757400
H	2.00184300	1.24128800	1.35317100
C	4.76385200	-0.18943800	0.00680400
H	4.78498100	-1.99344600	-1.17087900
H	4.43577200	1.56948800	1.20402000

O	0.06030800	-1.84794900	-0.22966800
H	5.83526600	-0.03952000	-0.05992200

(*R*,*S*)-Int8: 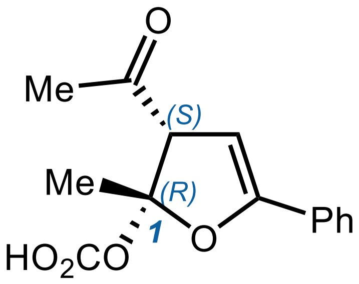

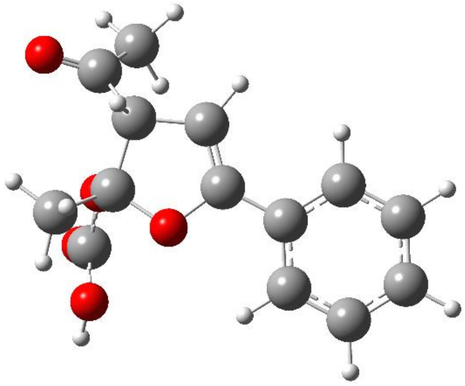

CBS-QB3 (0 K)= -916.634027

CBS-QB3 Energy= -916.616169

CBS-QB3 Enthalpy= -916.615224

CBS-QB3 Free Energy= -916.681489

Charge = 0 Multiplicity = 1

C	1.28561900	0.20195300	0.85465600
O	-0.09563100	0.50759400	0.89145200
C	1.36144700	-1.34770200	0.69345300
C	-0.03092600	-1.65927000	0.21666600
C	-0.80300800	-0.57884600	0.38081300
C	2.01002200	0.76312800	2.05757800
H	1.58882300	0.32174300	2.96168300
H	3.06563300	0.49432000	1.99610500
H	1.90812900	1.84473100	2.10855900
O	1.84111100	0.71710800	-0.40616400
C	1.79449000	1.99311700	-0.81754100
O	1.22563600	2.83197100	0.05840800
H	1.25149500	3.71713200	-0.33354100
O	2.23777900	2.31521000	-1.88878400
H	-0.35873200	-2.62366500	-0.13657600
H	1.54259700	-1.78476300	1.68169600
C	2.56621100	-1.78264200	-0.16256100
C	2.32691100	-2.17519100	-1.59640500
H	1.77940800	-3.12278800	-1.63087400
H	1.71043800	-1.42690000	-2.09830500
H	3.28097600	-2.29383900	-2.10845700
O	3.66733800	-1.81152200	0.34704300
C	-2.22778600	-0.34181900	0.14190000
C	-2.75797100	0.95315400	0.24076500
C	-3.08618900	-1.40315100	-0.18990200
C	-4.11176900	1.18067100	0.00523900
H	-2.10430700	1.77621700	0.49715200
C	-4.43513000	-1.17001600	-0.42615000
H	-2.69942600	-2.41299400	-0.25702400

C	-4.95433800	0.12303100	-0.32961700
H	-4.50700400	2.18696400	0.08350800
H	-5.08557400	-1.99870300	-0.68116000
H	-6.00782200	0.30114700	-0.51177700

(*R*,*R*)-Int8: 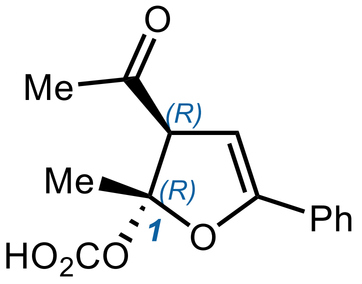

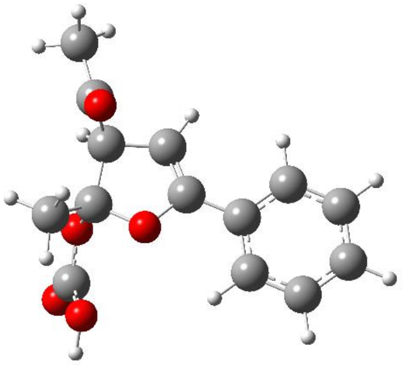

CBS-QB3 (0 K)= -916.631881

CBS-QB3 Energy= -916.614100

CBS-QB3 Enthalpy= -916.613155

CBS-QB3 Free Energy= -916.679392

Charge = 0 Multiplicity = 1

C	1.49283200	0.31354200	0.30643900
O	0.12846500	0.36887800	0.68578600
C	1.64493500	-0.99119600	-0.52804400
C	0.20207200	-1.26112700	-0.89300200
C	-0.58955000	-0.48512000	-0.14248800
C	2.38898800	0.52304800	1.50676800
H	2.22491400	-0.28209500	2.21880200
H	3.43400200	0.51284200	1.19214700
H	2.16501600	1.47752300	1.97746700
O	1.74144600	1.34670200	-0.71955100
C	1.49585200	2.65652600	-0.58489100
O	0.99473300	3.00978900	0.60798300
H	0.86935400	3.96983100	0.58984600
O	1.71890000	3.42545000	-1.48458500
H	-0.11670700	-1.99284700	-1.61765200
C	-2.04657800	-0.37043100	-0.05735100
C	-2.63461000	0.70933000	0.61811600
C	-2.87791800	-1.33660500	-0.64753600
C	-4.02069300	0.82366700	0.69196300
H	-2.00088100	1.45686700	1.07648800
C	-4.25996900	-1.21536400	-0.57414200
H	-2.44217100	-2.18822100	-1.15621600
C	-4.83769500	-0.13482300	0.09617400
H	-4.46148500	1.66423800	1.21553700
H	-4.88938100	-1.96856200	-1.03389300
H	-5.91623200	-0.04513100	0.15509400
C	2.20016500	-2.23477000	0.19315300
O	1.98831500	-2.45042900	1.36763700
C	2.97246300	-3.19318000	-0.67874400

H	2.41158100	-3.42602200	-1.58850000
H	3.90615000	-2.71714400	-0.99613400
H	3.19360500	-4.10743400	-0.13000800
H	2.26560900	-0.77794900	-1.39962400

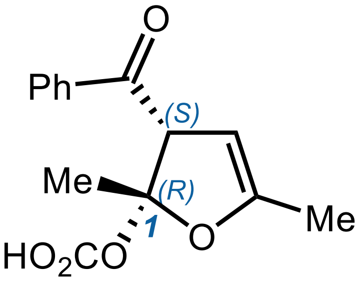

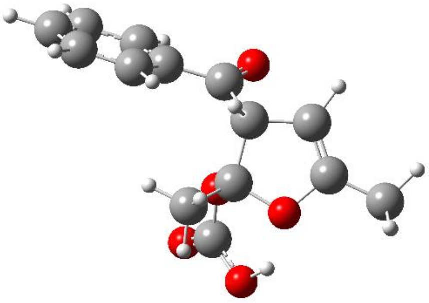

(*R*,*S*)-Int9:

CBS-QB3 (0 K)= -916.632684

CBS-QB3 Energy= -916.615346

CBS-QB3 Enthalpy= -916.614402

CBS-QB3 Free Energy= -916.678991

Charge = 0 Multiplicity = 1

C	-1.09986800	0.05563600	0.72731600
O	-2.50675900	-0.22811200	0.70722800
C	-0.41399200	-1.26810800	0.25762800
C	-1.58279800	-2.03280200	-0.30579700
C	-2.71979200	-1.41155900	-0.02019800
C	-0.72458000	0.58553400	2.09499300
H	-0.90735200	-0.18099300	2.84876400
H	0.32865900	0.86460400	2.11340400
H	-1.32575700	1.46263600	2.34028800
O	-0.80130100	1.04152100	-0.29938400
C	-1.45082100	2.23539900	-0.42489900
O	-2.67736600	2.31530500	0.09285200
H	-2.99029400	1.42752800	0.36512900
O	-0.92771000	3.14409000	-1.00552600
H	-1.49559700	-2.96459200	-0.84069200
C	-4.14519300	-1.72634400	-0.28328100
H	-4.70127100	-1.80144200	0.65569800
H	-4.61066700	-0.93711900	-0.88104700
H	-4.22944100	-2.67115100	-0.81941000
C	0.72430100	-1.04862400	-0.75710300
H	-0.01076100	-1.77902300	1.13663900
O	0.52495800	-1.25603000	-1.93855400
C	2.06394600	-0.59121100	-0.27634100
C	2.93891500	-0.03279900	-1.22160400
C	2.49216300	-0.71942700	1.05231700
C	4.20260400	0.40169700	-0.84520300
H	2.60482800	0.05778400	-2.24732500
C	3.76615300	-0.29613300	1.42566100
H	1.85409000	-1.17006600	1.80114200
C	4.61991600	0.26922400	0.48113000

H	4.86474800	0.84198800	-1.58153300
H	4.09036900	-0.41086400	2.45316400
H	5.60813800	0.60364000	0.77514600

(*R*,*R*)-Int9:

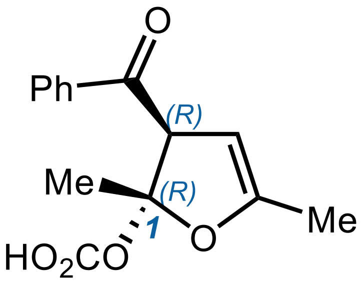

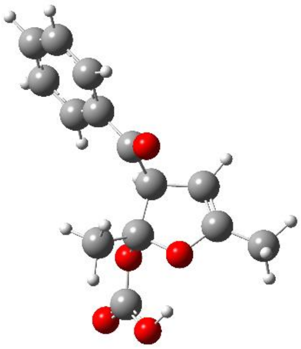

CBS-QB3 (0 K)= -916.630873

CBS-QB3 Energy= -916.613584

CBS-QB3 Enthalpy= -916.612639

CBS-QB3 Free Energy= -916.678054

Charge = 0 Multiplicity = 1

C	1.23160500	-0.14107700	0.39932200
O	2.22298000	0.89357200	0.45733800
C	0.10578600	0.41367200	-0.52700200
H	-0.14332500	-0.36658600	-1.24160100
C	0.81583200	1.57152000	-1.18655500
C	1.98104900	1.79586000	-0.59038600
C	-1.15193100	0.89203000	0.22839800
C	0.88024200	-0.57507500	1.80671800
H	0.46870700	0.26641900	2.36136400
H	0.14762400	-1.38242500	1.77536300
H	1.77277700	-0.93005100	2.32383100
O	1.76488800	-1.27157700	-0.35468600
C	2.95806600	-1.87198700	-0.08116000
O	3.86475600	-1.15293700	0.58398800
H	3.57685800	-0.21894400	0.64878700
O	3.16769900	-2.99032100	-0.45995500
O	-1.05957000	1.83908900	0.99012800
C	-2.46272700	0.21332300	0.00464400
C	-3.61026700	0.82330700	0.53968300
C	-2.59946600	-0.99896900	-0.68807600
C	-4.86026100	0.24329200	0.37797900
H	-3.49706200	1.75606700	1.07729300
C	-3.85374400	-1.58546900	-0.83919200
H	-1.73692500	-1.50609300	-1.09951600
C	-4.98439500	-0.96498800	-0.31239800
H	-5.73880100	0.72738300	0.78808300
H	-3.94616100	-2.52575400	-1.36947000
H	-5.96004300	-1.42033800	-0.43758600
H	0.40604200	2.14937600	-2.00008300
C	3.04822100	2.80453600	-0.80051200

H	3.21305300	3.38219400	0.11366200
H	3.99367500	2.31945800	-1.06038000
H	2.76951800	3.48682100	-1.60302400

XIV. References

[1] R. K. Harris, E. D. Becker, S. M. C. De Menezes, R. Goodfellow and P. Granger, *Magn. Reason. Chem.* 2002, **40**, 489-505.

[2] H. E. Gottlieb, V. Kotlyar and A. Nudelman, *J. Org. Chem.* 1997, **62**, 7512-7515.

[3] G. M. Sheldrick, *Acta Crystallogr., Sect. A: Found. Adv.* 2015, **71**, 3-8.

[4] G. M. Sheldrick, *Acta Crystallogr., Sect. A: Found. Crystallogr.* 2008, **64**, 112-122.

[5] H. Putz and K. Brandenburg GbR, *Diamond, crystal and molecular structure visualization, crystal impact*, Bonn (Germany), 2017.

[6] Gaussian 16, Revision C.01, M. J. Frisch, G. W. Trucks, H. B. Schlegel, G. E. Scuseria, M. A. Robb, J. R. Cheeseman, G. Scalmani, V. Barone, G. A. Petersson, H. Nakatsuji, X. Li, M. Caricato, A. V. Marenich, J. Bloino, B. G. Janesko, R. Gomperts, B. Mennucci, H. P. Hratchian, J. V. Ortiz, A. F. Izmaylov, J. L. Sonnenberg, D. Williams-Young, F. Ding, F. Lipparini, F. Egidi, J. Goings, B. Peng, A. Petrone, T. Henderson, D. Ranasinghe, V. G. Zakrzewski, J. Gao, N. Rega, G. Zheng, W. Liang, M. Hada, M. Ehara, K. Toyota, R. Fukuda, J. Hasegawa, M. Ishida, T. Nakajima, Y. Honda, O. Kitao, H. Nakai, T. Vreven, K. Throssell, J. A. Montgomery, Jr., J. E. Peralta, F. Ogliaro, M. J. Bearpark, J. J. Heyd, E. N. Brothers, K. N. Kudin, V. N. Staroverov, T. A. Keith, R. Kobayashi, J. Normand, K. Raghavachari, A. P. Rendell, J. C. Burant, S. S. Iyengar, J. Tomasi, M. Cossi, J. M. Millam, M. Klene, C. Adamo, R. Cammi, J. W. Ochterski, R. L. Martin, K. Morokuma, O. Farkas, J. B. Foresman, and D. J. Fox, Gaussian, Inc., Wallingford CT, 2019.

[7] R. G. Parr and W. Yang, *Density-Functional Theory of Atoms and Molecules*. University Press: Oxford, 1989.

[8] a) R. G. Parr and W. Yang, *Annu. Rev. Phys. Chem.* 1995, **46**, 701-728; b) P. J. Stephens, F. J. Devlin, C. F. Chabalowski and M. J. Frisch, *J. Phys. Chem.* 1994, **98**, 11623-11627; c) C. Lee, W. Yang and R. G. Parr, *Phys. Rev. B*, 1988, **37**, 785-789; d) A. D. Becke, *Phys. Rev. A*, 1988, **38**, 3098-3100; e) A. D. Becke, *J. Chem. Phys.* 1993, **98**, 5648-5652.

[9] a) R. Ditchfield, W. J. Hehre and J. A. Pople, *J. Chem. Phys.* 1971, **54**, 724-728; b) W. J. Hehre, R. Ditchfield and J. A. Pople, *J. Chem. Phys.* 1972, **56**, 2257-2261; c) P. C. Hariharan and J. A. Pople, *Theor. Chem. Acc.* 1973, **28**, 213-222; d) P. C. Hariharan and J. A. Pople, *Mol. Phys.* 1974, **27**,

209-214; e) M. S. Gordon, *Chem. Phys. Lett.* 1980, **76**, 163-168; f) M. M. Francl, W. J. Pietro, W. J. Hehre, J. S. Binkley, D. J. DeFrees, J. A. People and M. S. Gordon, *J. Chem. Phys.* 1982, **77**, 3654-3665; g) R. C. Binning and L. A. Curtiss, *J. Comp. Chem.* 1990, **11**, 1206-1216; h) J.-P. Blaudeau, M. P. McGrath, L. A. Curtiss and L. Radom, *J. Chem. Phys.* 1997, **107**, 5016-5021; i) V. A. Rassolov, J. A. Pople, M. A. Ratner and T. L. Windus, *J. Chem. Phys.* 1998, **109**, 1223-1229; j) V. A. Rassolov, M. A. Ratner, J. A. Pople, P. C. Redfern and L. A. Curtiss, *J. Comp. Chem.* 2001, **22**, 976-984; k) T. Clark, J. Chandrasekhar, G. W. Spitznagel and P. V. R. Schleyer, *J. Comp. Chem.* 1983, **4**, 294-301; l) M. J. Frisch, J. A. People and J. S. Binkley, *J. Chem. Phys.* 1984, **80**, 3265-3269.

[10] a) V. Barone and M. Cossi, *J. Phys. Chem. A*, 1998, **102**, 1995-2001; b) M. Cossi, N. Rega, G. Scalmani and V. Barone, *J. Comput. Chem.* 2003, **24**, 669-681.

[11] a) M. R. Nyden and G. A. Petersson, *J. Chem. Phys.* 1981, **75**, 1843-1862; b) G. A. Petersson, A. Bennett, T. G. Tensfeldt, M. A. Al-Laham and W. A. Shirley, *J. Chem. Phys.* 1988, **89**, 2193-2218; c) G. A. Petersson and M. A. AlLaham, *J. Chem. Phys.* 1991, **94**, 6081-6090; d) G. A. Petersson, T. G. Tensfeldt and J. A. Montgomery, Jr., *J. Chem. Phys.* 1991, **94**, 6091-6101; e) J. A. Montgomery, Jr., J. W. Ochterski and G. A. Petersson, *J. Chem. Phys.* 1994, **101**, 5900-5909; f) J. A. Montgomery, Jr. and M. J. Frisch, *J. Chem. Phys.* 1999, **110**, 2822-2827; g) J. A. Montgomery, Jr., M. J. Frisch and J. W. Ochterski, *J. Chem. Phys.* 2000, **112**, 6532-6542.

[12] J. D. Chai and M. Head-Gordon, *Phys. Chem. Chem. Phys.* 2008, **10**, 6615-6620.

[13] a) A. D. McLean and G. S. Chandler, *J. Chem. Phys.* 1980, **72**, 5639-5648; b) J.-P. Blaudeau, M. P. McGrath, L. A. Curtiss and L. Radom, *J. Chem. Phys.* 1997, **107**, 5016-5021; c) A. J. H. Wachters, *J. Chem. Phys.* 1970, **52**, 1033-1037; d) P. J. Hay, *J. Chem. Phys.* 1977, **66**, 4377-4384; e) K. Raghavachari and G. W. Trucks, *J. Chem. Phys.* 1989, **91**, 1062-1065; f) R. C. Binning and L. A. Curtiss, *J. Comp. Chem.* 1990, **11**, 1206-1216; g) M. P. McGrath and L. Radom, *J. Chem. Phys.* 1991, **94**, 511-516; h) L. A. Curtiss, M. P. McGrath, J.-P. Blaudeau, N. E. Davis, R. C. Binning Jr. and L. Radom, *J. Chem. Phys.* 1995, **103**, 6104-6113; i) T. Clark, J. Chandrasekhar, G. W. Spitznagel and P. V. R. Schleyer, *J. Comp. Chem.* 1983, **4**, 294-301; j) M. J. Frisch, J. A. Pople and J. S. Binkley, *J. Chem. Phys.* 1984, **80**, 3265-3269.
